# Supplementary material for: Na2S-Mediated One-Pot Selective Deoxygenation of α-Hydroxyl Carbonyl Compounds including Natural Products
Source: Molecules. 2022 Jul 22;27(15):4675. doi: 10.3390/molecules27154675 (PMC9330554; doi:10.3390/molecules27154675)
Supplement: Supplementary file 1 [file molecules-27-04675-s001.zip › molecules-1810566-supplementary.pdf]

Supporting Information

A Practical and Mild Method for One-Pot Selective Dehydroxylation of α-Hydroxyl Carbonyl Compounds

Xiaobo Xu,*[a] Leyu Yan,[a] Zhi-Kai Zhang,[b] Bingqing Lu,[a] Zhuangwen Guo,[a] Mengyue Chen[a] and Zhong-Yan Cao*[b]

[a]College of Chemistry and Pharmaceutical Engineering, Huanghuai University, Zhumadian 463000, China

[b]College of Chemistry and Chemical Engineering, Henan University, Kaifeng 475004, China

Table of Contents

1. General Information..2

2. General Procedures 3

3. Characterization of Products..5

4. References..12

5. NMR Spectra 14

1. General Information

Unless otherwise noted, reactions were carried out in oven-dried glassware or sealed tube under ambient atmosphere. *N*, *N*-Dimethylformamide (DMF) was distilled from calcium hydride. Tetrahydrofuran (THF) was dried and distilled from sodium. Reactions were monitored by analytical thin-layer chromatography (TLC) on Merck silica gel 60 F_{254} plates (0.25 mm), visualized by ultraviolet light (254 nm) or by staining with ceric ammonium molybdate. ^{1}H NMR spectra were obtained on a Bruker AVANCE 400 MHz spectrometer at ambient temperature. Data were reported as follows: chemical shift on the δ scale using residual proton solvent as internal standard [δ TMS: 0.00 ppm], multiplicity (s = singlet, d = doublet, t = triplet, q = quartet, m = multiplet, dd = doublet of doublets), integration, and coupling constant (J) in hertz (Hz). ^{13}C NMR spectra were obtained with proton decoupling on a Bruker AVANCE (100 MHz) spectrometer and were reported in ppm with residual solvent for internal standard [δ 77.0 ($CHCl_3$)].

2. Experimental procedures

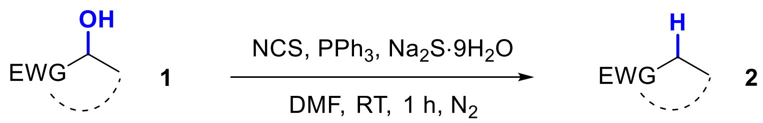

General dihydroxylation procedure: To a solution of α-hydroxyl carbonyl compounds **1** (0.5 mmol) and PPh_3 (0.5 mmol) in dry DMF (2.0 mL) under nitrogen atmosphere at room temperature, followed by the addition of NCS (0.5 mmol). The resulting reaction mixture was stirred for 0.5 h and $Na_2S \cdot 9H_2O$ (0.5 mmol) was then added to the mixture. After 0.5 h the reaction was completed, the reaction was quenched with H_2O (5 mL), and the resulting mixture was extracted with ethyl acetate (3 × 20 mL). The organic phases were combined, washed with saturated brine, dried over anhydrous Na_2SO_4, and and concentrated in vacuo. The resultant residue was purified by flash chromatography (petroleum ether/ethyl acetate = 50/1−10/1) on silica gel to afford the desired product **2**.

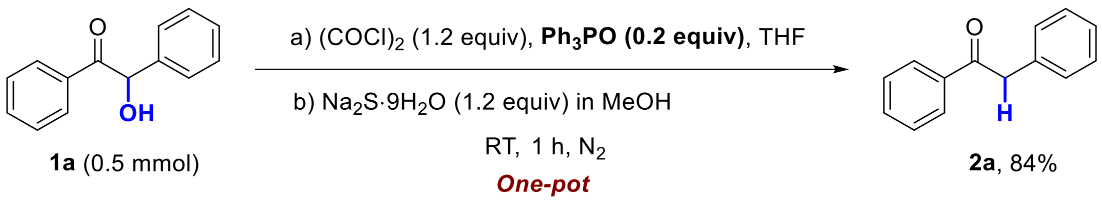

General procedure for catalytic triphenylphosphine oxide mediated reaction: At room temperature, benzoin **1a** (0.5 mmol, 106 mg) and Ph_3PO (0.2 equiv, 28 mg) were added to a dry round-bottom flask, and the gas was purged three times under a nitrogen atmosphere. Then, the solvent dry THF (2 mL) and $(COCl)_2$ (1.2 equiv, 51 μL) were added. The reaction was initiated at room temperature, and the mixture was stirred for 0.5 h, followed by the addition of $Na_2S \cdot 9H_2O$ (1.2 equiv, 144 mg) in MeOH (1 mL). After 0.5 h the reaction was completed, the reaction was quenched with H_2O (5 mL), and the resulting mixture was extracted with ethyl acetate (3 × 20 mL). The organic phases were combined, washed with saturated brine, dried over anhydrous Na_2SO_4, and

and concentrated in vacuo. The resultant residue was purified by flash chromatography (petroleum ether/ethyl acetate = 50/1−10/1) on silica gel to afford the desired product **2a** as a white solid (82 mg, 84% yield).

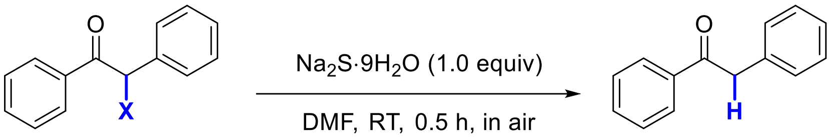

3a-3c **2a**

General procedure for Na_2S-mediated dehalogenation: To a solution of α-halogenated carbonyl compounds **3a-3c** (0.5 mmol) and $Na_2S{\cdot}9H_2O$ (1.0 equiv, 0.5 mmol) in DMF (2 mL) at room temperature. The resulting reaction mixture was stirred for 0.5 h and H_2O (5 mL) was then added to the mixture. The resulting mixture was extracted with ethyl acetate (3 × 20 mL). The organic phases were combined, washed with saturated brine, dried over anhydrous Na_2SO_4, and and concentrated in vacuo. The resultant residue was purified by flash chromatography (petroleum ether/ethyl acetate = 50/1−10/1) on silica gel to afford the desired product **2a** as a white solid.

3. Characterization of Products

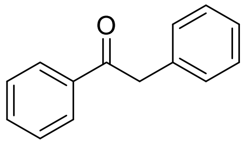

1,2-Diphenylethan-1-one (2a)[1, 2]: Known compound. Isolated yield 93% (91 mg). ^{1}H NMR (400 MHz, $CDCl_3$): *δ* (ppm) 8.08–8.06 (m, 2H), 7.59 (ddd, *J* = 6.7, 3.9, 1.2 Hz, 1H), 7.49 (dd, *J* = 10.5, 4.7 Hz, 2H), 7.40–7.34 (m, 2H), 7.33–7.28 (m, 3H), 4.33 (s, 2H); ^{13}C NMR (100 MHz, $CDCl_3$): *δ* (ppm) 197.5, 136.5, 134.4, 133.1, 129.4, 128.6, 128.5, 128.5, 126.8, 45.4.

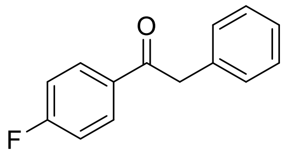

1-(4-Fluorophenyl)-2-phenylethan-1-one (2b)[2]: Known compound. Isolated yield 88% (94 mg). ^{1}H NMR (400 MHz, $CDCl_3$): *δ* (ppm) 8.09–8.06 (m, 2H), 7.37 (dd, *J* = 8.0, 6.8 Hz, 2H), 7.30 (d, *J* = 6.9 Hz, 3H), 7.15 (t, *J* = 8.6 Hz, 2H), 4.29 (s, 2H); ^{13}C NMR (100 MHz, $CDCl_3$): *δ* (ppm) 195.9, 166.9, 164.4, 134.3, 132.9, 132.9, 131.2, 131.1, 129.3, 128.6, 126.9, 115.7, 115.5, 45.4.

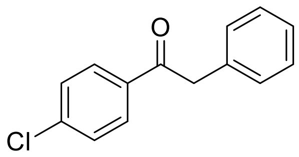

1-(4-Chlorophenyl)-2-phenylethan-1-one (2c)[1, 2]: Known compound. Isolated yield 90% (104 mg). ^{1}H NMR (400 MHz, $CDCl_3$): *δ* (ppm) 7.99–7.96 (m, 2H), 7.46–7.44 (m, 2H), 7.38–7.35 (m, 2H), 7.31–7.28 (m, 3H), 4.28 (s, 2H); ^{13}C NMR (100 MHz, $CDCl_3$): *δ* (ppm) 196.4, 139.5, 134.8, 134.1, 133.0, 129.3, 128.9, 128.7, 127.0, 45.6.

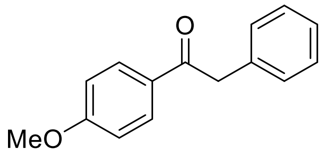

1-(4-Methoxyphenyl)-2-phenylethan-1-one (2d)[2]: Known compound. Isolated yield 95% (107 mg). ^{1}H NMR (400 MHz, $CDCl_3$): δ (ppm) 8.05–8.02 (m, 2H), 7.37–7.27 (m, 5H), 6.97–6.95 (m, 2H), 4.26 (s, 2H), 3.87 (s, 3H); ^{13}C NMR (100 MHz, $CDCl_3$): δ (ppm) 196.1, 163.4, 134.9, 130.8, 129.5, 129.3, 128.5, 126.7, 113.7, 55.3, 45.1.

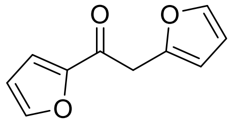

1,2-Di(furan-2-yl)ethan-1-one (2e)[3]: Known compound. Isolated yield 84% (74 mg). ^{1}H NMR ($CDCl_3$, 400 MHz): δ (ppm) 8.60 (d, J = 7.9 Hz, 1H), 8.28 (d, J = 8.1 Hz, 1H), 8.03 (t, J = 7.6 Hz, 1H), 7.71 (dt, J = 8.6, 4.0 Hz, 3H), 7.42 (dd, J = 5.3, 1.9 Hz, 3H); ^{13}C NMR ($CDCl_3$, 100 MHz): δ (ppm) 145.08, 144.56, 143.84, 135.36, 131.54, 131.41, 129.25, 126.77, 126.28, 125.30.

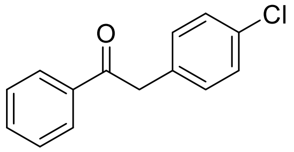

2-(4-Chlorophenyl)-1-phenylethan-1-one (2f)[2]: Known compound. Isolated yield 91% (105 mg). ^{1}H NMR ($CDCl_3$, 400 MHz): δ (ppm) 8.04–8.02 (m, 2H), 7.62–7.58 (m, 1H), 7.50 (dd, J = 8.4, 7.0 Hz, 2H), 7.34–7.32 (m, 2H), 7.23–7.21 (m, 2H), 4.29 (s, 2H); ^{13}C NMR ($CDCl_3$, 100 MHz): δ (ppm) 197.0, 136.3, 133.3, 132.9, 132.8, 130.9, 128.7, 128.7, 128.4, 44.6.

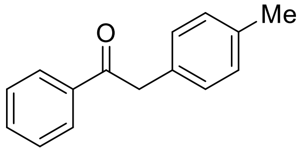

1-Phenyl-2-(p-tolyl)ethan-1-one (2g)[2]: Known compound. Isolated yield 92% (97 mg). ^{1}H NMR (CDCl$_3$, 400 MHz): δ (ppm) 8.07–8.05 (m, 2H), 7.59–7.57 (m, 1H), 7.51–7.47 (m, 2H), 7.23–7.17 (m, 4H), 4.29 (s, 2H), 2.37 (s, 3H); ^{13}C NMR (CDCl$_3$, 100 MHz): δ (ppm) 197.7, 136.5, 136.4, 133.0, 131.7, 129.3, 129.2, 128.5, 45.0, 21.0.

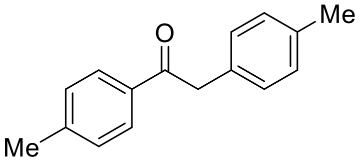

1,2-Di-p-tolylethan-1-one (2h)[1]: Known compound. Isolated yield 94% (105 mg). ^{1}H NMR (CDCl$_3$, 400 MHz): δ (ppm) 7.83–7.81 (m, 2H), 7.15 (d, J = 8.2 Hz, 2H), 7.08–7.02 (m, 4H), 4.12 (s, 2H), 2.30 (s, 3H), 2.23 (s, 3H); ^{13}C NMR (CDCl$_3$, 100 MHz): δ (ppm) 197.4, 143.8, 136.3, 134.1, 131.6, 129.3, 129.2, 129.2, 128.7, 45.0, 21.6, 21.0.

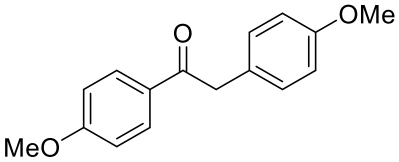

1,2-Bis(4-methoxyphenyl)ethan-1-one (2i)[1]: Known compound. Isolated yield 90% (115 mg). ^{1}H NMR (CDCl$_3$, 400 MHz): δ (ppm) 8.03–8.01 (m, 2H), 7.22–7.20 (m, 2H), 6.95–6.94 (m, 2H), 6.90–6.87 (m, 2H), 4.19 (s, 2H), 3.86 (s, 3H), 3.79 (s, 3H); ^{13}C NMR (CDCl$_3$, 100 MHz): δ (ppm) 196.4, 163.3, 158.3, 130.7, 130.2, 129.5, 126.8, 113.9, 113.6, 55.3, 55.0, 44.2.

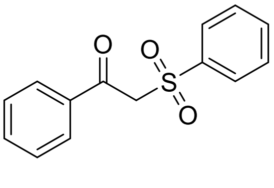

1-Phenyl-2-(phenylsulfonyl)ethan-1-one (2j)[4]: Known compound. Isolated yield 82% (107 mg). ^{1}H NMR (CDCl$_3$, 400 MHz): δ (ppm) 7.94–7.89 (m, 4H), 7.67–7.61 (m, 2H),

7.59–7.48 (m, 2H), 7.46 (t, J = 7.9 Hz, 2H), 4.77 (s, 2H); ^{13}C NMR (CDCl$_3$, 100 MHz): δ (ppm) 187.9, 138.7, 135.6, 134.2, 134.1, 129.1, 129.0, 128.7, 128.4, 63.2.

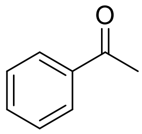

Acetophenone (2k)[3, 5]: Known compound. Isolated yield 65% (39 mg). ^{1}H NMR (CDCl$_3$, 400 MHz): δ (ppm) 7.93–7.91 (m, 2H), 7.54–7.50 (m, 1H), 7.44–7.40 (m, 2H), 2.55 (s, 3H); ^{13}C NMR (CDCl$_3$, 100 MHz): δ (ppm) 197.6, 136.7, 132.7, 128.2, 127.9, 26.2.

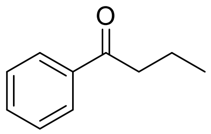

1-Phenylbutan-1-one (2l)[5]: Known compound. Isolated yield 94% (70 mg). ^{1}H NMR (CDCl$_3$, 400 MHz): δ (ppm) 7.98–7.96 (m, 2H), 7.57–7.53 (m, 1H), 7.48–7.44 (m, 2H), 2.95 (t, J = 7.3 Hz, 2H), 1.78 (p, J = 7.4 Hz, 2H), 1.02 (t, J = 7.4 Hz, 3H); ^{13}C NMR (CDCl$_3$, 100 MHz): δ (ppm) 200.2, 137.0, 132.7, 128.4, 127.9, 40.4, 17.7, 13.8.

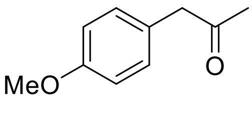

1-(4-Methoxyphenyl)propan-2-one (2m)[1]: Known compound. Isolated yield 95% (78 mg). ^{1}H NMR (CDCl$_3$, 400 MHz): δ (ppm) 7.13–7.10 (m, 2H), 6.88–6.86 (m, 2H), 3.78 (s, 3H), 3.62 (s, 2H), 2.13 (s, 3H); ^{13}C NMR (CDCl$_3$, 100 MHz): δ (ppm) 206.4, 158.4, 130.1, 126.1, 113.9, 54.9, 49.7, 28.8.

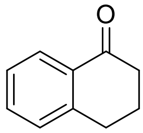

3,4-Dihydronaphthalen-1(2H)-one (2n)[6]: Known compound. Isolated yield 88% (64 mg). ^{1}H NMR ($CDCl_3$, 400 MHz): δ (ppm) 7.99 (dd, J = 7.8, 1.5 Hz, 1H), 7.41 (td, J = 7.5, 1.5 Hz, 1H), 7.25 (t, J = 7.6 Hz, 1H), 7.20 (d, J = 7.7 Hz, 1H), 2.91 (t, J = 6.1 Hz, 2H), 2.60 (dd, J = 7.3, 5.8 Hz, 2H), 2.08 (p, J = 6.4 Hz, 2H); ^{13}C NMR ($CDCl_3$, 100 MHz): δ (ppm) 197.9, 144.2, 133.1, 132.3, 128.5, 126.8, 126.3, 38.8, 29.4, 23.0.

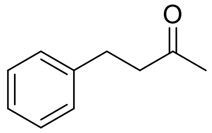

4-Phenylbutan-2-one (2o)[7]: Known compound. Isolated yield 86% (64 mg). ^{1}H NMR ($CDCl_3$, 400 MHz): δ (ppm) 7.33–7.30 (m, 2H), 7.23 (td, J = 5.9, 1.9 Hz, 3H), 2.93 (t, J = 7.6 Hz, 2H), 2.80–2.76 (m, 2H), 2.16 (s, 3H); ^{13}C NMR ($CDCl_3$, 100 MHz): δ (ppm) 207.6, 140.8, 128.3, 128.1, 125.9, 44.9, 29.8, 29.5.

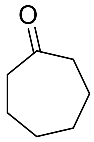

Cycloheptanone (2p)[8]: Known compound. Isolated yield 75% (42 mg). ^{1}H NMR ($CDCl_3$, 400 MHz): δ (ppm) 2.41–2.38 (m, 4H), 1.62–1.56 (m, 4H); ^{13}C NMR ($CDCl_3$, 100 MHz): δ (ppm) 214.9, 43.5, 30.1, 24.0.

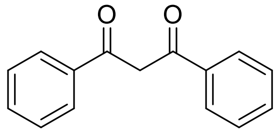

1,3-Diphenylpropane-1,3-dione (2q)[9]: Known compound. Isolated yield 89% (100 mg). ^{1}H NMR ($CDCl_3$, 400 MHz): δ (ppm) 8.04–8.02 (m, 4H), 7.61–7.54 (m, 2H), 7.52 (dd, J = 8.3, 6.5 Hz, 4H), 6.90 (s, 1H); ^{13}C NMR ($CDCl_3$, 100 MHz): δ (ppm) 194.3, 185.7, 135.5, 132.4, 128.6, 127.1, 93.1.

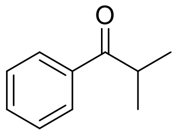

2-Methyl-1-phenylpropan-1-one (2r)[3]: Known compound. Isolated yield 73% (54 mg). ^{1}H NMR ($CDCl_3$, 400 MHz): δ (ppm) 7.96–7.94 (m, 2H), 7.54–7.50 (m, 1H), 7.48–7.42 (m, 2H), 3.53 (dq, *J* = 13.7, 6.8 Hz, 1H), 1.21 (d, *J* = 7.0 Hz, 6H); ^{13}C NMR ($CDCl_3$, 100 MHz): δ (ppm) 204.1, 136.0, 136.2, 132.6, 128.4, 128.1, 35.1, 18.9.

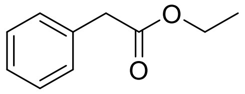

Ethyl 2-phenylacetate (2s)[10]: Known compound. Isolated yield 81% (66 mg). ^{1}H NMR ($CDCl_3$, 400 MHz): δ (ppm) 7.39 (dt, *J* = 6.8, 1.4 Hz, 1H), 7.35–7.31 (m, 4H), 4.20 (q, *J* = 7.1 Hz, 2H), 3.66 (s, 2H), 1.30 (t, *J* = 7.1 Hz, 3H); ^{13}C NMR ($CDCl_3$, 100 MHz): δ (ppm) 171.3, 134.0, 129.0, 128.3, 126.8, 60.6, 41.2, 13.9.

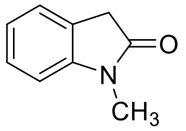

1-Methylindolin-2-one (2t)[11]: Known compound. Isolated yield 87% (64 mg). ^{1}H NMR ($CDCl_3$, 400 MHz): δ (ppm) 7.22–7.14 (m, 2H), 6.95 (td, *J* = 7.6, 1.1 Hz, 1H), 6.73 (d, *J* = 7.8 Hz, 1H), 3.42 (s, 2H), 3.12 (s, 3H); ^{13}C NMR ($CDCl_3$, 100 MHz): δ (ppm) 175.0, 145.1, 127.8, 124.4, 124.2, 122.2, 108.0, 35.6, 26.0.

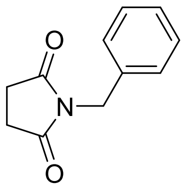

1-Benzylpyrrolidine-2,5-dione (2u)[12]: Known compound. Isolated yield 76% (72 mg). ^{1}H NMR ($CDCl_3$, 400 MHz): δ (ppm) 7.39–7.38 (m, 2H), 7.33–7.27 (m, 3H), 4.64

(s, 2H), 2.67 (s, 4H); ^{13}C NMR ($CDCl_3$, 100 MHz): δ (ppm) 176.7, 135.7, 128.7, 128.5, 127.8, 42.2, 28.0.

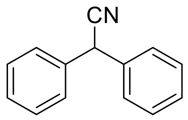

2,2-Diphenylacetonitrile (2v)[13]: Known compound. Isolated yield 78% (75 mg). ^{1}H NMR ($CDCl_3$, 400 MHz): δ (ppm) 7.42–7.38 (m, 10H), 5.19 (s, 1H); ^{13}C NMR ($CDCl_3$, 100 MHz): δ (ppm) 135.8, 129.0, 128.1, 127.6, 119.6, 42.4.

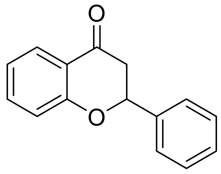

Flavanone (2w)[14]: Known compound. Isolated yield 87% (97 mg). ^{1}H NMR ($CDCl_3$, 400 MHz): δ (ppm) 7.97 (dd, J = 8.0, 1.8 Hz, 1H), 7.56–7.42 (m, 6H), 7.10–7.07 (m, 2H), 5.51 (dd, J = 13.4, 2.9 Hz, 1H), 3.12 (dd, J = 16.9, 13.3 Hz, 1H), 2.92 (dd, J = 16.9, 2.9 Hz, 1H); ^{13}C NMR ($CDCl_3$, 100 MHz): δ (ppm) 191.9, 161.5, 138.7, 136.1, 128.8, 128.7, 127.0, 126.1, 121.6, 120.9, 118.1, 79.5, 44.6.

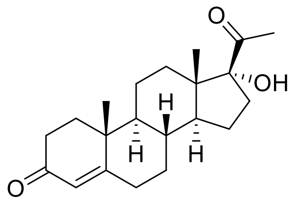

Hydroxyprogesterone (2x)[15]: Known compound. Isolated yield 53% (87 mg). ^{1}H NMR ($CDCl_3$, 400 MHz): δ (ppm) 5.70 (d, J = 1.8 Hz, 1H), 3.00 (s, 1H), 2.66 (ddd, J = 14.7, 11.4, 2.9 Hz, 1H), 2.39–2.26 (m, 4H), 2.24 (s, 3H), 2.02 (ddd, J = 13.4, 5.0, 3.3 Hz, 1H), 1.99–1.53 (m, 8H), 1.43–1.30 (m, 3H), 1.16 (s, 3H), 0.95–0.94 (m, 2H), 0.71 (s, 3H); ^{13}C NMR ($CDCl_3$, 100 MHz): 211.5, 199.6, 171.2, 171.2, 123.8, 89.8, 53.2, 49.9, 47.9, 38.5, 35.6, 35.4, 33.8, 33.3, 32.7, 31.9, 30.0, 27.7, 23.8, 20.4, 17.3, 15.2.

4. References

1. Thopate, Y.; Singh, R.; Rastogi, S. K.; Sinha, A. K. A highly regioselective and practical synthesis of α-aryl ketones under a cooperative cascade effect of an ionic liquid and tetrabutylammonium fluoride. *Asian J. Org. Chem.* **2019**, *8*, 2017-2022.
2. Luo, M.; Zhang, Y.; Fang, P.; Li, Y.; Qi, C.; Li, Y.; Shen, R.; Cheng, K.; Wang, H. H_2O_2-mediated room temperature synthesis of 2-arylacetophenones from arylhydrazines and vinyl azides in water. *Org. Biomol. Chem.* **2022**, *20*, 630-635.
3. Zhang, J.; Yang, J.-D.; Cheng, J.-P. Diazaphosphinyl radical-catalyzed deoxygenation of α-carboxy ketones: a new protocol for chemo-selective C–O bond scission via mechanism regulation. *Chem. Sci.* **2020**, *11*, 8476-8481.
4. Chikunova, E. I.; Kukushkin, V. Y.; Dubovtsev, A. Y. Atom-economic synthesis of β-ketosulfones based on gold-catalyzed highly regioselective hydration of alkynylsulfones. *Green Chem.* **2022**, *24*, 3314-3320.
5. Zhu, X.; Liu, Y.; Liu, C.; Yang, H.; Fu, H. Light and oxygen-enabled sodium trifluoromethanesulfinate-mediated selective oxidation of C–H bonds. *Green Chem.* **2020**, *22*, 4357-4363.
6. Santra, S. K.; Szpilman, A. M. Visible-spectrum solar-light-mediated benzylic c–h oxygenation using 9,10-dibromoanthracene as an initiator. *J. Org. Chem.* **2021**, *86*, 1164-1171.
7. Xu, L.; Sun, S.; Zhang, X.; Gao, H.; Wang, W. Study on the selective hydrogenation of isophorone. *RSC Adv.* **2021**, *11*, 4465-4471.
8. Zhong, J.-J.; To, W.-P.; Liu, Y.; Lu, W.; Che, C.-M. Efficient acceptorless photo-dehydrogenation of alcohols and N-heterocycles with binuclear platinum(ii) diphosphite complexes. *Chem. Sci.* **2019**, *10*, 4883-4889.
9. Zhu, Z.; Tang, X.; Li, J.; Li, X.; Wu, W.; Deng, G.; Jiang, H. Synthesis of enaminones via copper-catalyzed decarboxylative coupling reaction under

redox-neutral conditions. *Chem. Commun.* **2017**, *53*, 3228-3231.

10. Nguyen, T. V.; Lyons, D. J. M. A novel aromatic carbocation-based coupling reagent for esterification and amidation reactions. *Chem. Commun.* **2015**, *51*, 3131-3134.

11. Zhou, Z.-Z.; Zhao, J.-H.; Gou, X.-Y.; Chen, X.-M.; Liang, Y.-M. Visible-light-mediated hydrodehalogenation and Br/D exchange of inactivated aryl and alkyl halides with a palladium complex. *Org. Chem. Front.* **2019**, *6*, 1649-1654.

12. Duchemin, N.; Buccafusca, R.; Daumas, M.; Ferey, V.; Arseniyadis, S. A unified strategy for the synthesis of difluoromethyl- and vinylfluoride-containing scaffolds. *Org. Lett.* **2019**, *21*, 8205-8210.

13. Liu, R. Y.; Bae, M.; Buchwald, S. L. Mechanistic insight facilitates discovery of a mild and efficient copper-catalyzed dehydration of primary amides to nitriles using hydrosilanes. *J. Am. Chem. Soc.* **2018**, *140*, 1627-1631.

14. He, X.; Xie, M.; Li, R.; Choy, P. Y.; Tang, Q.; Shang, Y.; Kwong, F. Y. Organocatalytic approach for assembling flavanones via a cascade 1,4-conjugate addition/oxa-michael addition between propargylamines with water. *Org. Lett.* **2020**, *22*, 4306-4310.

15. Epifanov, M.; Mo, J. Y.; Dubois, R.; Yu, H.; Sammis, G. M. One-pot deoxygenation and substitution of alcohols mediated by sulfuryl fluoride. *J. Org. Chem.* **2021**, *86*, 3768-3777.

5. NMR Spect

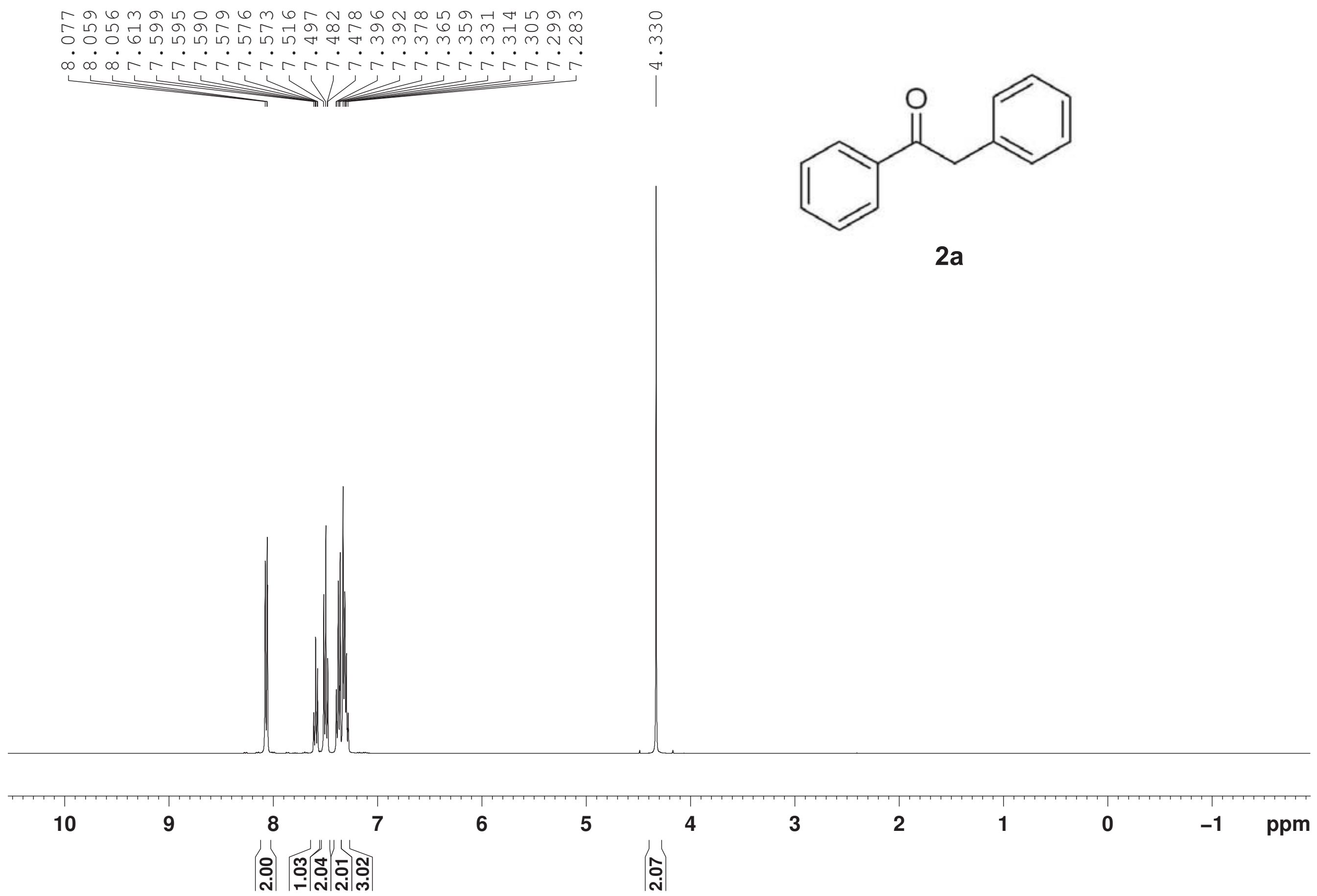
8.077
8.059
8.056
7.613
7.599
7.595
7.590
7.579
7.576
7.573
7.516
7.497
7.482
7.478
7.396
7.392
7.378
7.365
7.359
7.331
7.314
7.305
7.299
7.283
4.330
2a
10 9 8 7 6 5 4 3 2 1 0 –1 ppm
2.00
1.03
2.04
2.01
3.02
2.07

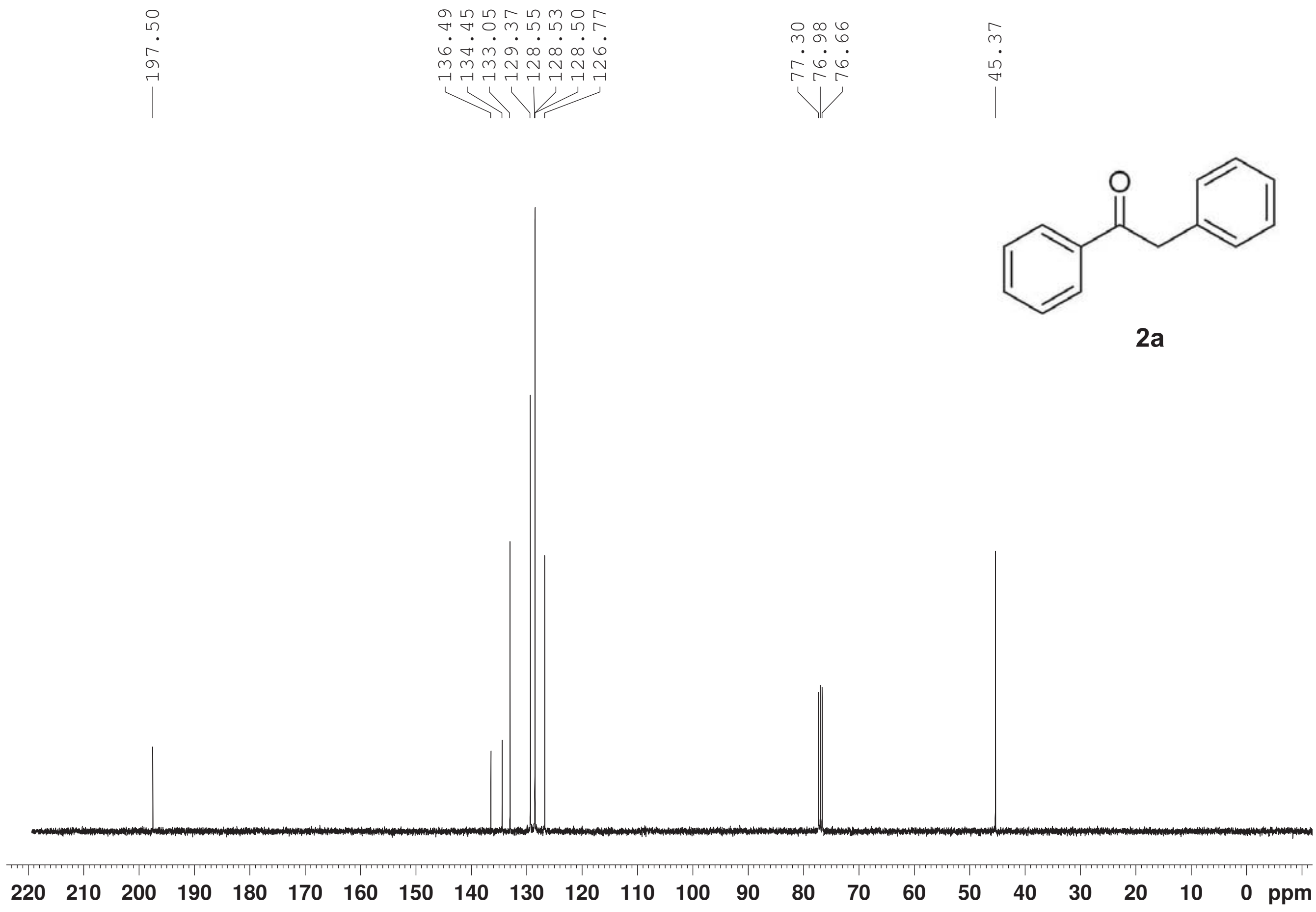
197.50
136.49
134.45
133.05
129.37
128.55
128.53
128.50
126.77
77.30
76.98
76.66
45.37
2a
220 210 200 190 180 170 160 150 140 130 120 110 100 90 80 70 60 50 40 30 20 10 0 ppm

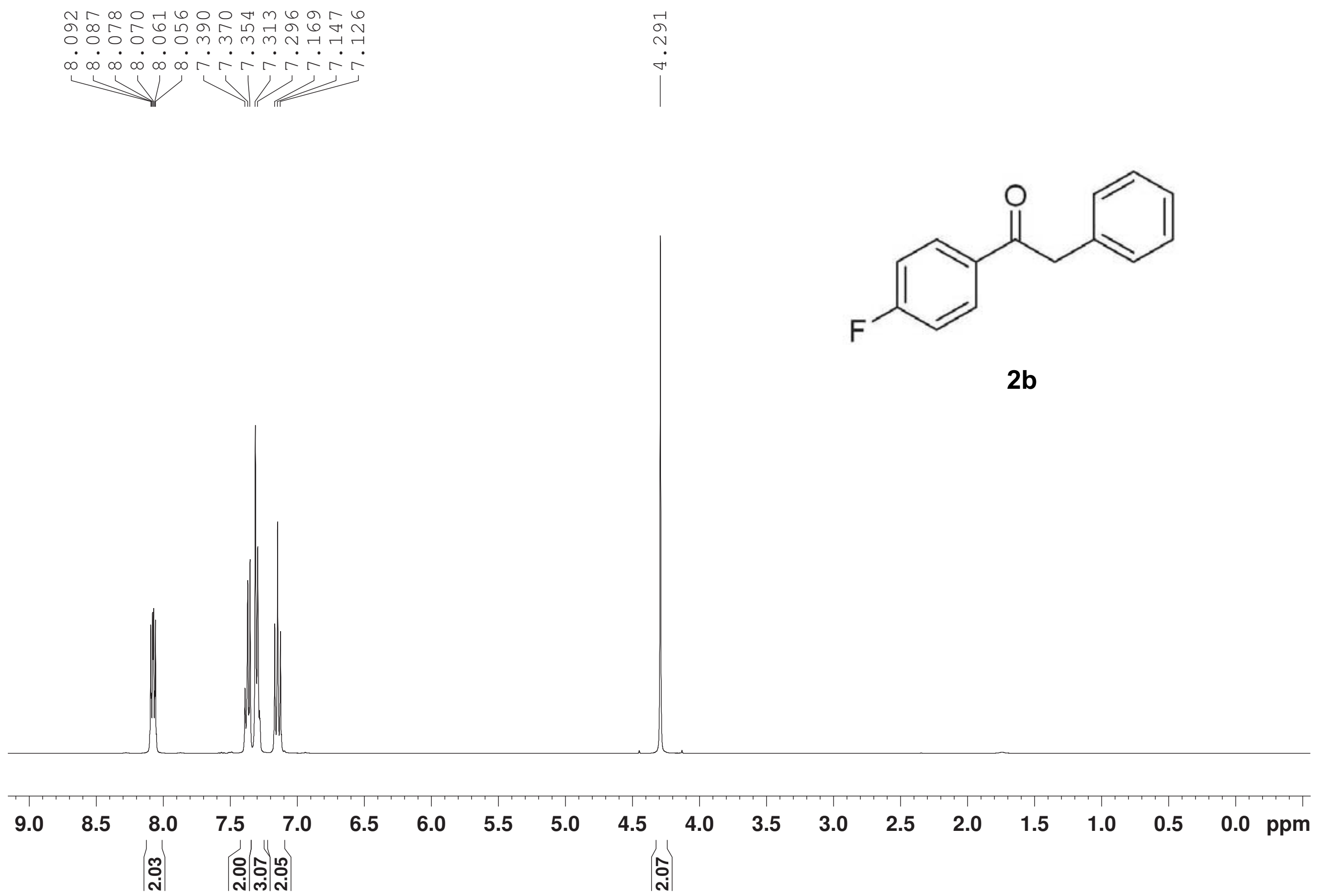
8.092
8.087
8.078
8.070
8.061
8.056
7.390
7.370
7.354
7.313
7.296
7.169
7.147
7.126
4.291
2b
9.0 8.5 8.0 7.5 7.0 6.5 6.0 5.5 5.0 4.5 4.0 3.5 3.0 2.5 2.0 1.5 1.0 0.5 0.0 ppm
2.03
2.00
3.07
2.05
2.07

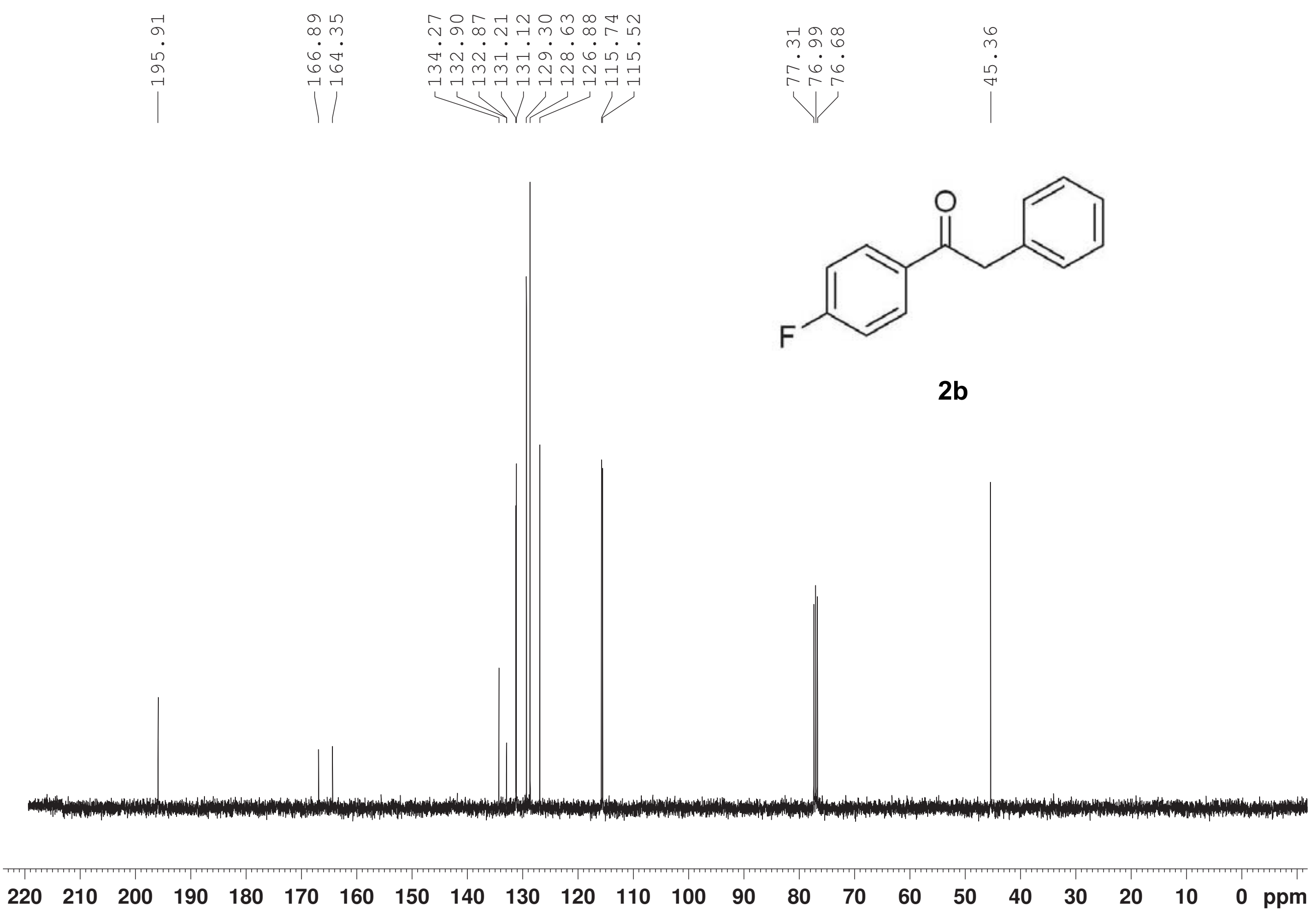
195.91
166.89
164.35
134.27
132.90
132.87
131.21
131.12
129.30
128.63
126.88
115.74
115.52
77.31
76.99
76.68
45.36
2b
220 210 200 190 180 170 160 150 140 130 120 110 100 90 80 70 60 50 40 30 20 10 0 ppm

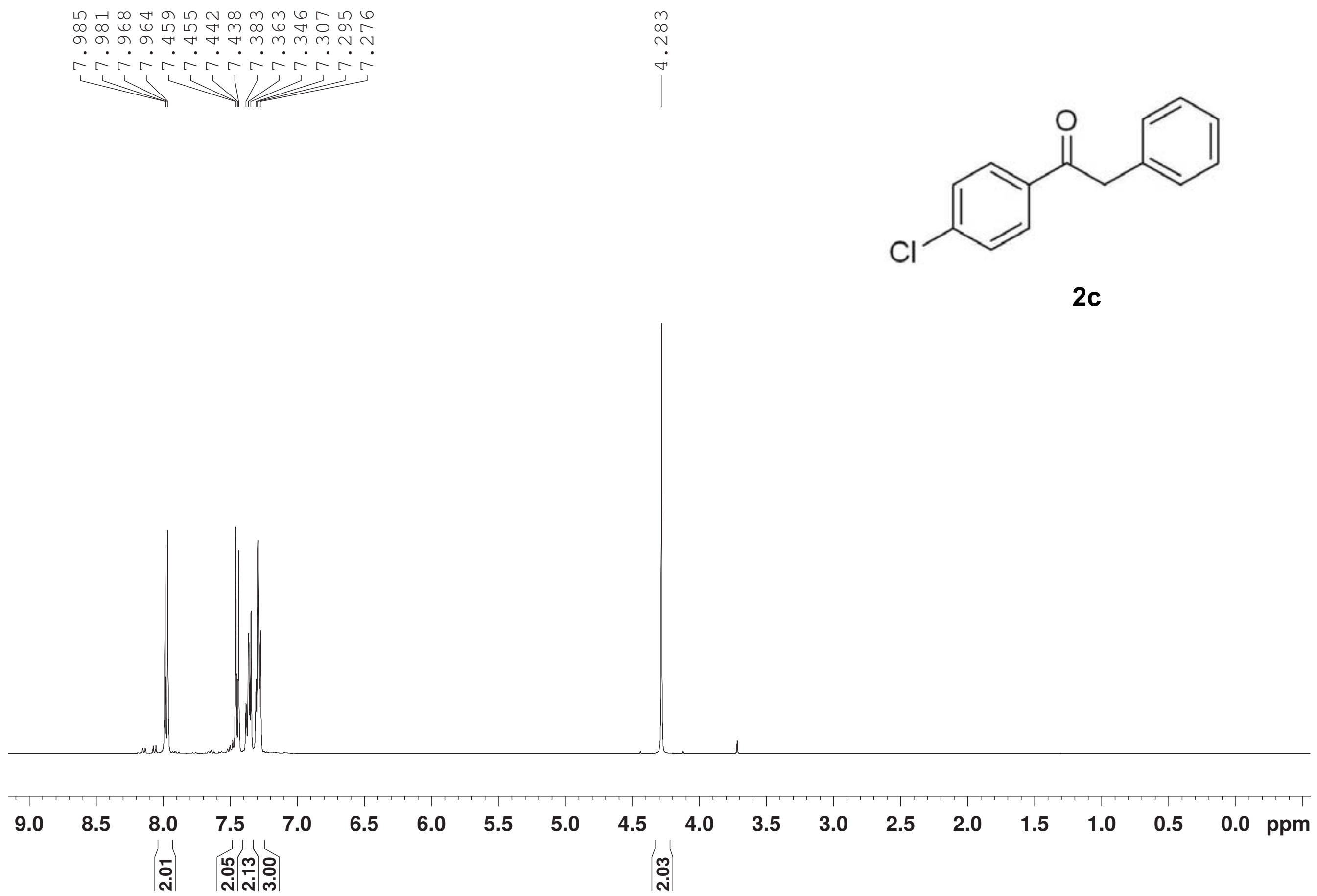
7.985
7.981
7.968
7.964
7.459
7.455
7.442
7.438
7.383
7.363
7.346
7.307
7.295
7.276
4.283
O
Cl
2c
9.0 8.5 8.0 7.5 7.0 6.5 6.0 5.5 5.0 4.5 4.0 3.5 3.0 2.5 2.0 1.5 1.0 0.5 0.0 ppm
2.01
2.05
2.13
3.00
2.03

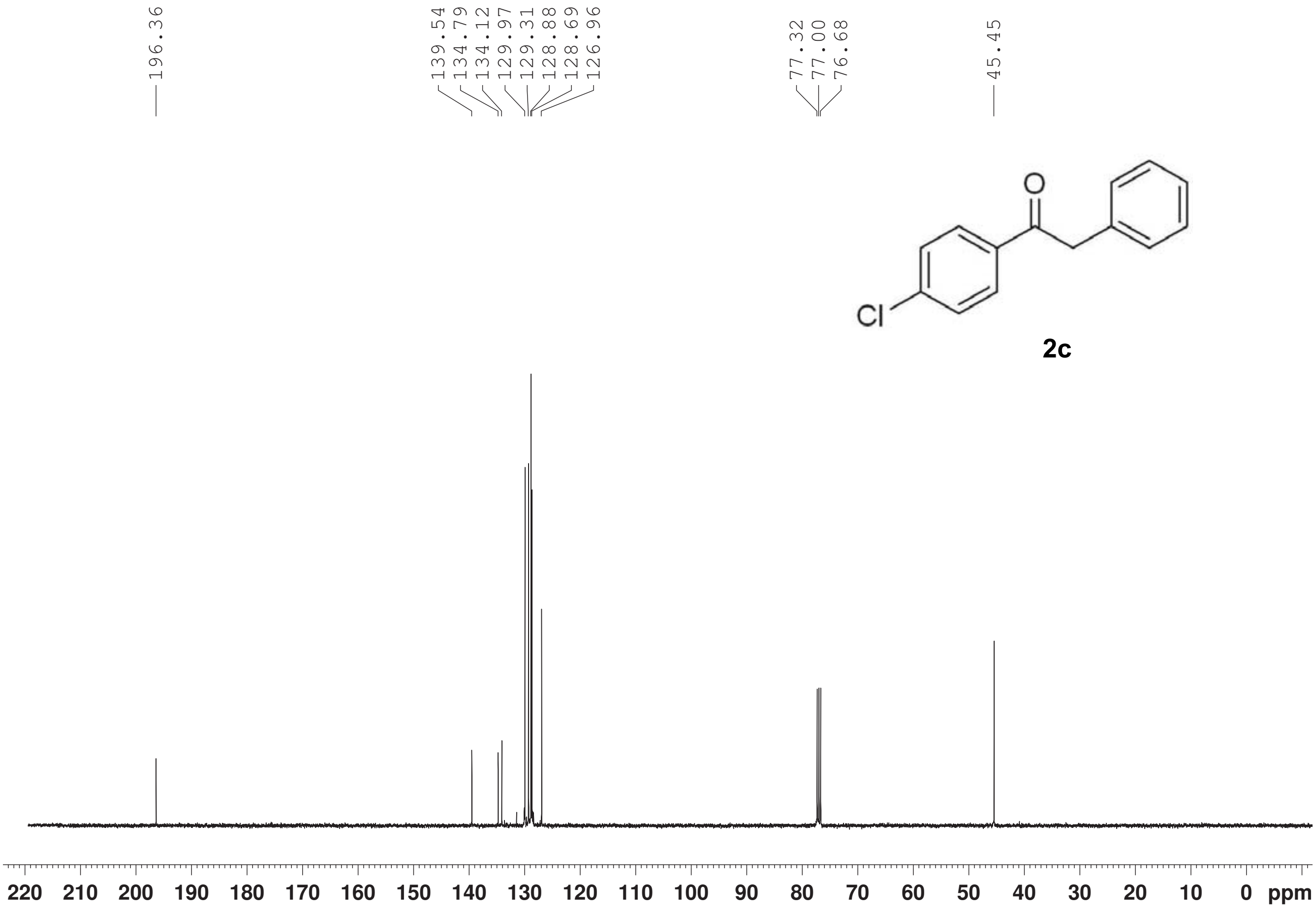
196.36
139.54
134.79
134.12
129.97
129.31
128.88
128.69
126.96
77.32
77.00
76.68
45.45
O
Cl
2c
220 210 200 190 180 170 160 150 140 130 120 110 100 90 80 70 60 50 40 30 20 10 0 ppm

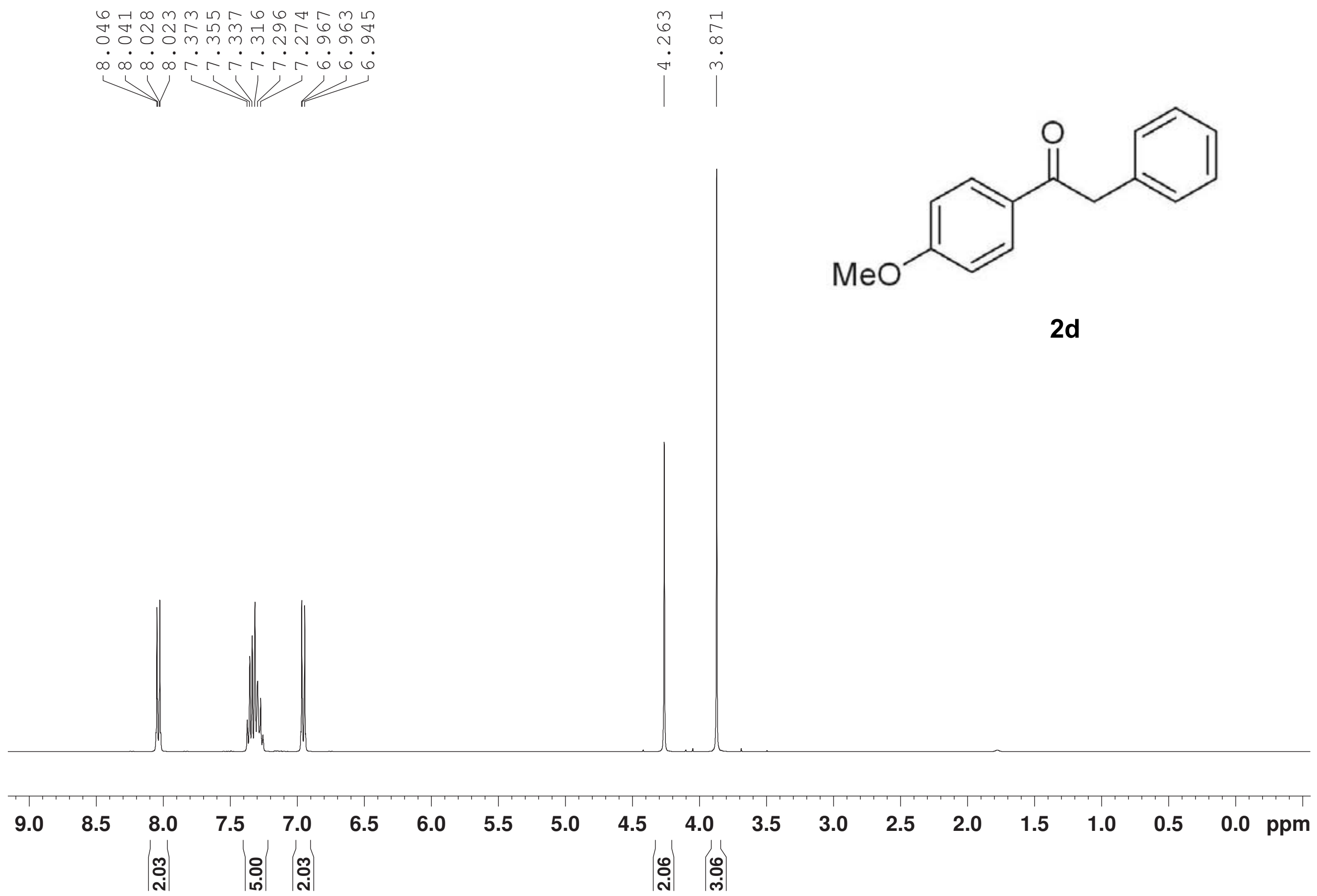
8.046
8.041
8.028
8.023
7.373
7.355
7.337
7.316
7.296
7.274
6.967
6.963
6.945
4.263
3.871
MeO
O
2d
9.0 8.5 8.0 7.5 7.0 6.5 6.0 5.5 5.0 4.5 4.0 3.5 3.0 2.5 2.0 1.5 1.0 0.5 0.0 ppm
2.03
5.00
2.03
2.06
3.06

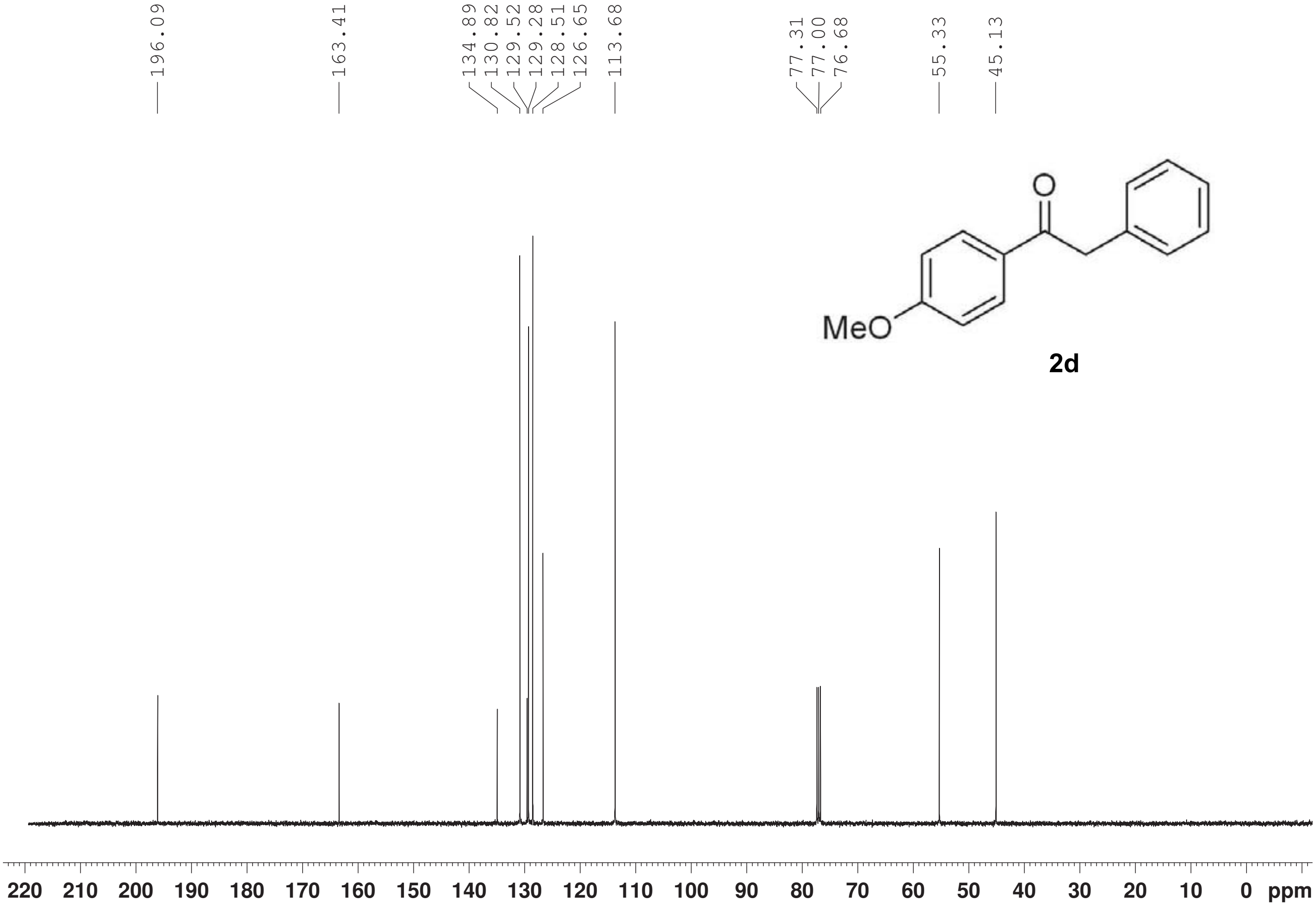
196.09
163.41
134.89
130.82
129.52
129.28
128.51
126.65
113.68
77.31
77.00
76.68
55.33
45.13
MeO
O
2d
220 210 200 190 180 170 160 150 140 130 120 110 100 90 80 70 60 50 40 30 20 10 0 ppm

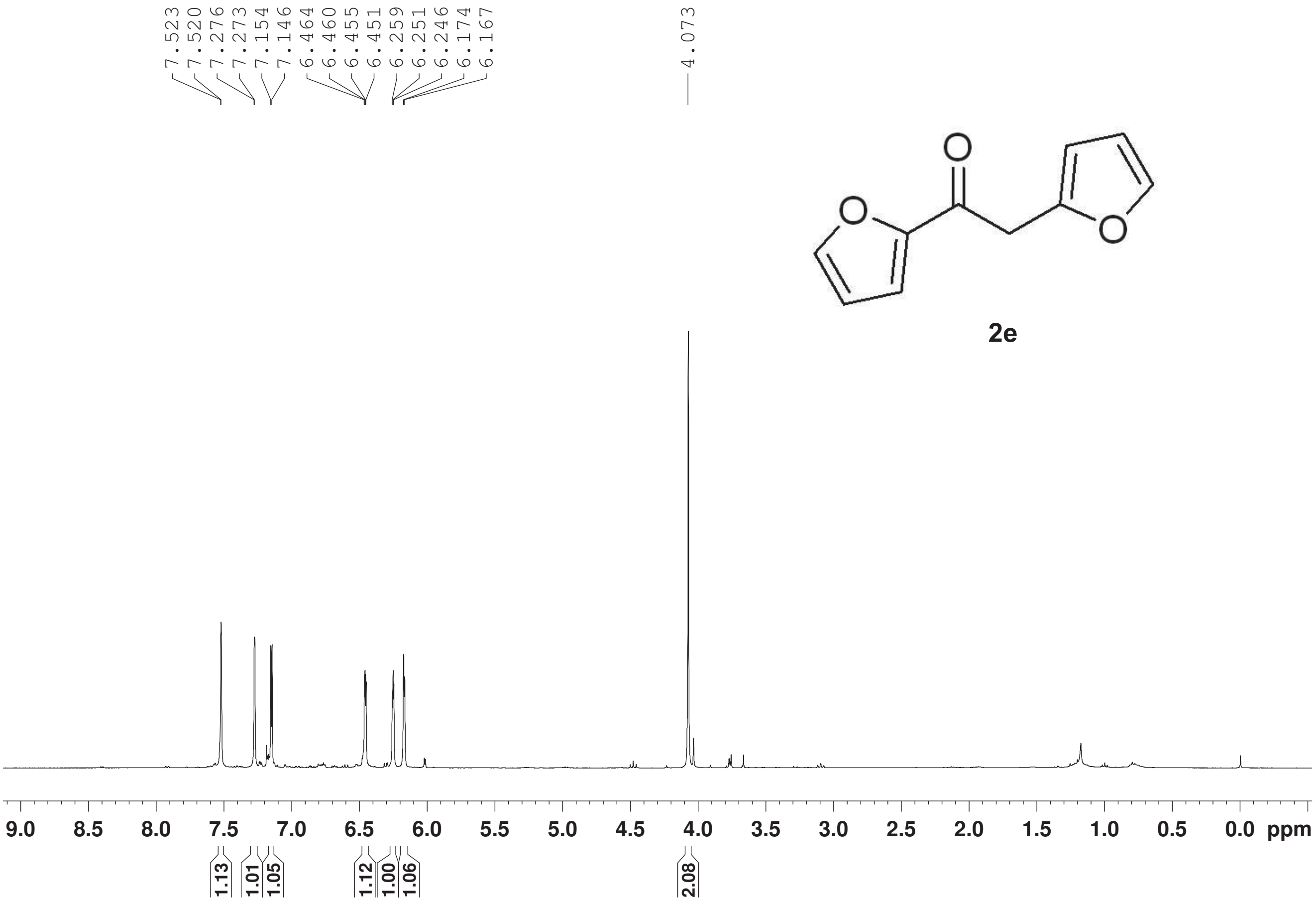
7.523
7.520
7.276
7.273
7.154
7.146
6.464
6.460
6.455
6.451
6.259
6.251
6.246
6.174
6.167
4.073
2e
1.13
1.01
1.05
1.12
1.00
1.06
2.08
9.0 8.5 8.0 7.5 7.0 6.5 6.0 5.5 5.0 4.5 4.0 3.5 3.0 2.5 2.0 1.5 1.0 0.5 0.0 ppm

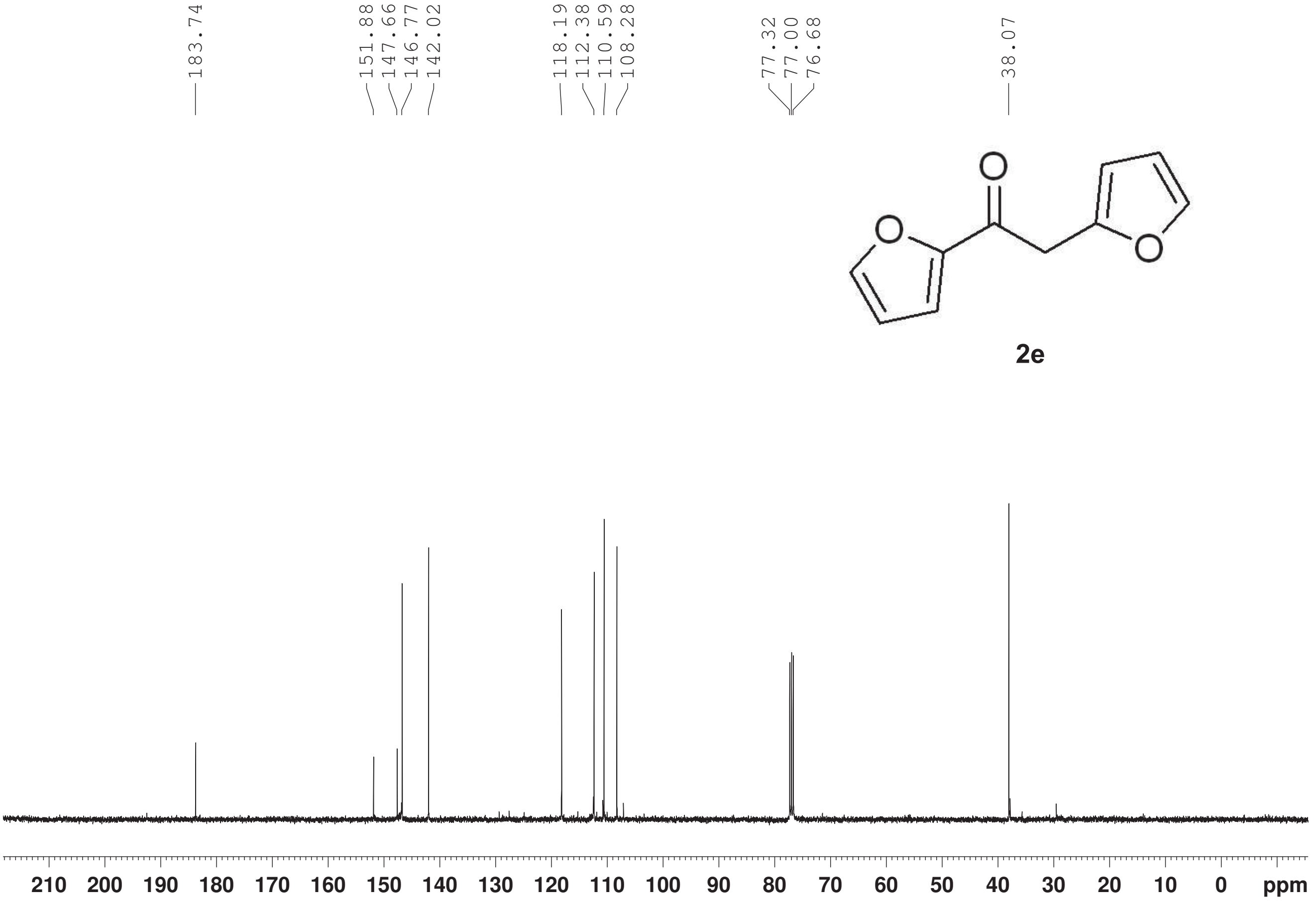
183.74
151.88
147.66
146.77
142.02
118.19
112.38
110.59
108.28
77.32
77.00
76.68
38.07
2e
210 200 190 180 170 160 150 140 130 120 110 100 90 80 70 60 50 40 30 20 10 0 ppm

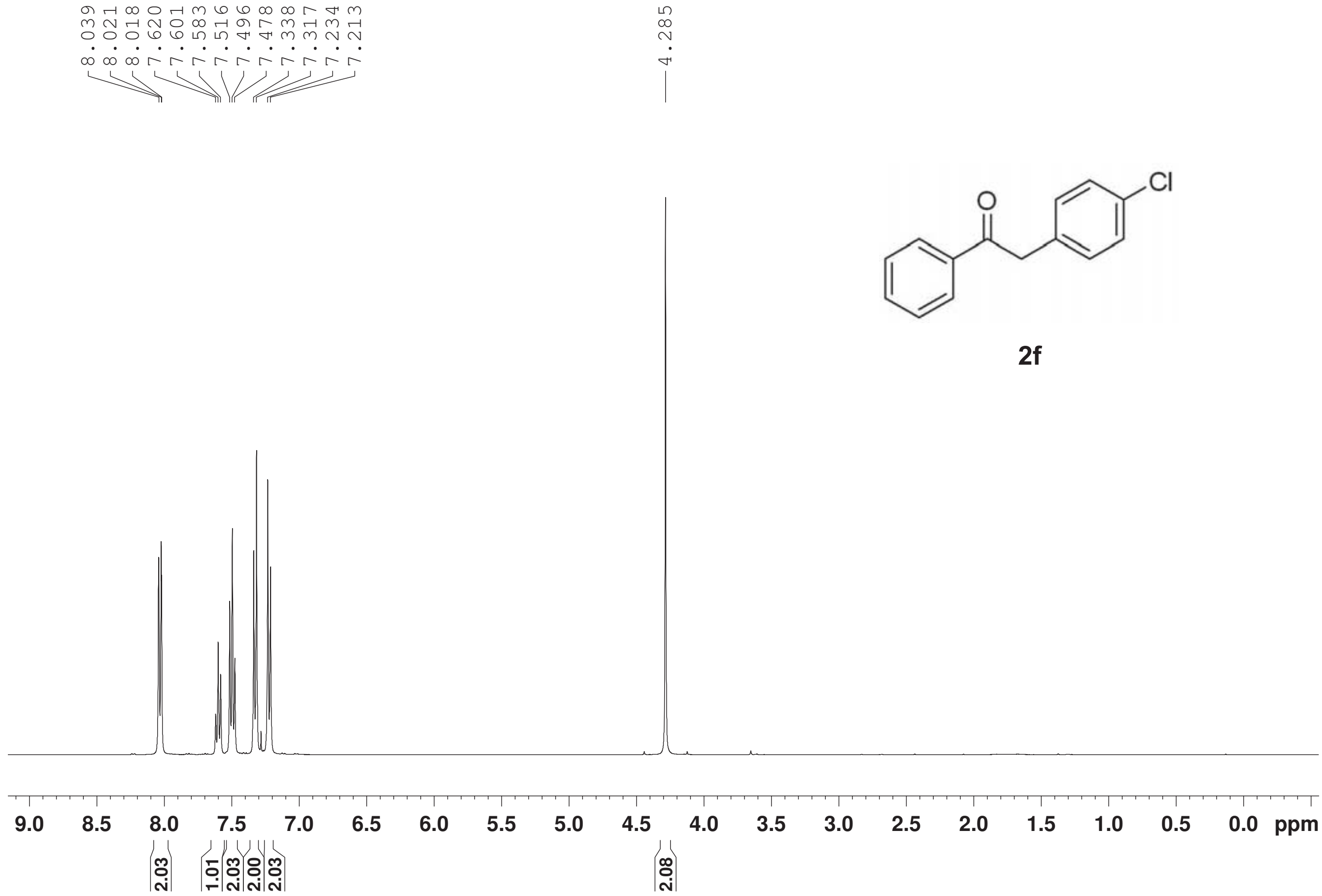
8.039 8.021 8.018 7.620 7.601 7.583 7.516 7.496 7.478 7.338 7.317 7.234 7.213
4.285
Cl
O
2f
9.0 8.5 8.0 7.5 7.0 6.5 6.0 5.5 5.0 4.5 4.0 3.5 3.0 2.5 2.0 1.5 1.0 0.5 0.0 ppm
2.03 1.01 2.03 2.00 2.03 2.08

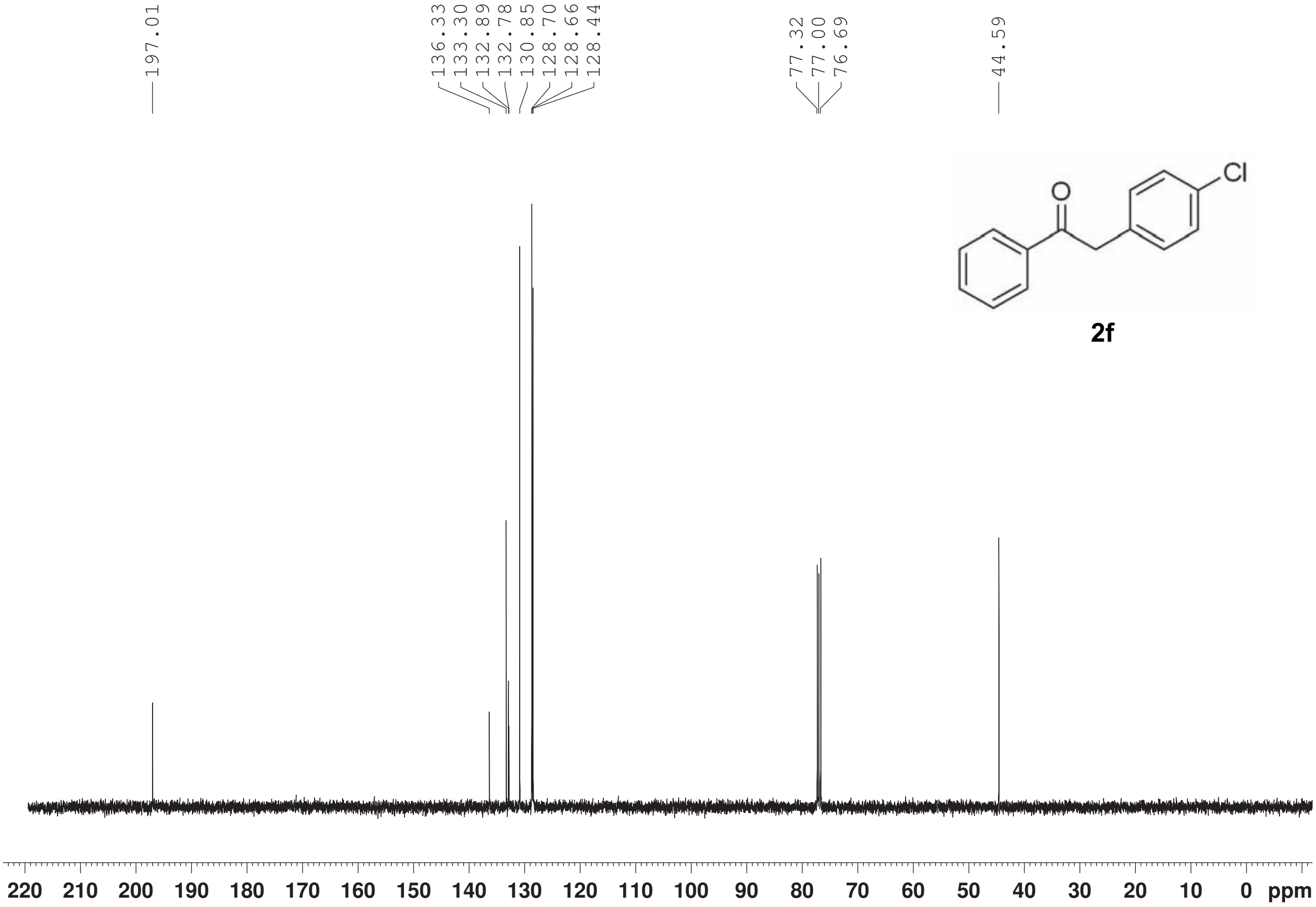
197.01
136.33 133.30 132.89 132.78 130.85 128.70 128.66 128.44
77.32 77.00 76.69
44.59
Cl
O
2f
220 210 200 190 180 170 160 150 140 130 120 110 100 90 80 70 60 50 40 30 20 10 0 ppm

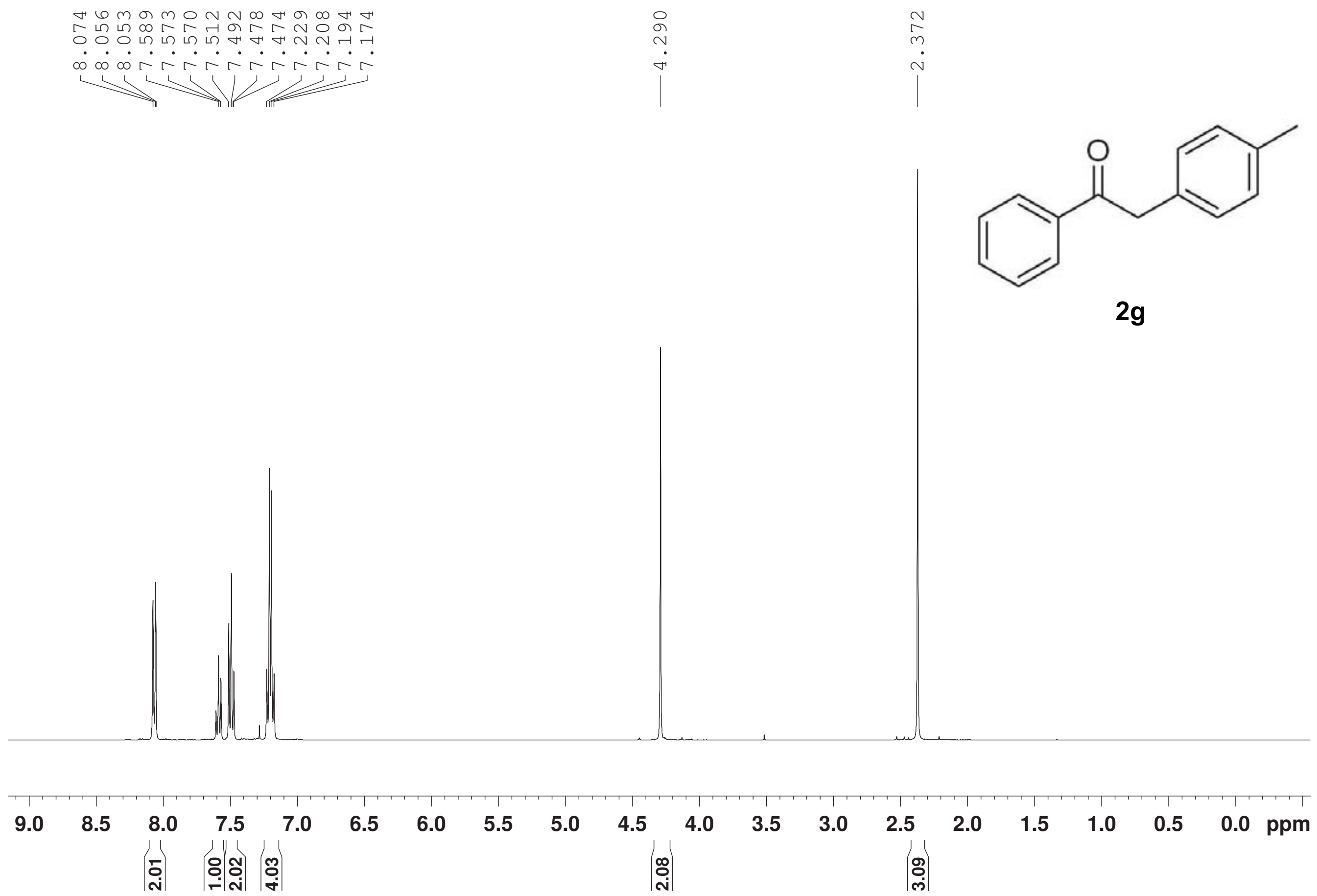
8.074
8.056
8.053
7.589
7.573
7.570
7.512
7.492
7.478
7.474
7.229
7.208
7.194
7.174
4.290
2.372
O
2g
9.0 8.5 8.0 7.5 7.0 6.5 6.0 5.5 5.0 4.5 4.0 3.5 3.0 2.5 2.0 1.5 1.0 0.5 0.0 ppm
2.01
1.00
2.02
4.03
2.08
3.09

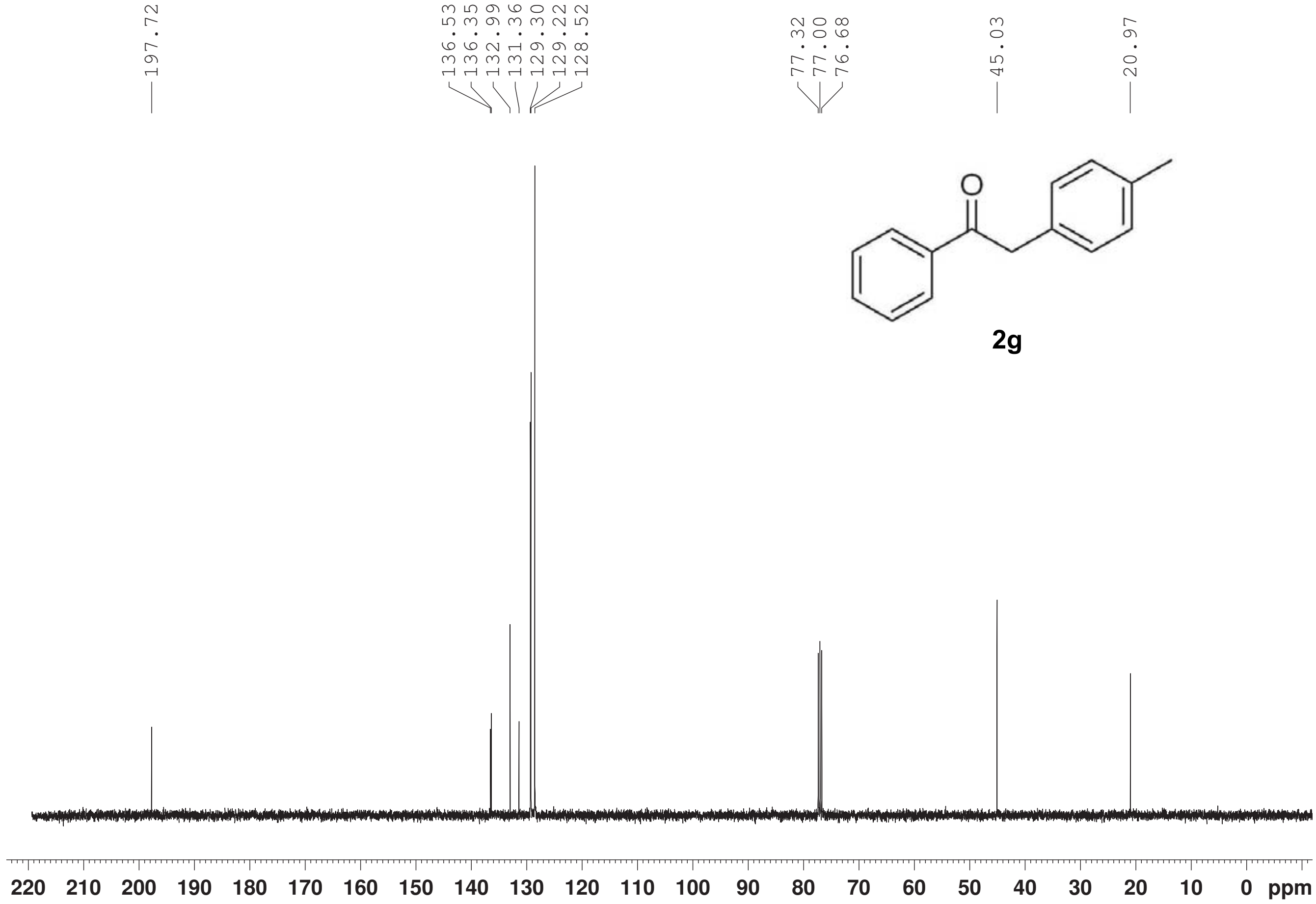
197.72
136.53
136.35
132.99
131.36
129.30
129.22
128.52
77.32
77.00
76.68
45.03
20.97
O
2g
220 210 200 190 180 170 160 150 140 130 120 110 100 90 80 70 60 50 40 30 20 10 0 ppm

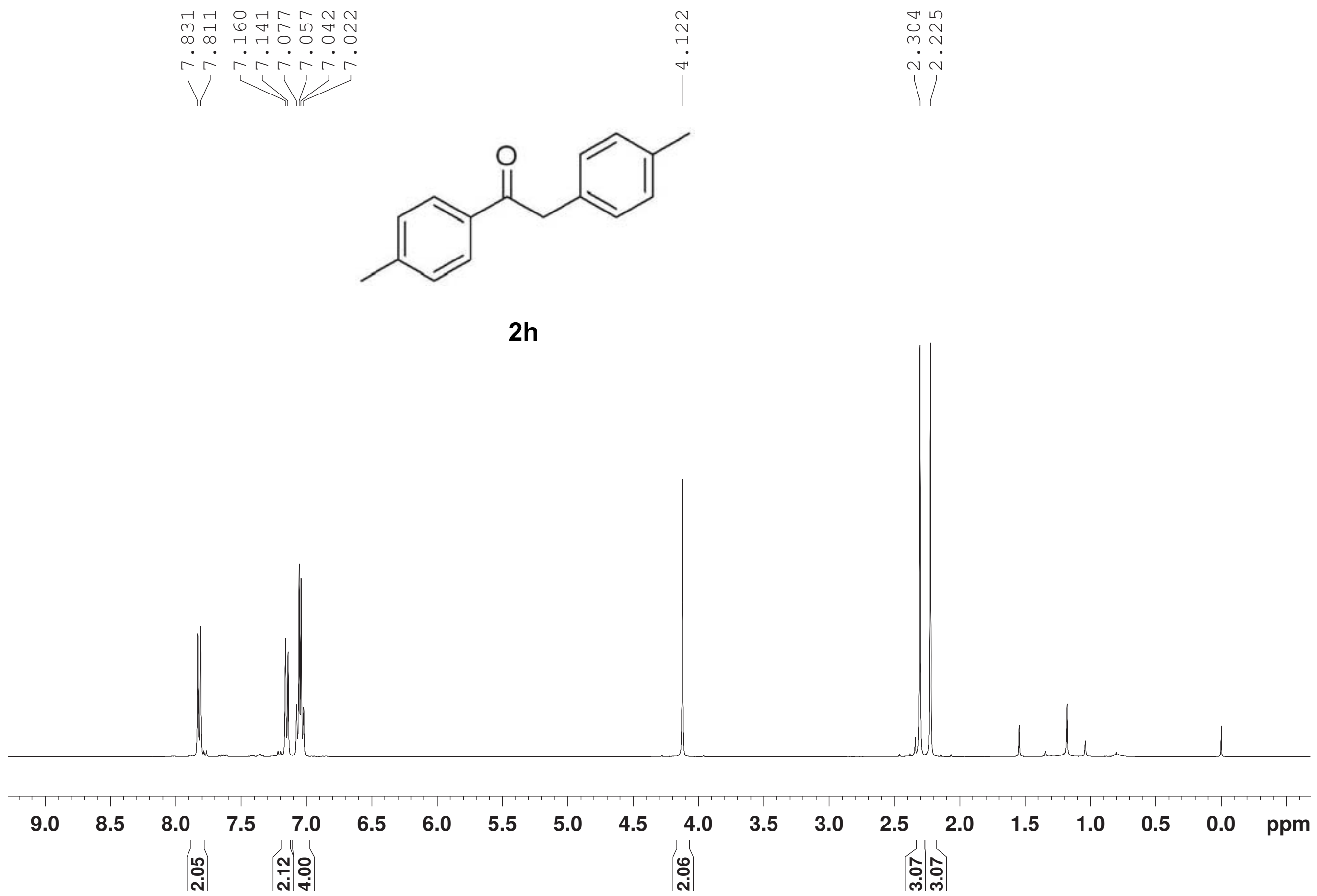

7.831
7.811
7.160
7.141
7.077
7.057
7.042
7.022
4.122
2.304
2.225
2h
9.0 8.5 8.0 7.5 7.0 6.5 6.0 5.5 5.0 4.5 4.0 3.5 3.0 2.5 2.0 1.5 1.0 0.5 0.0 ppm
2.05
2.12
4.00
2.06
3.07
3.07

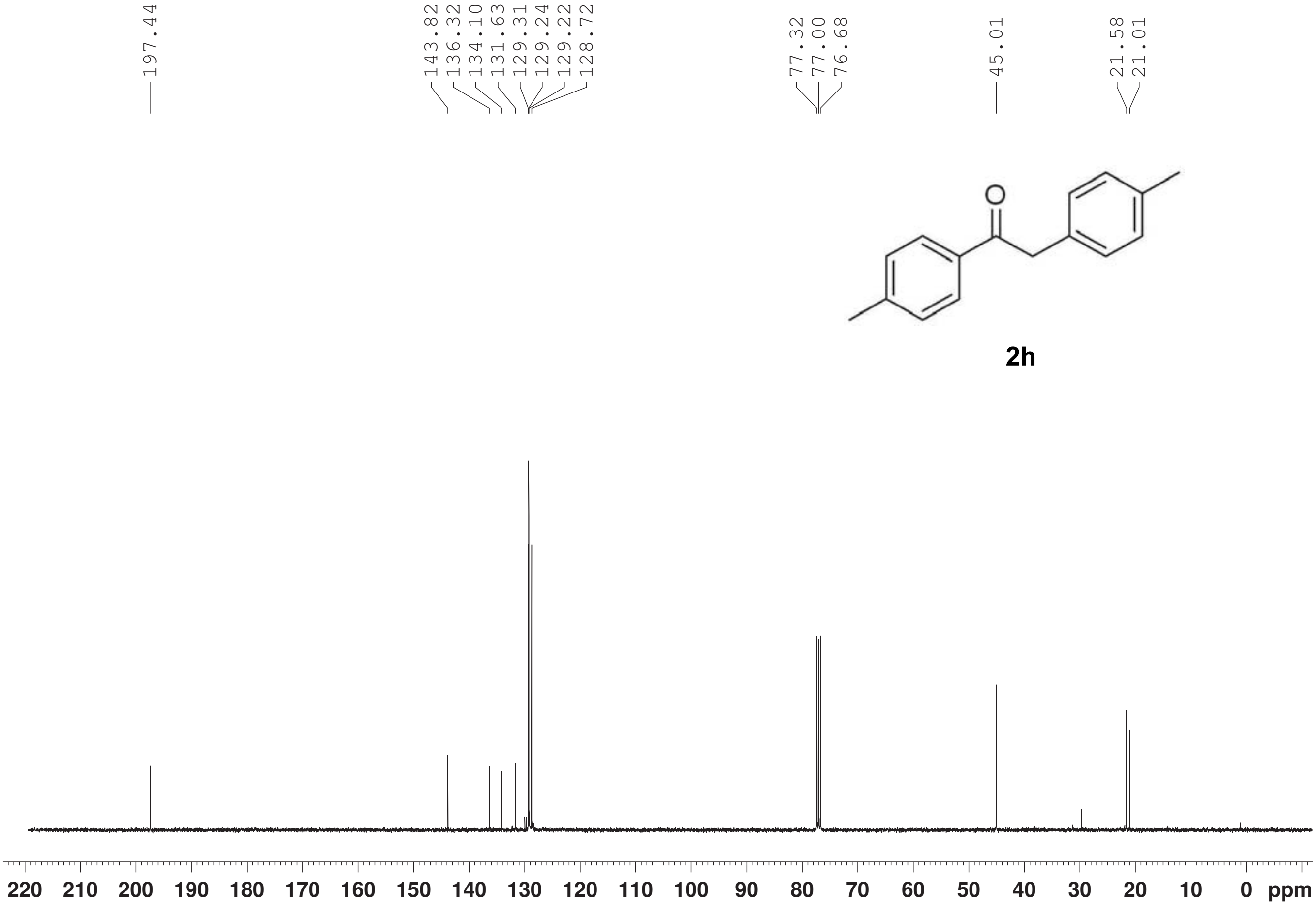

197.44
143.82
136.32
134.10
131.63
129.31
129.24
129.22
128.72
77.32
77.00
76.68
45.01
21.58
21.01
2h
220 210 200 190 180 170 160 150 140 130 120 110 100 90 80 70 60 50 40 30 20 10 0 ppm

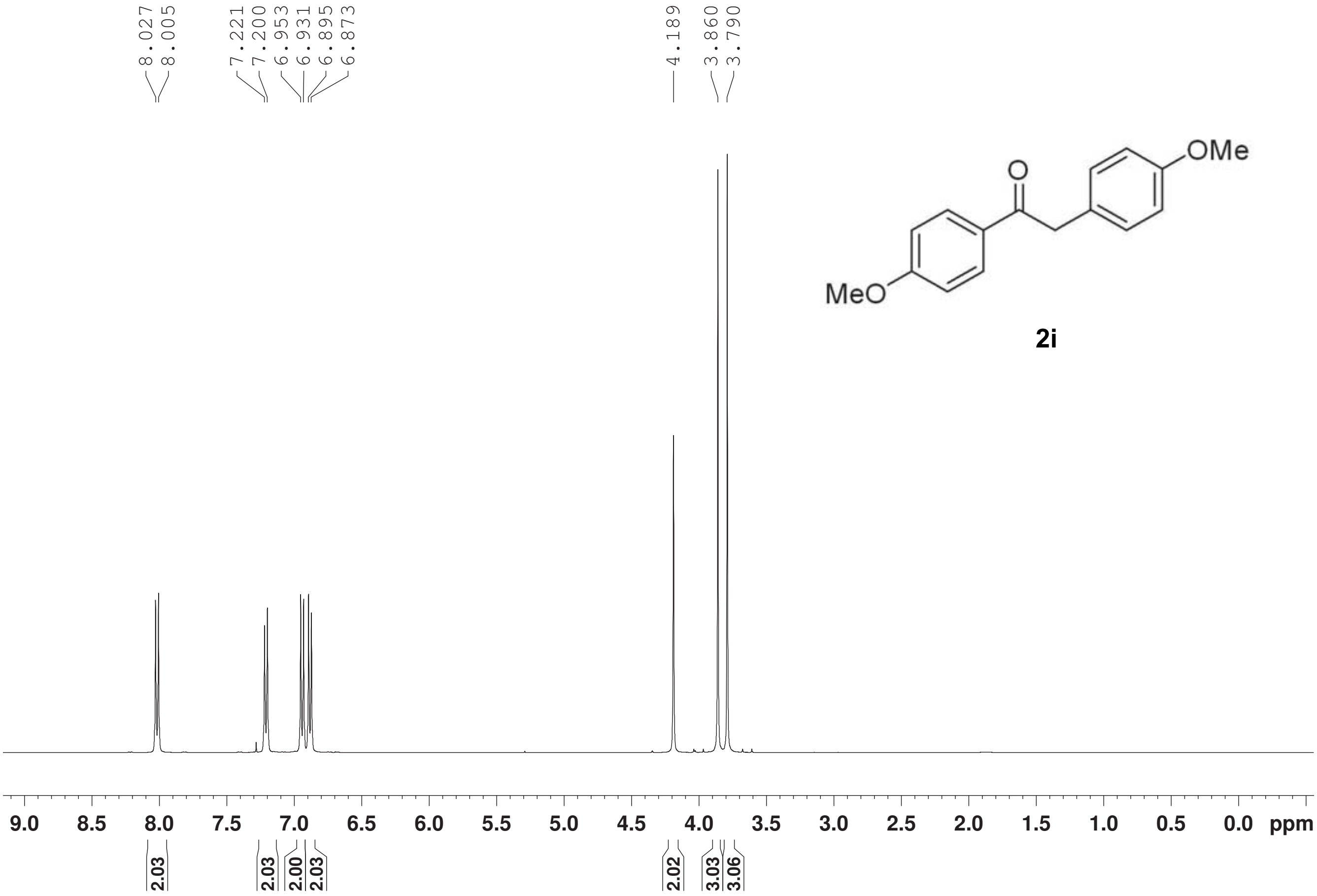
8.027
8.005
7.221
7.200
6.953
6.931
6.895
6.873
4.189
3.860
3.790
OMe
O
MeO
2i
9.0 8.5 8.0 7.5 7.0 6.5 6.0 5.5 5.0 4.5 4.0 3.5 3.0 2.5 2.0 1.5 1.0 0.5 0.0 ppm
2.03
2.03
2.00
2.03
2.02
3.03
3.06

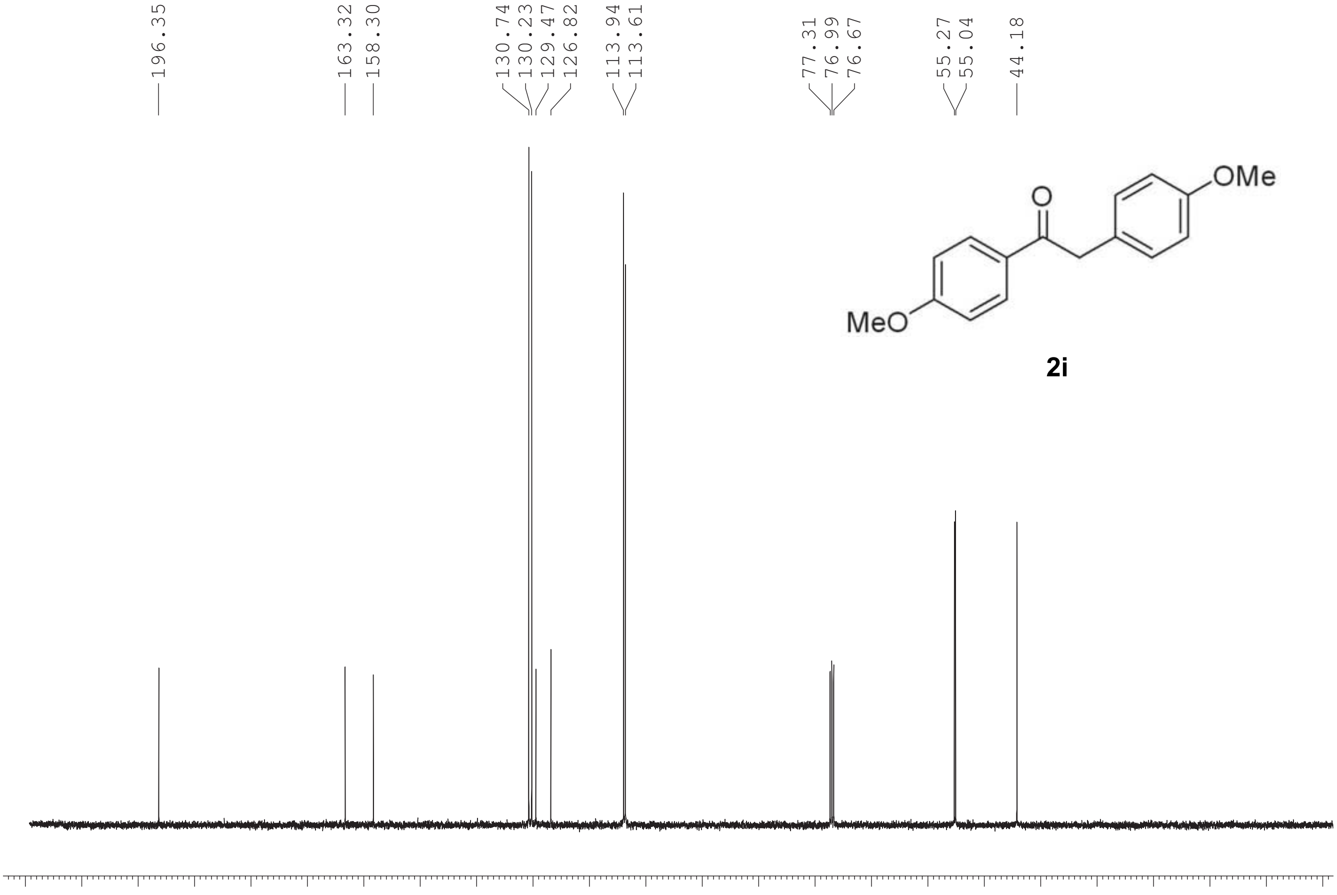
196.35
163.32
158.30
130.74
130.23
129.47
126.82
113.94
113.61
77.31
76.99
76.67
55.27
55.04
44.18
OMe
O
MeO
2i
220 210 200 190 180 170 160 150 140 130 120 110 100 90 80 70 60 50 40 30 20 10 0 ppm

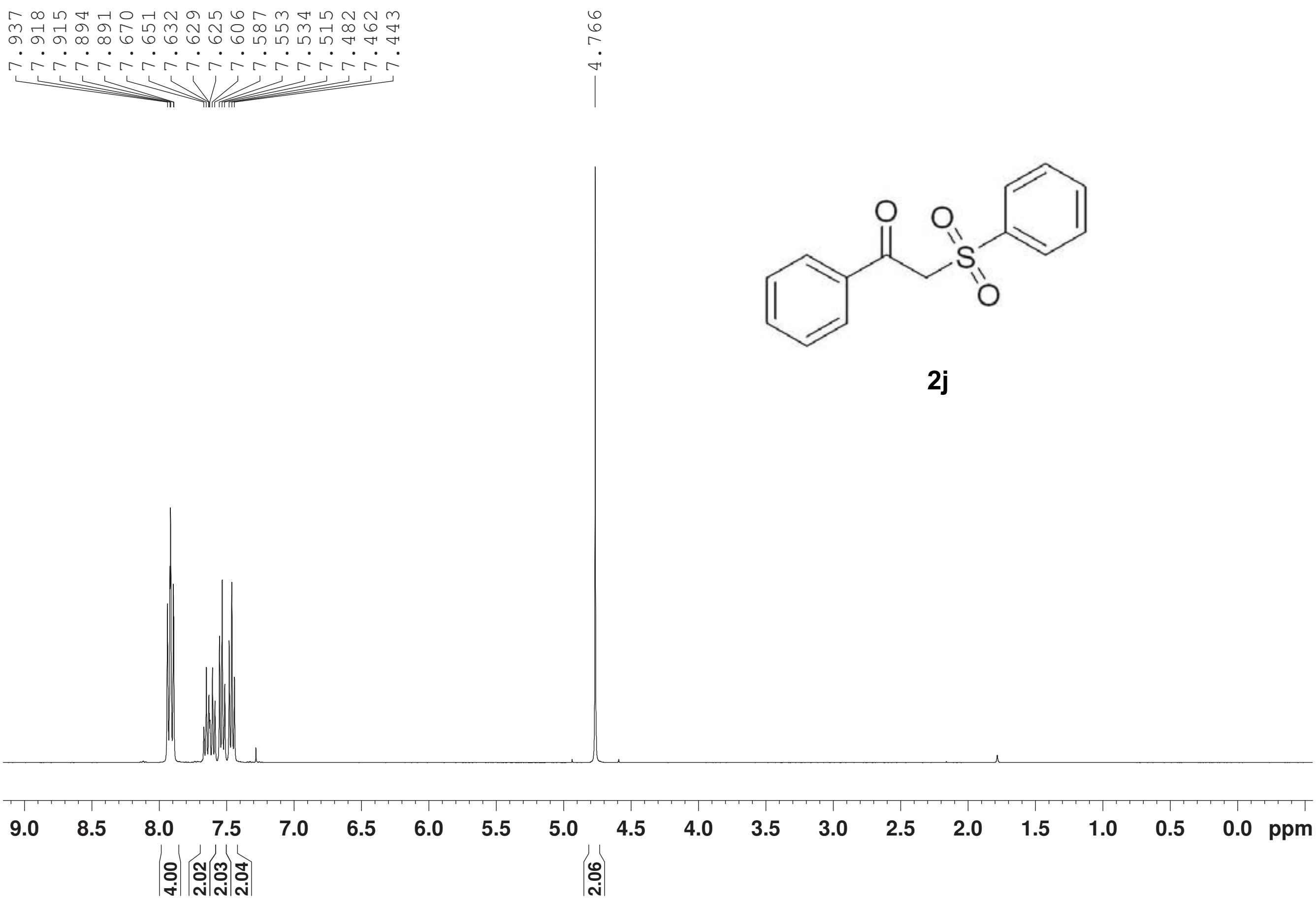
7.937
7.918
7.915
7.894
7.891
7.670
7.651
7.632
7.629
7.625
7.606
7.587
7.553
7.534
7.515
7.482
7.462
7.443
4.766
2j
9.0 8.5 8.0 7.5 7.0 6.5 6.0 5.5 5.0 4.5 4.0 3.5 3.0 2.5 2.0 1.5 1.0 0.5 0.0 ppm
4.00
2.02
2.03
2.04
2.06

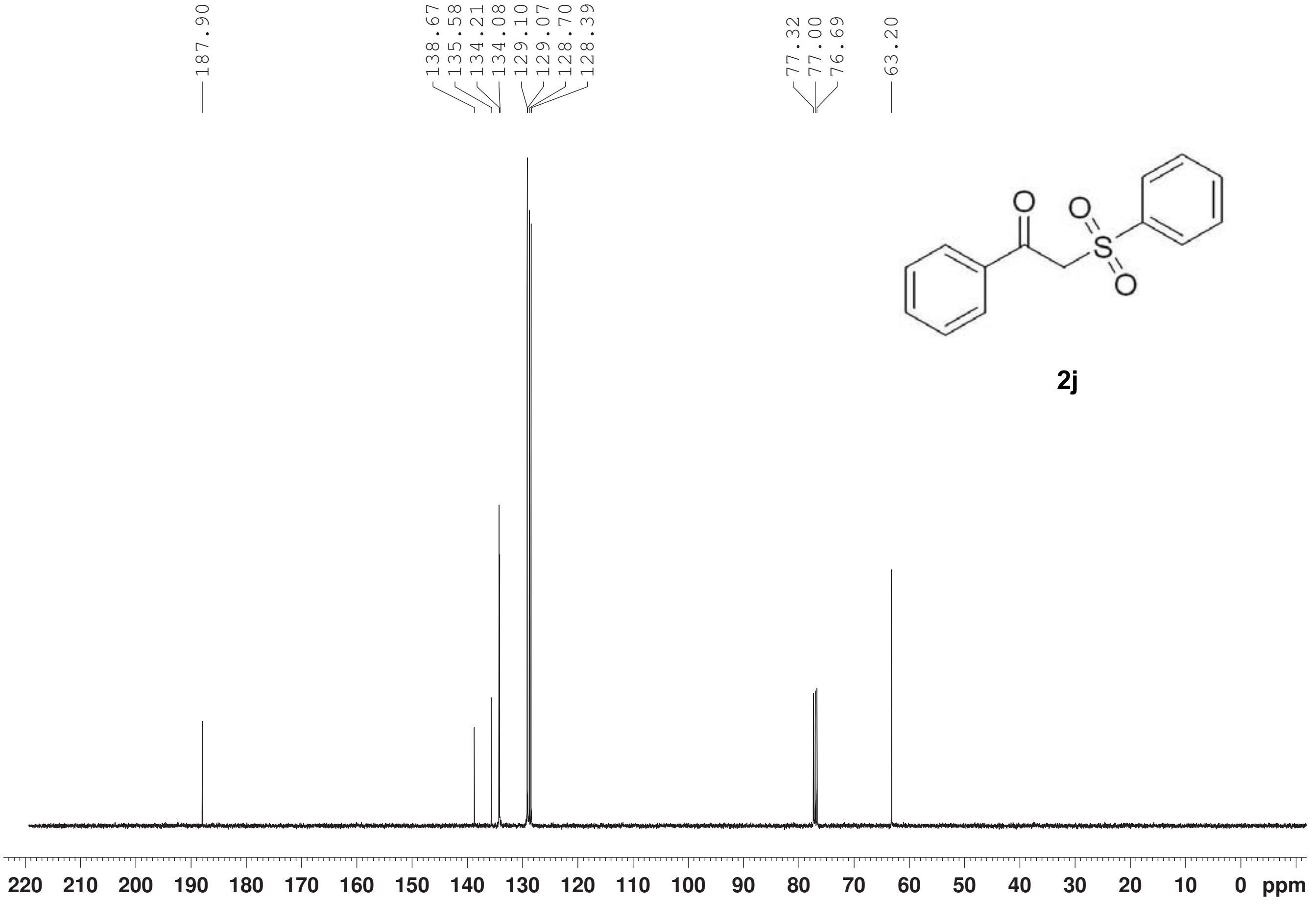
187.90
138.67
135.58
134.21
134.08
129.10
129.07
128.70
128.39
77.32
77.00
76.69
63.20
2j
220 210 200 190 180 170 160 150 140 130 120 110 100 90 80 70 60 50 40 30 20 10 0 ppm

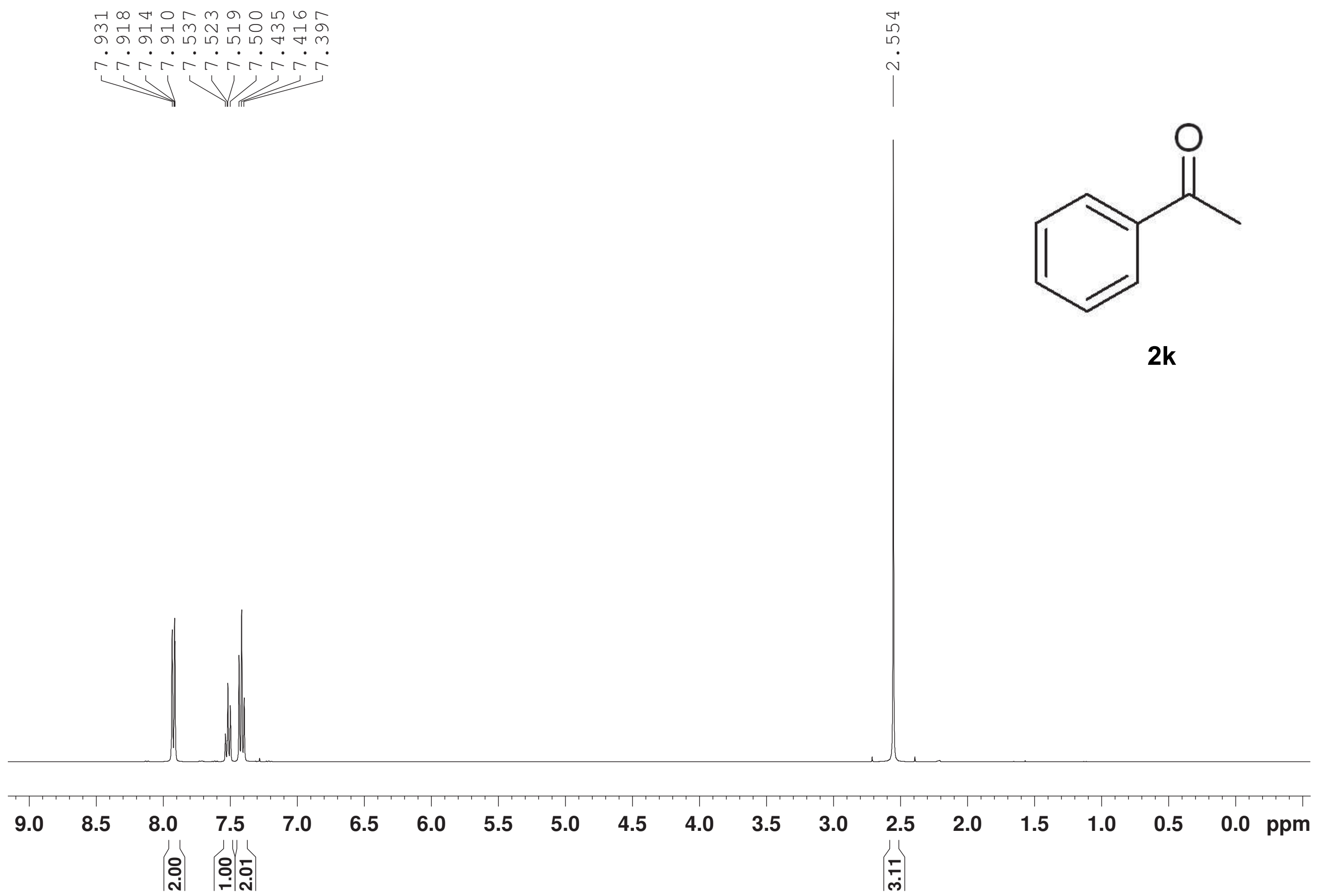
7.931
7.918
7.914
7.910
7.537
7.523
7.519
7.500
7.435
7.416
7.397
2.554
2k
9.0 8.5 8.0 7.5 7.0 6.5 6.0 5.5 5.0 4.5 4.0 3.5 3.0 2.5 2.0 1.5 1.0 0.5 0.0 ppm
2.00
1.00
2.01
3.11

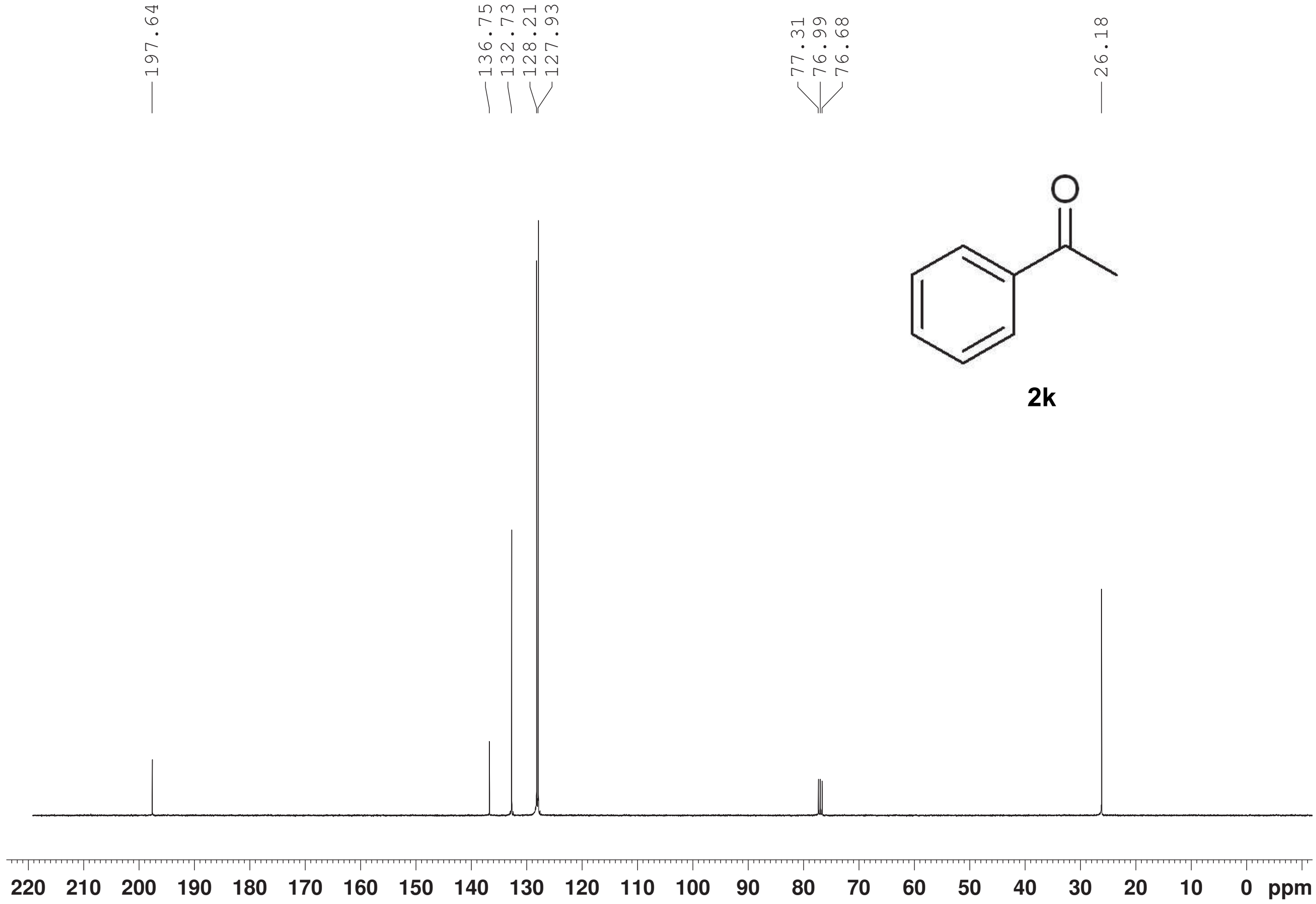
197.64
136.75
132.73
128.21
127.93
77.31
76.99
76.68
26.18
2k
220 210 200 190 180 170 160 150 140 130 120 110 100 90 80 70 60 50 40 30 20 10 0 ppm

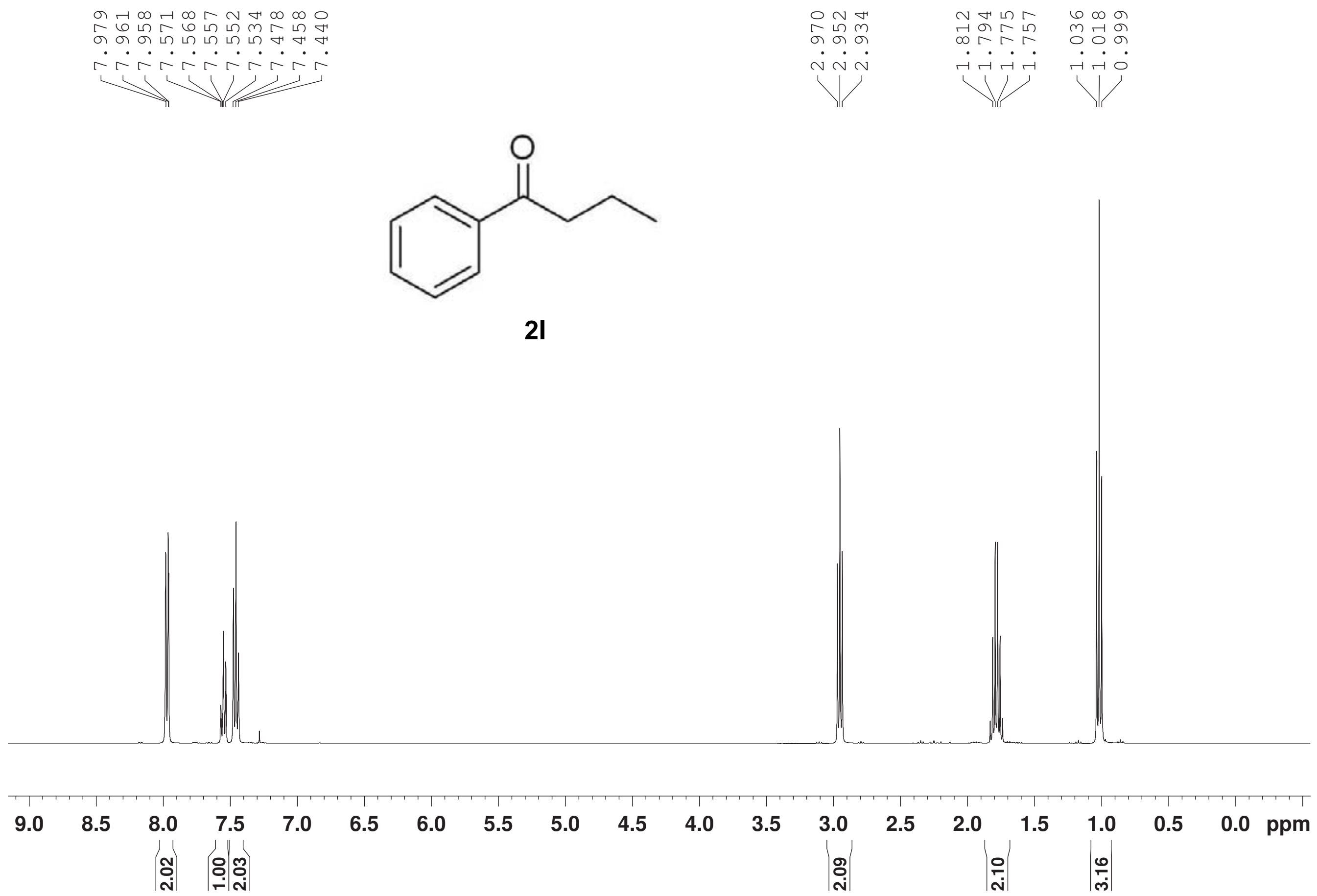
7.979
7.961
7.958
7.571
7.568
7.557
7.552
7.534
7.478
7.458
7.440
2.970
2.952
2.934
1.812
1.794
1.775
1.757
1.036
1.018
0.999
2l
9.0 8.5 8.0 7.5 7.0 6.5 6.0 5.5 5.0 4.5 4.0 3.5 3.0 2.5 2.0 1.5 1.0 0.5 0.0 ppm
2.02
1.00
2.03
2.09
2.10
3.16

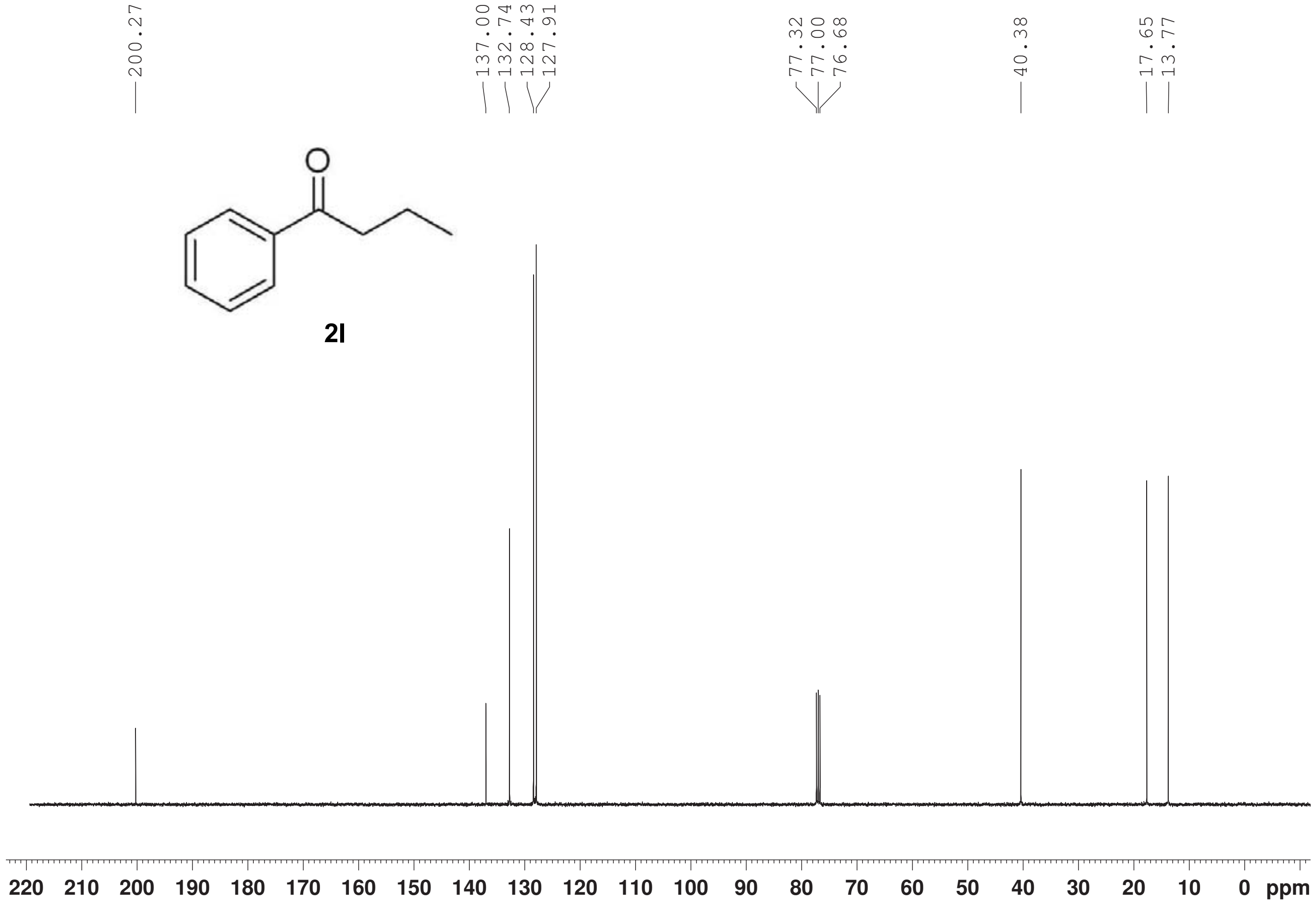
200.27
137.00
132.74
128.43
127.91
77.32
77.00
76.68
40.38
17.65
13.77
2l
220 210 200 190 180 170 160 150 140 130 120 110 100 90 80 70 60 50 40 30 20 10 0 ppm

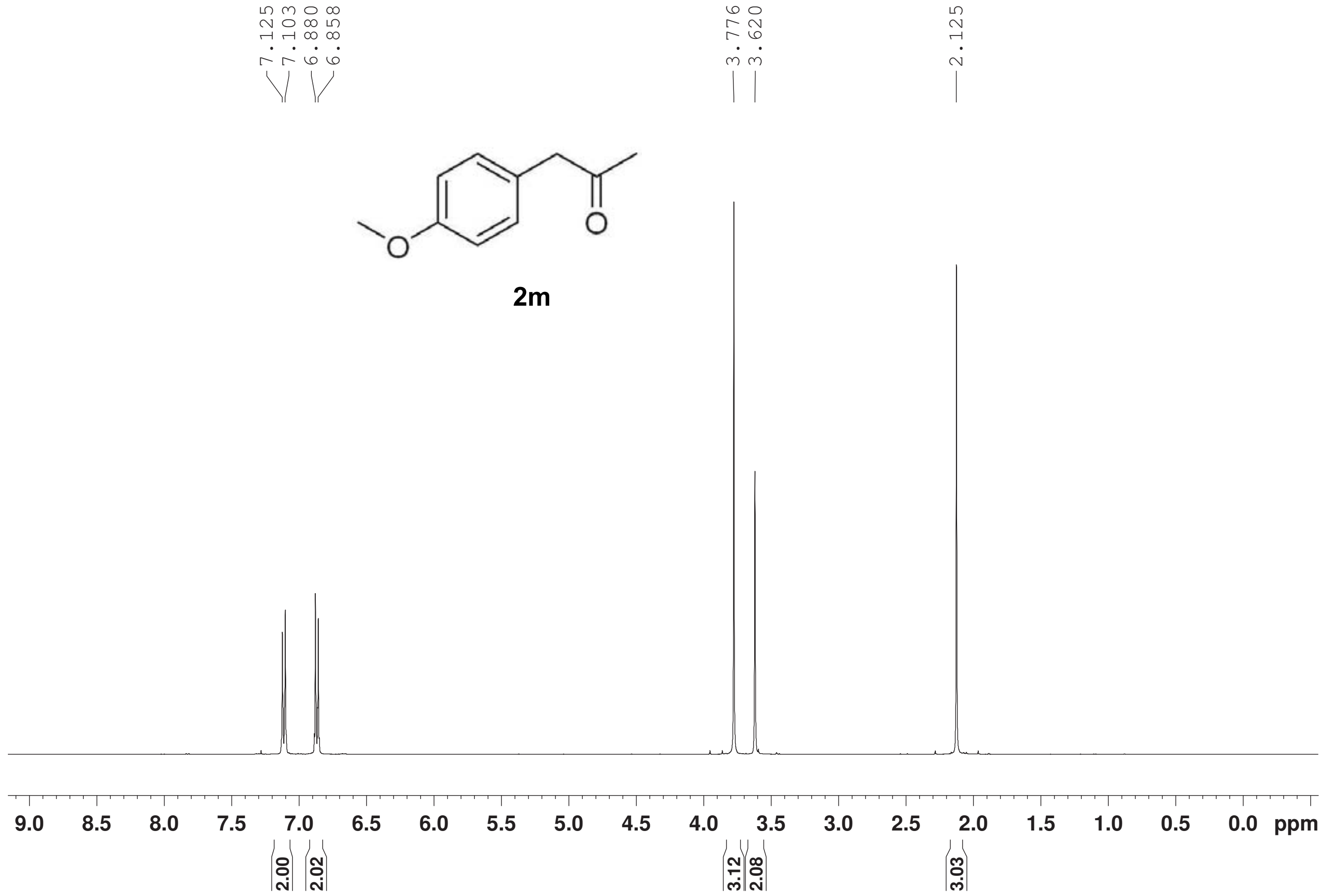
7.125
7.103
6.880
6.858
3.776
3.620
2.125
2m
9.0 8.5 8.0 7.5 7.0 6.5 6.0 5.5 5.0 4.5 4.0 3.5 3.0 2.5 2.0 1.5 1.0 0.5 0.0 ppm
2.00
2.02
3.12
2.08
3.03

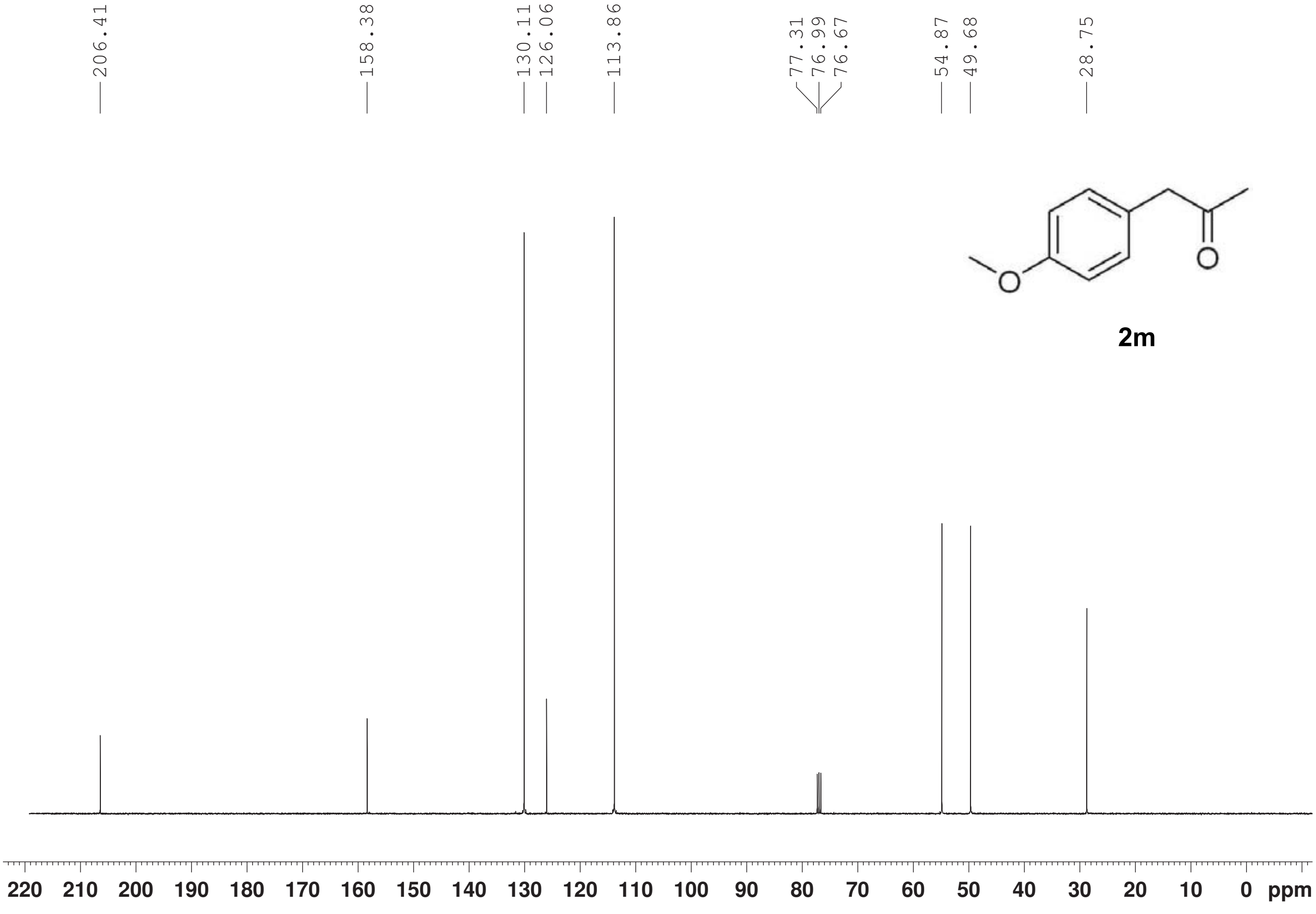
206.41
158.38
130.11
126.06
113.86
77.31
76.99
76.67
54.87
49.68
28.75
2m
220 210 200 190 180 170 160 150 140 130 120 110 100 90 80 70 60 50 40 30 20 10 0 ppm

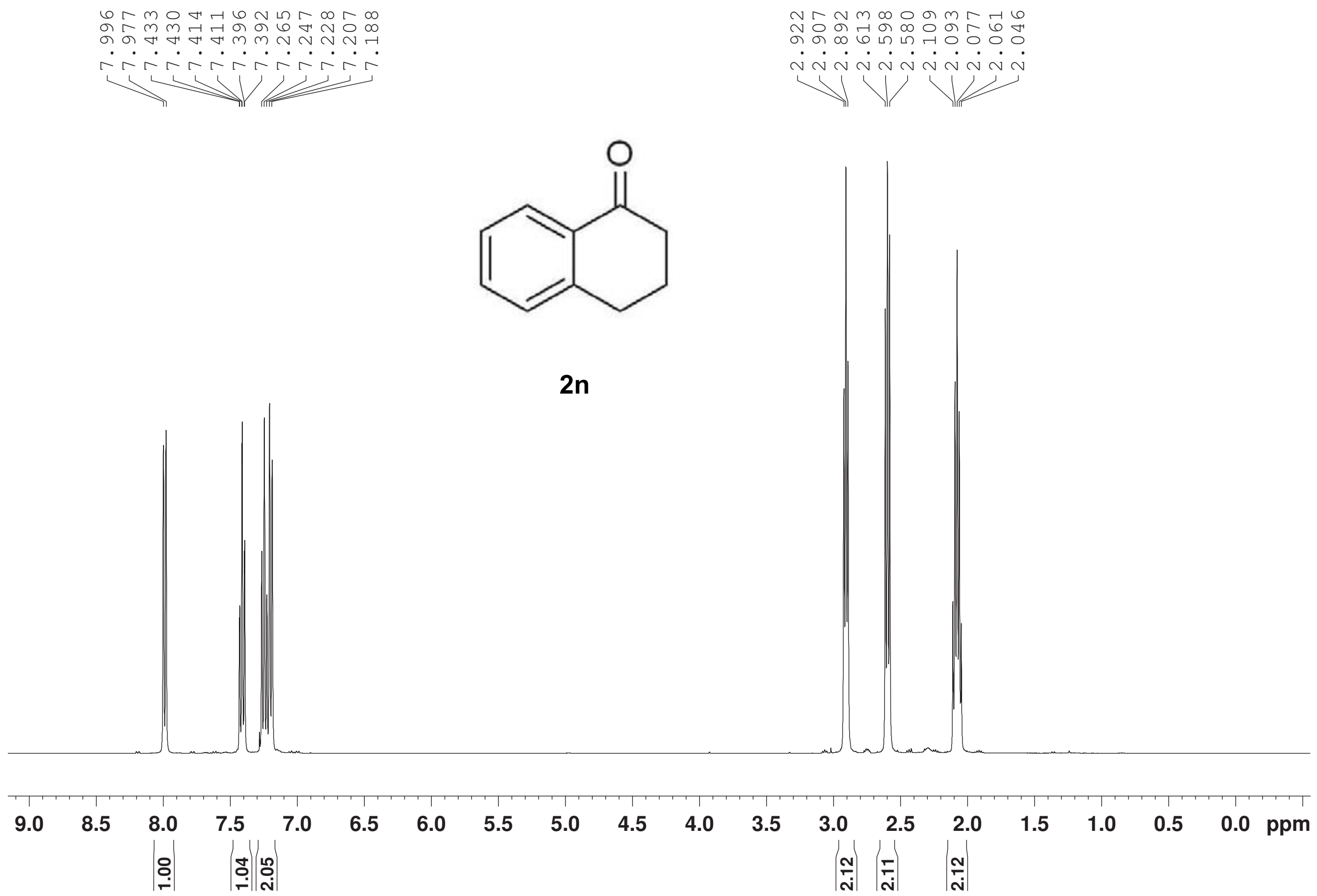
7.996
7.977
7.433
7.430
7.414
7.411
7.396
7.392
7.265
7.247
7.228
7.207
7.188
2.922
2.907
2.892
2.613
2.598
2.580
2.109
2.093
2.077
2.061
2.046
2n
9.0 8.5 8.0 7.5 7.0 6.5 6.0 5.5 5.0 4.5 4.0 3.5 3.0 2.5 2.0 1.5 1.0 0.5 0.0 ppm
1.00
1.04
2.05
2.12
2.11
2.12

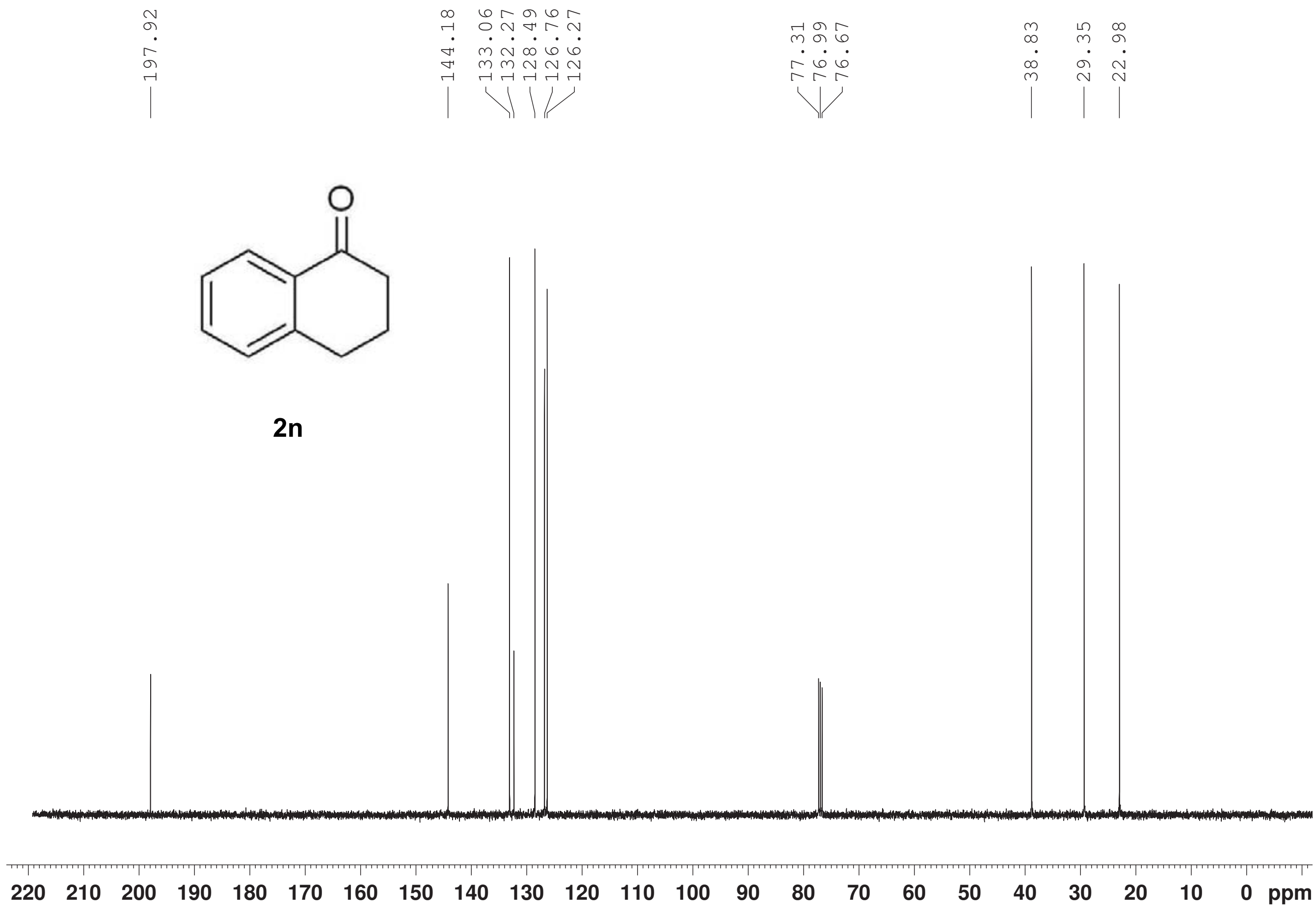
197.92
144.18
133.06
132.27
128.49
126.76
126.27
77.31
76.99
76.67
38.83
29.35
22.98
2n
220 210 200 190 180 170 160 150 140 130 120 110 100 90 80 70 60 50 40 30 20 10 0 ppm

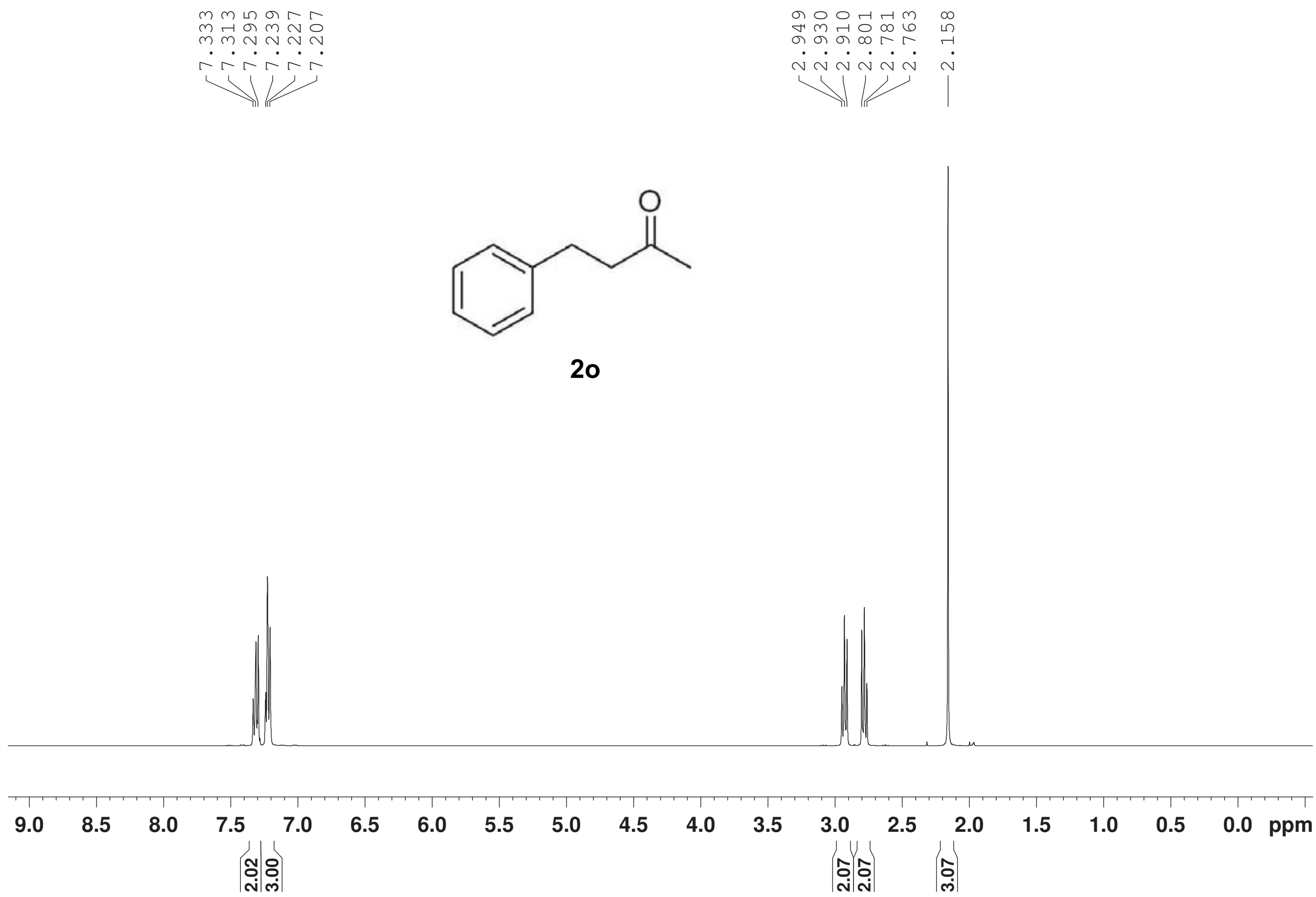
7.333
7.313
7.295
7.239
7.227
7.207
2.949
2.930
2.910
2.801
2.781
2.763
2.158
2o
9.0 8.5 8.0 7.5 7.0 6.5 6.0 5.5 5.0 4.5 4.0 3.5 3.0 2.5 2.0 1.5 1.0 0.5 0.0 ppm
2.02
3.00
2.07
2.07
3.07

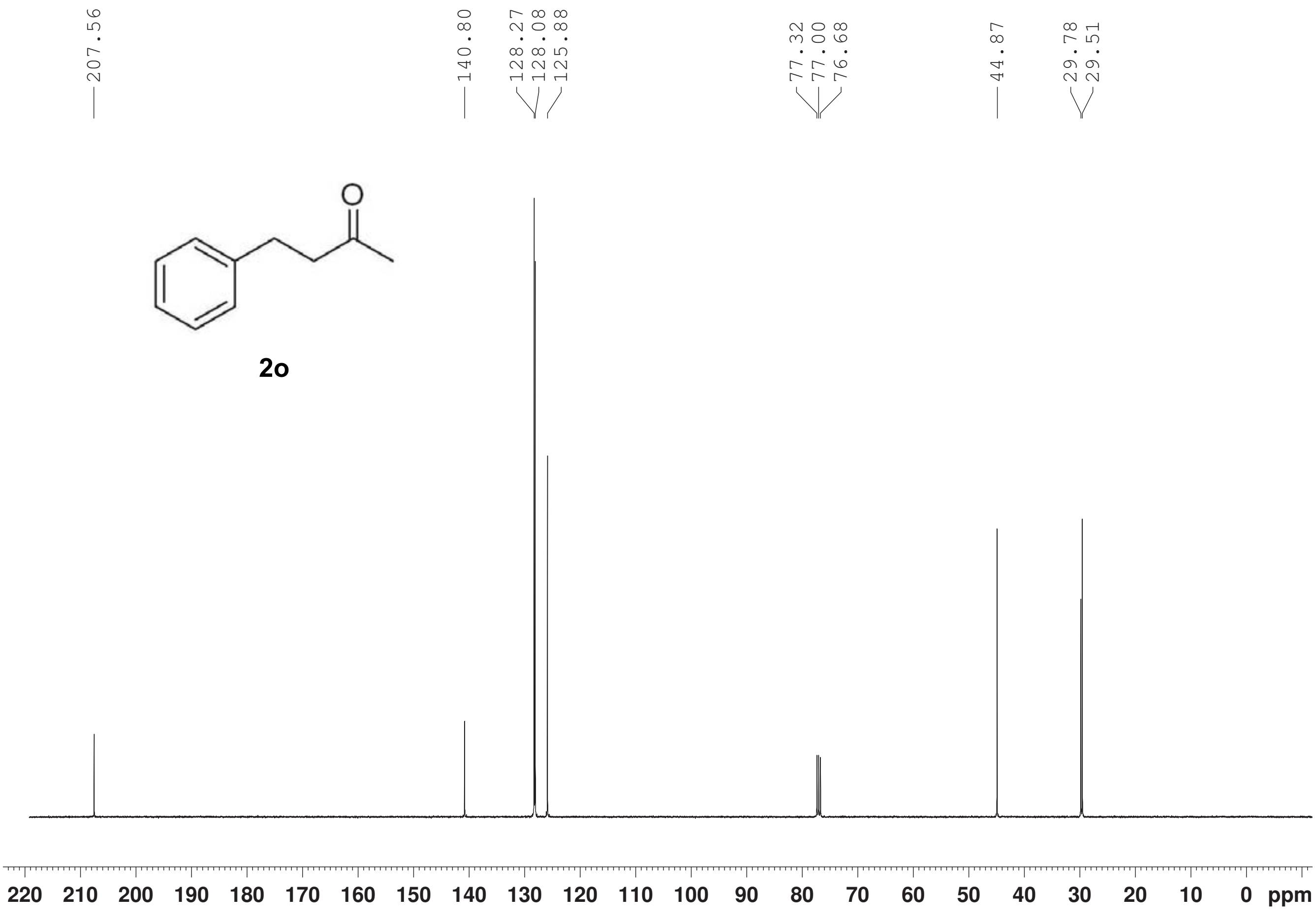
207.56
140.80
128.27
128.08
125.88
77.32
77.00
76.68
44.87
29.78
29.51
2o
220 210 200 190 180 170 160 150 140 130 120 110 100 90 80 70 60 50 40 30 20 10 0 ppm

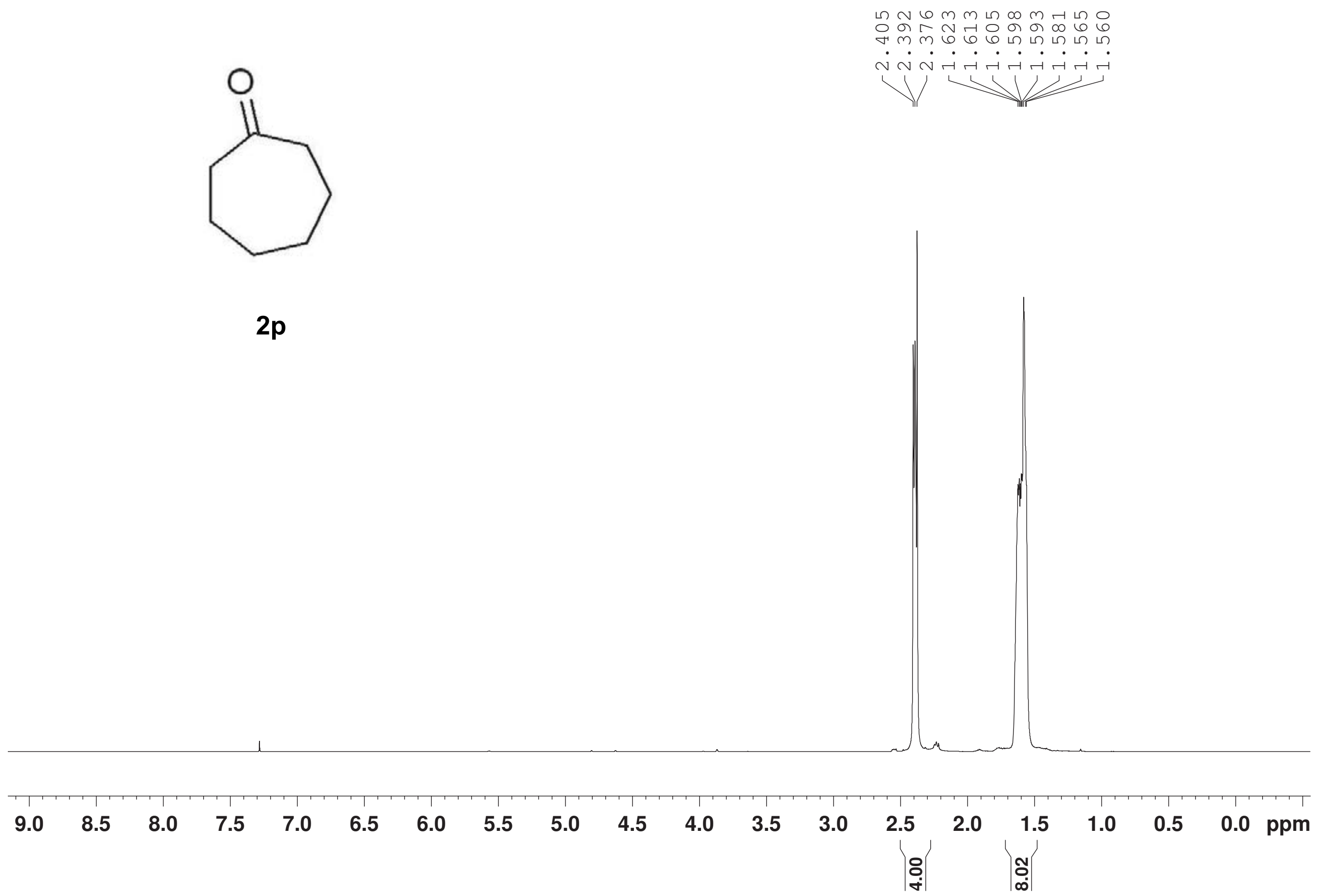
2.405
2.392
2.376
1.623
1.613
1.605
1.598
1.593
1.581
1.565
1.560
9.0 8.5 8.0 7.5 7.0 6.5 6.0 5.5 5.0 4.5 4.0 3.5 3.0 2.5 2.0 1.5 1.0 0.5 0.0 ppm
4.00
8.02

2p

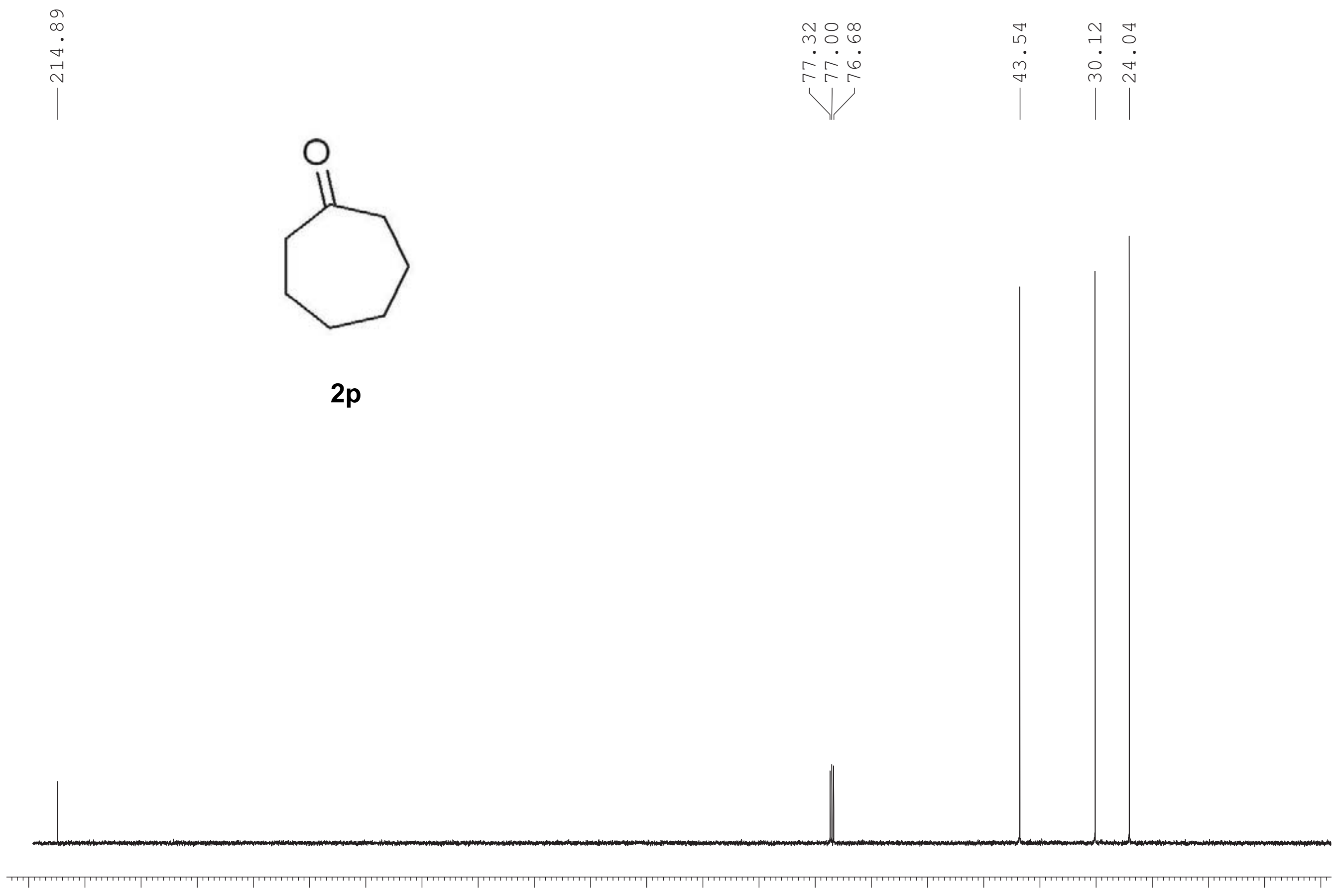
214.89
77.32
77.00
76.68
43.54
30.12
24.04
220 210 200 190 180 170 160 150 140 130 120 110 100 90 80 70 60 50 40 30 20 10 0 ppm

2p

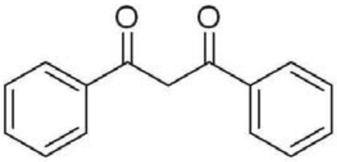

2q

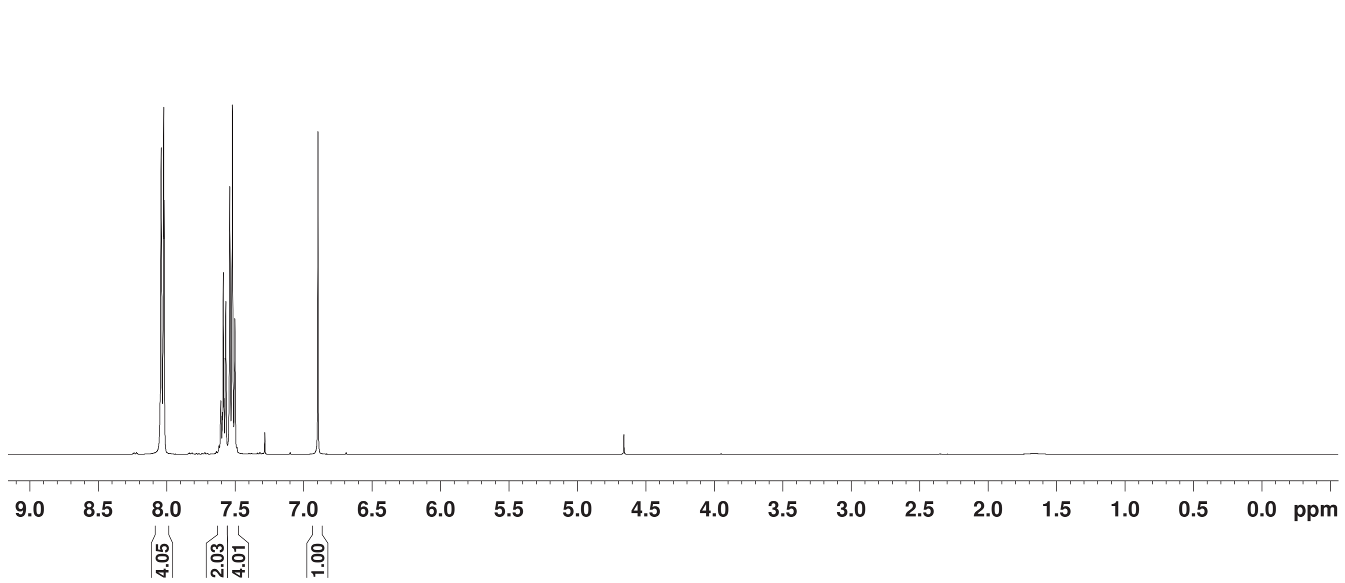

8.038
8.025
8.020
8.017
7.606
7.587
7.582
7.572
7.569
7.566
7.540
7.524
7.521
7.507
7.503
6.896
9.0 8.5 8.0 7.5 7.0 6.5 6.0 5.5 5.0 4.5 4.0 3.5 3.0 2.5 2.0 1.5 1.0 0.5 0.0 ppm
4.05
2.03
4.01
1.00

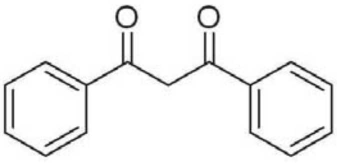

2q

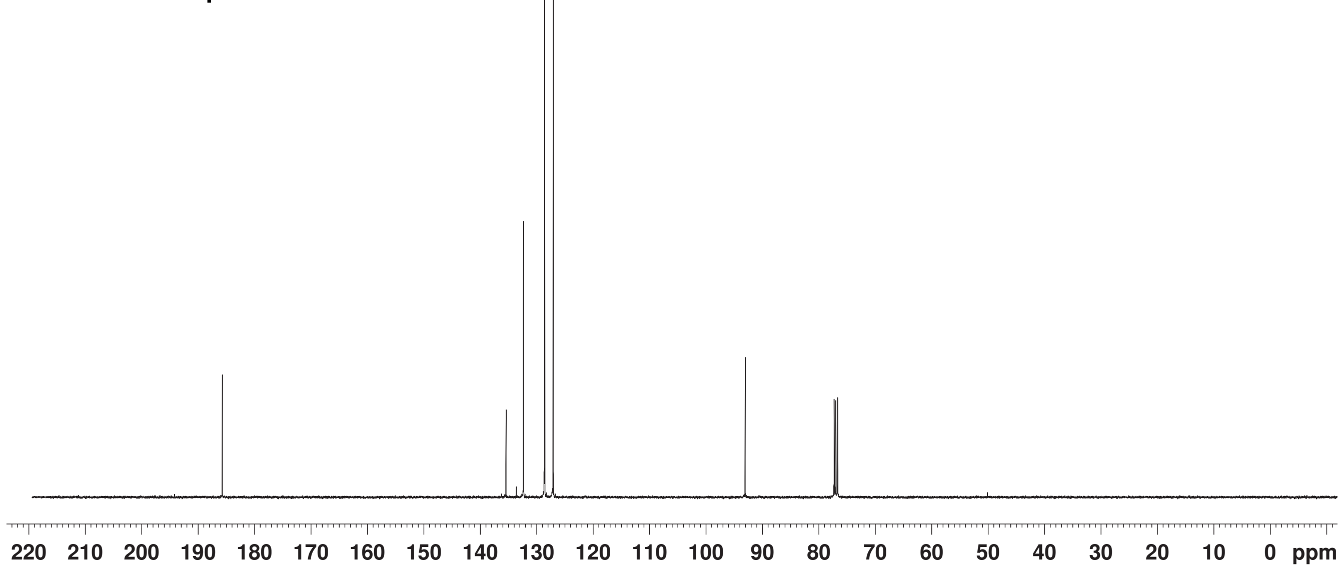

185.68
135.47
132.38
128.61
127.10
93.08
77.31
76.99
76.67
220 210 200 190 180 170 160 150 140 130 120 110 100 90 80 70 60 50 40 30 20 10 0 ppm

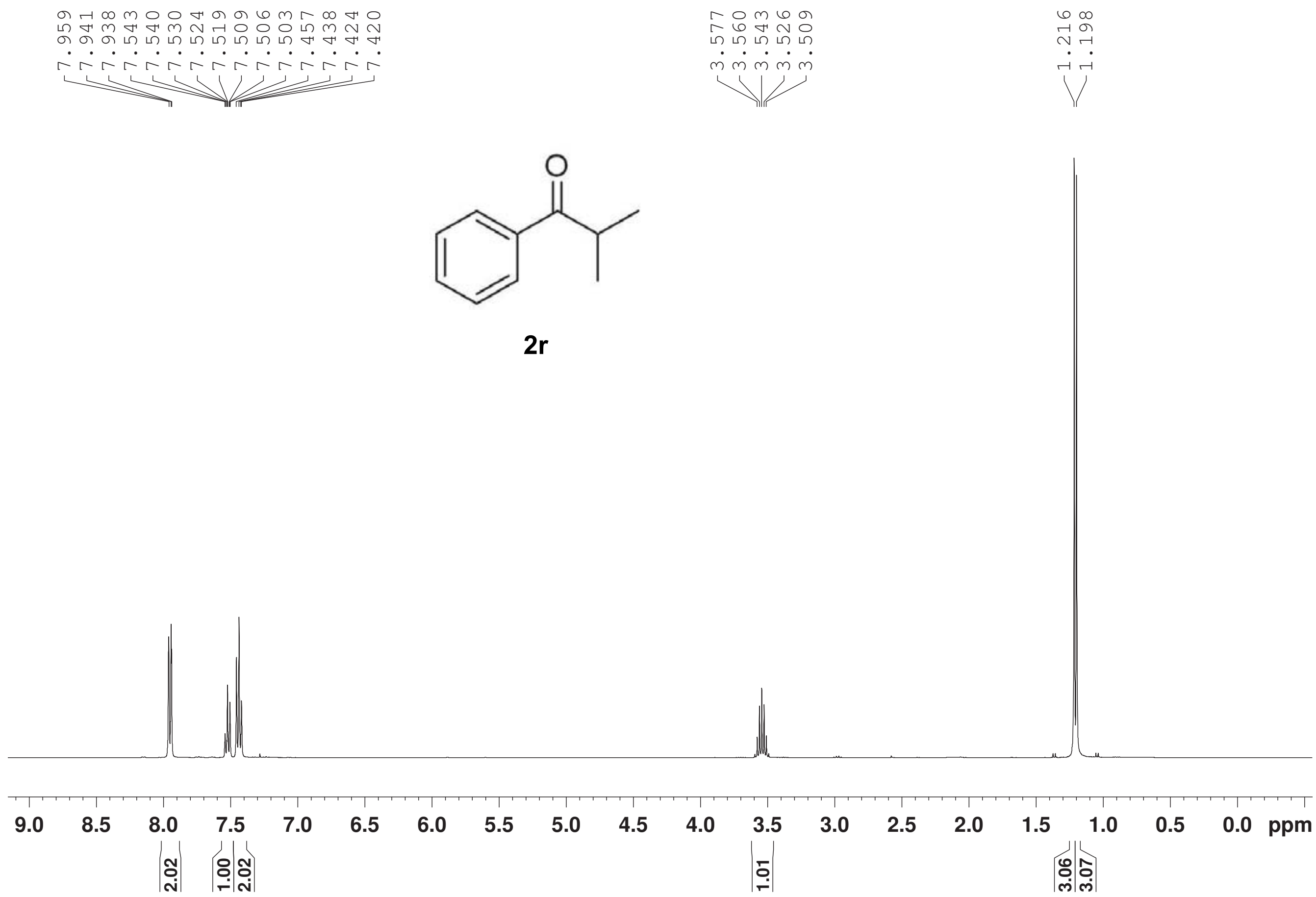
7.959
7.941
7.938
7.543
7.540
7.530
7.524
7.519
7.509
7.506
7.503
7.457
7.438
7.424
7.420
3.577
3.560
3.543
3.526
3.509
1.216
1.198
2r
9.0 8.5 8.0 7.5 7.0 6.5 6.0 5.5 5.0 4.5 4.0 3.5 3.0 2.5 2.0 1.5 1.0 0.5 0.0 ppm
2.02
1.00
2.02
1.01
3.06
3.07

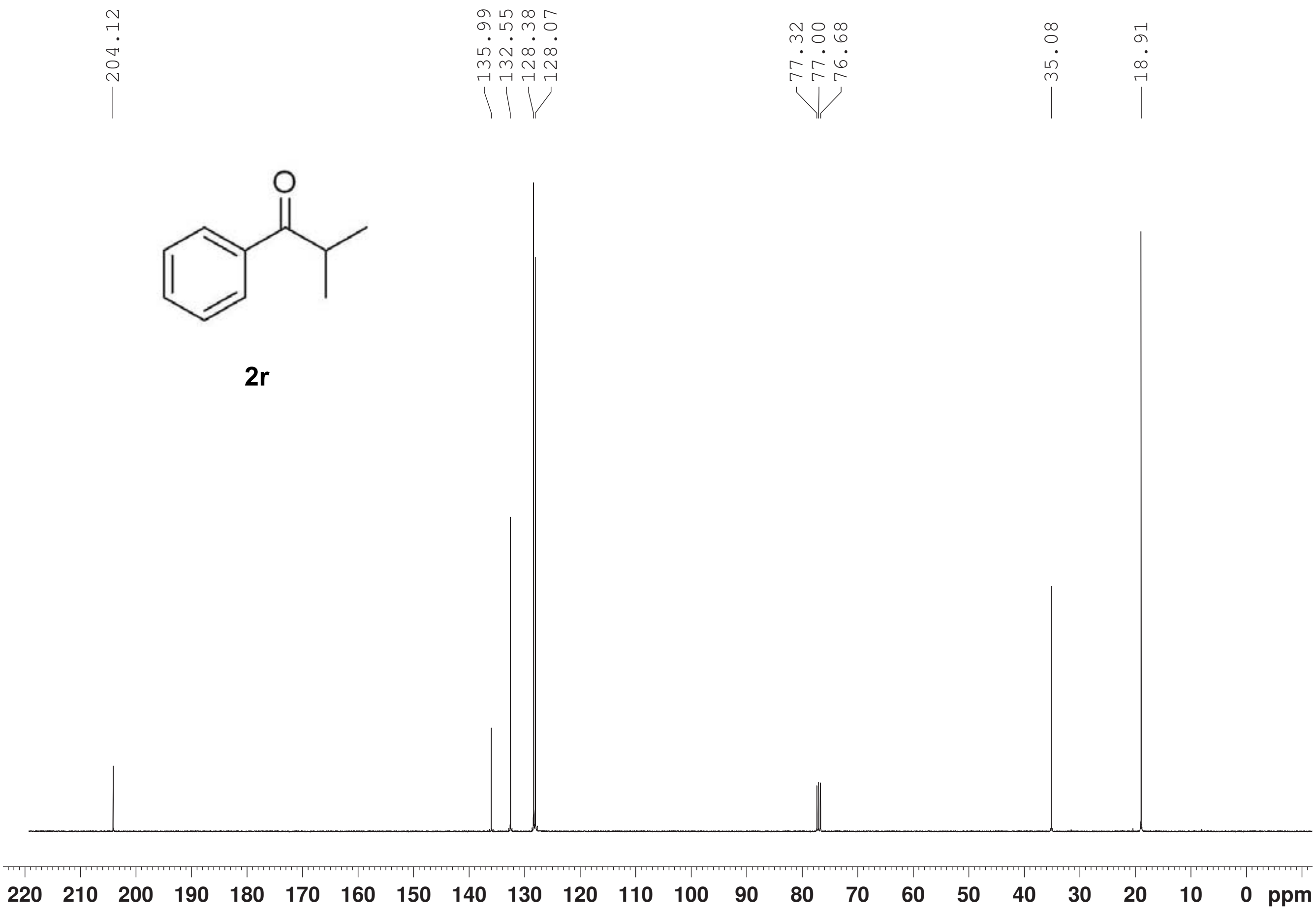
204.12
135.99
132.55
128.38
128.07
77.32
77.00
76.68
35.08
18.91
2r
220 210 200 190 180 170 160 150 140 130 120 110 100 90 80 70 60 50 40 30 20 10 0 ppm

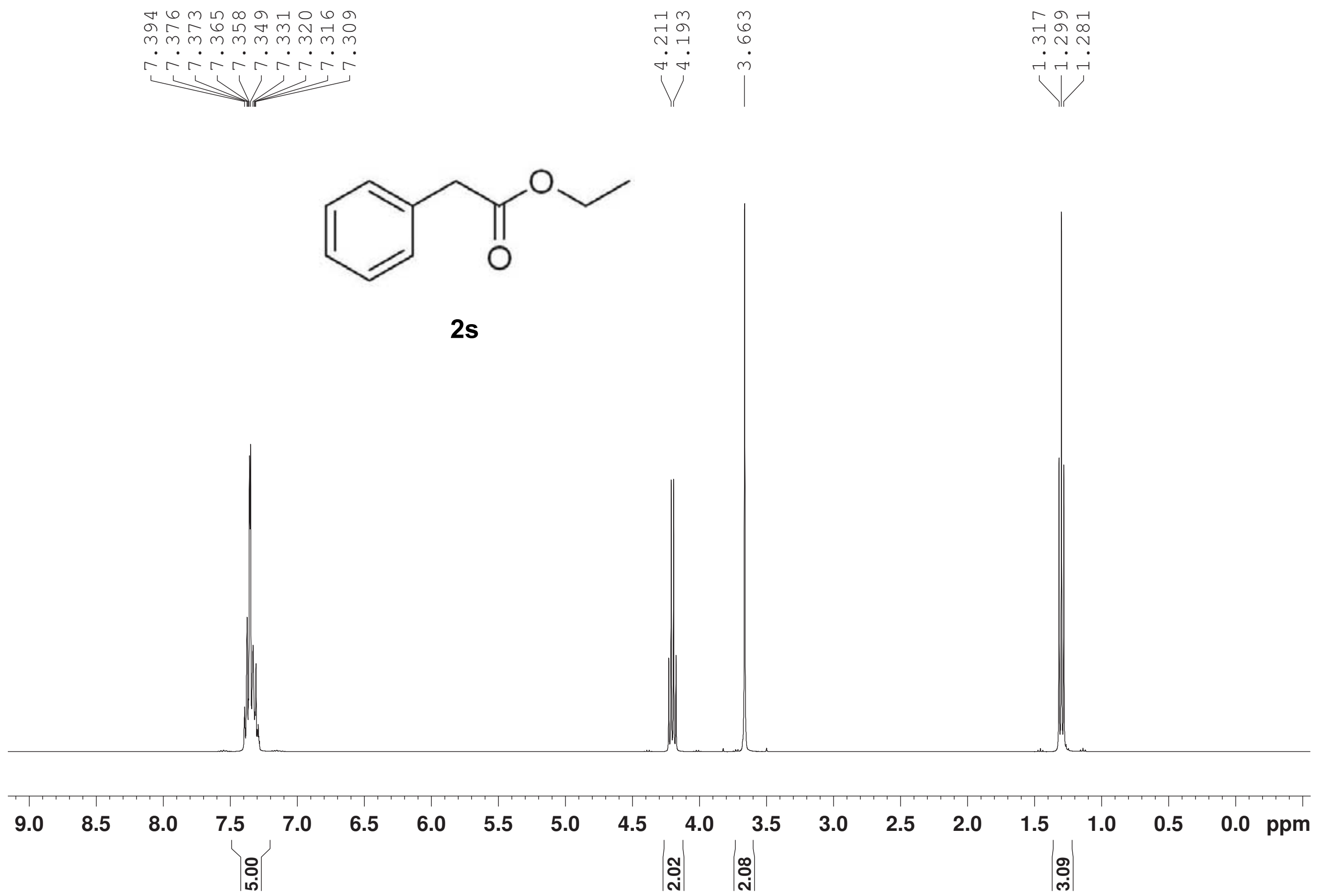
7.394
7.376
7.373
7.365
7.358
7.349
7.331
7.320
7.316
7.309
4.211
4.193
3.663
1.317
1.299
1.281
2s
9.0 8.5 8.0 7.5 7.0 6.5 6.0 5.5 5.0 4.5 4.0 3.5 3.0 2.5 2.0 1.5 1.0 0.5 0.0 ppm
5.00
2.02
2.08
3.09

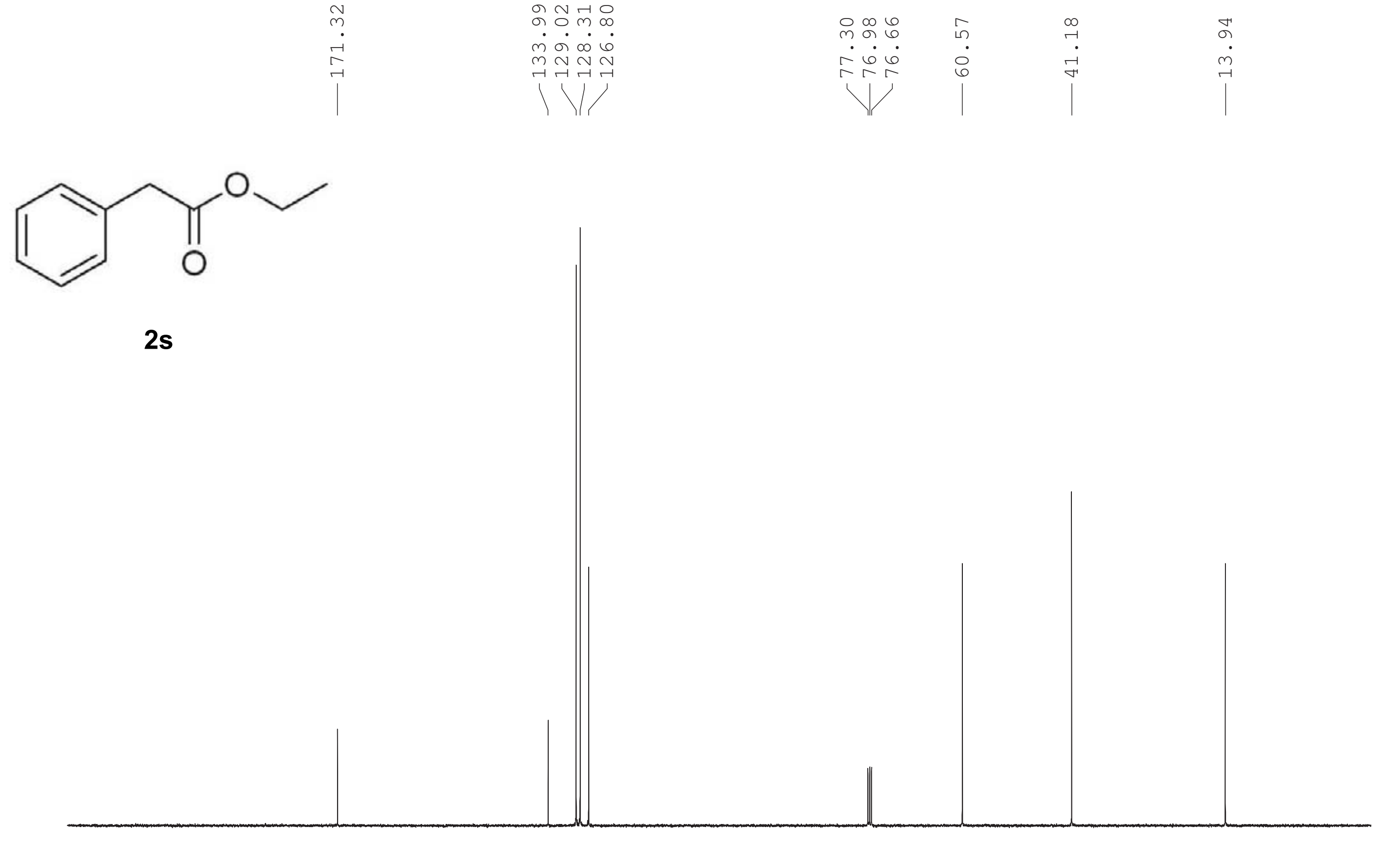
171.32
133.99
129.02
128.31
126.80
77.30
76.98
76.66
60.57
41.18
13.94
2s
220 210 200 190 180 170 160 150 140 130 120 110 100 90 80 70 60 50 40 30 20 10 0 ppm

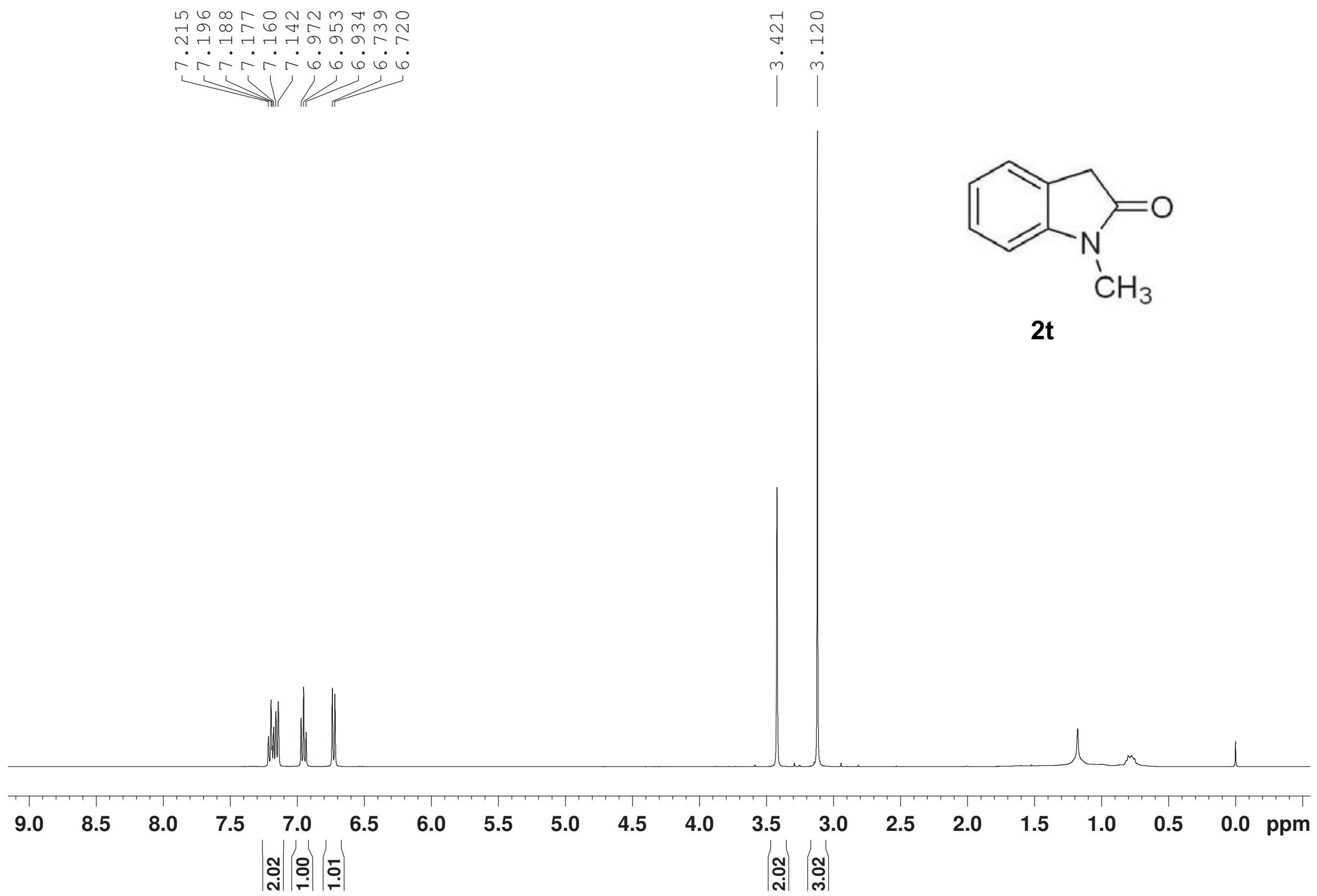
7.215
7.196
7.188
7.177
7.160
7.142
6.972
6.953
6.934
6.739
6.720
3.421
3.120
N
O
CH3
2t
9.0 8.5 8.0 7.5 7.0 6.5 6.0 5.5 5.0 4.5 4.0 3.5 3.0 2.5 2.0 1.5 1.0 0.5 0.0 ppm
2.02
1.00
1.01
2.02
3.02

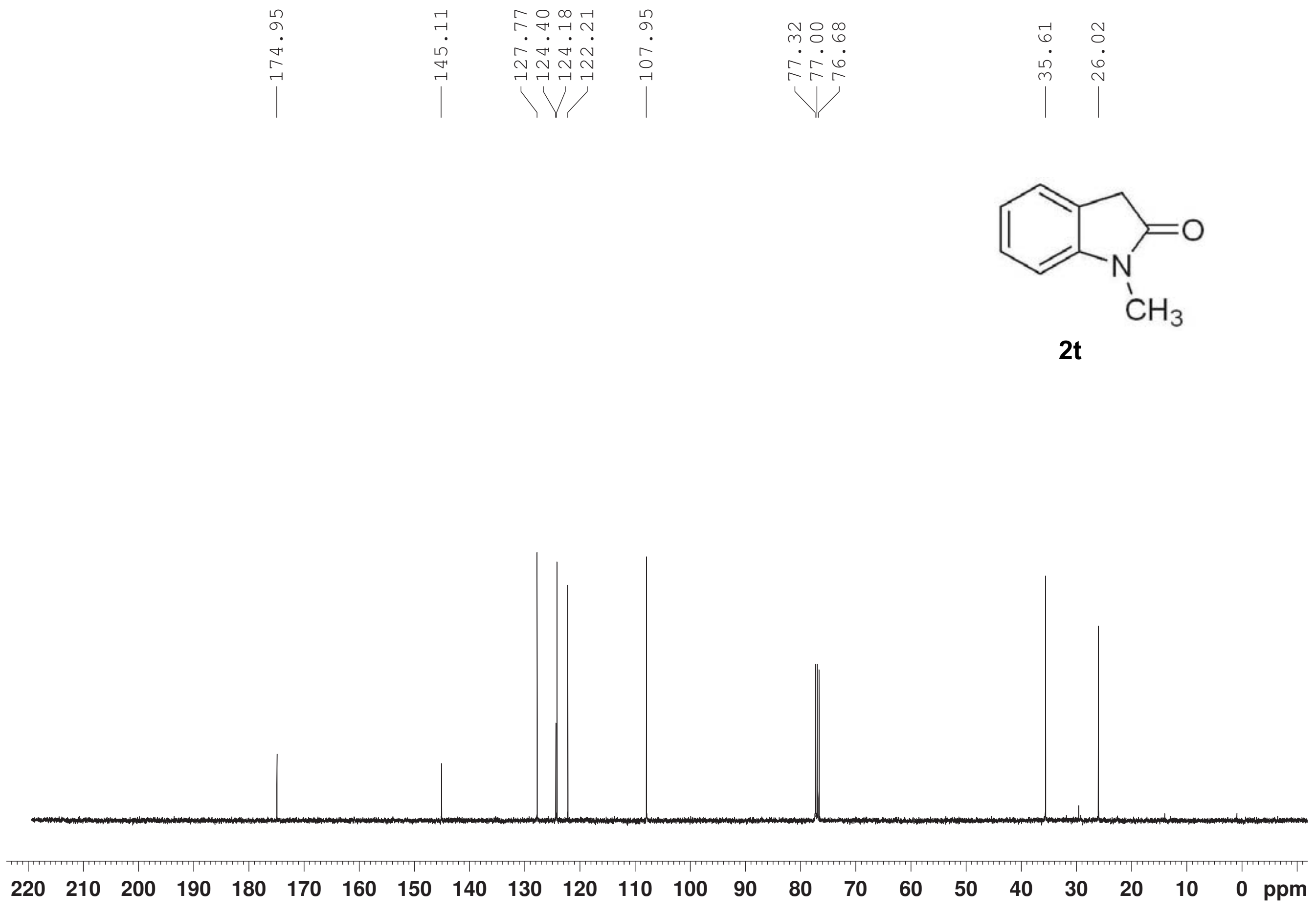
174.95
145.11
127.77
124.40
124.18
122.21
107.95
77.32
77.00
76.68
35.61
26.02
N
O
CH3
2t
220 210 200 190 180 170 160 150 140 130 120 110 100 90 80 70 60 50 40 30 20 10 0 ppm

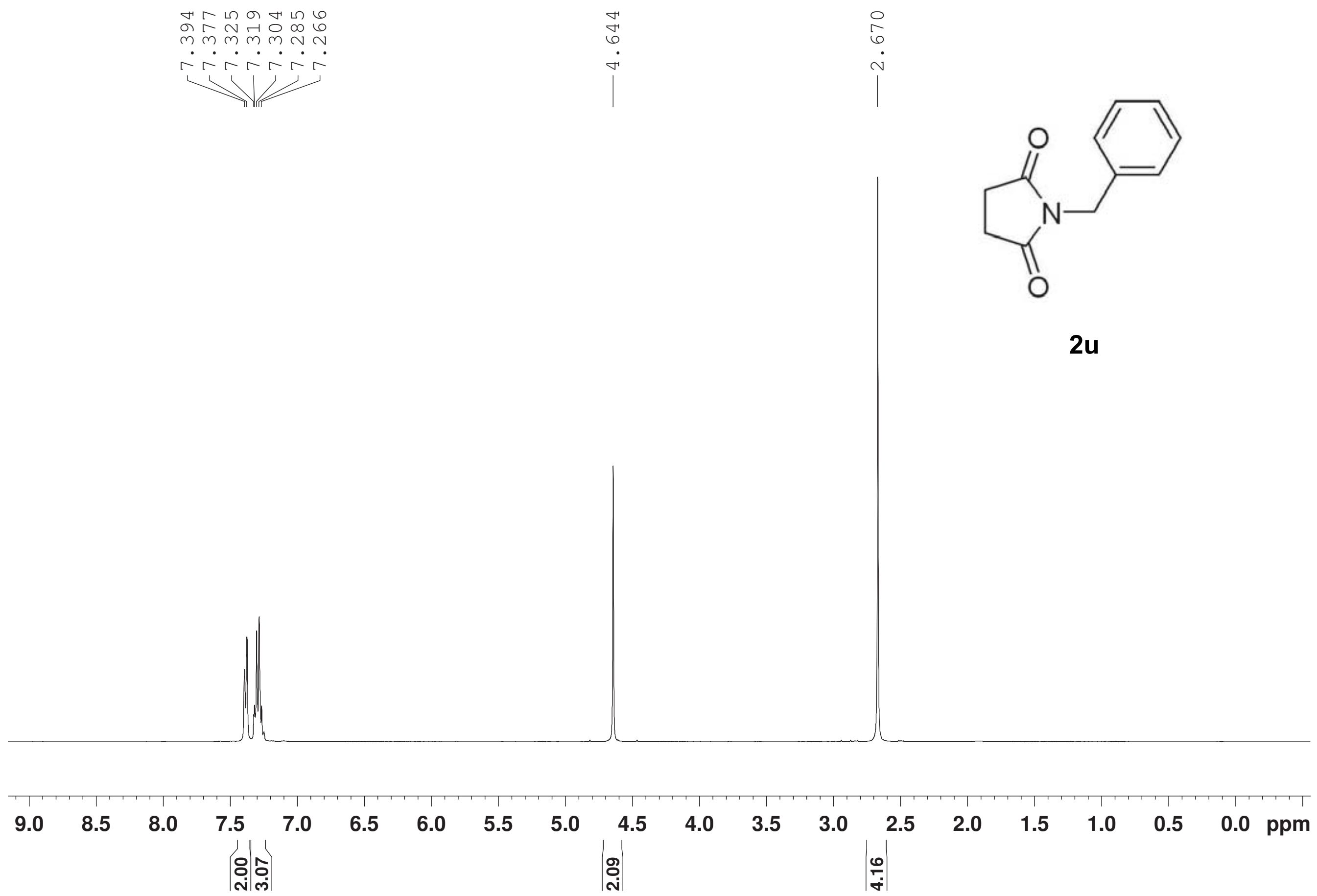
7.394
7.377
7.325
7.319
7.304
7.285
7.266
4.644
2.670
9.0 8.5 8.0 7.5 7.0 6.5 6.0 5.5 5.0 4.5 4.0 3.5 3.0 2.5 2.0 1.5 1.0 0.5 0.0 ppm
2.00
3.07
2.09
4.16

2u

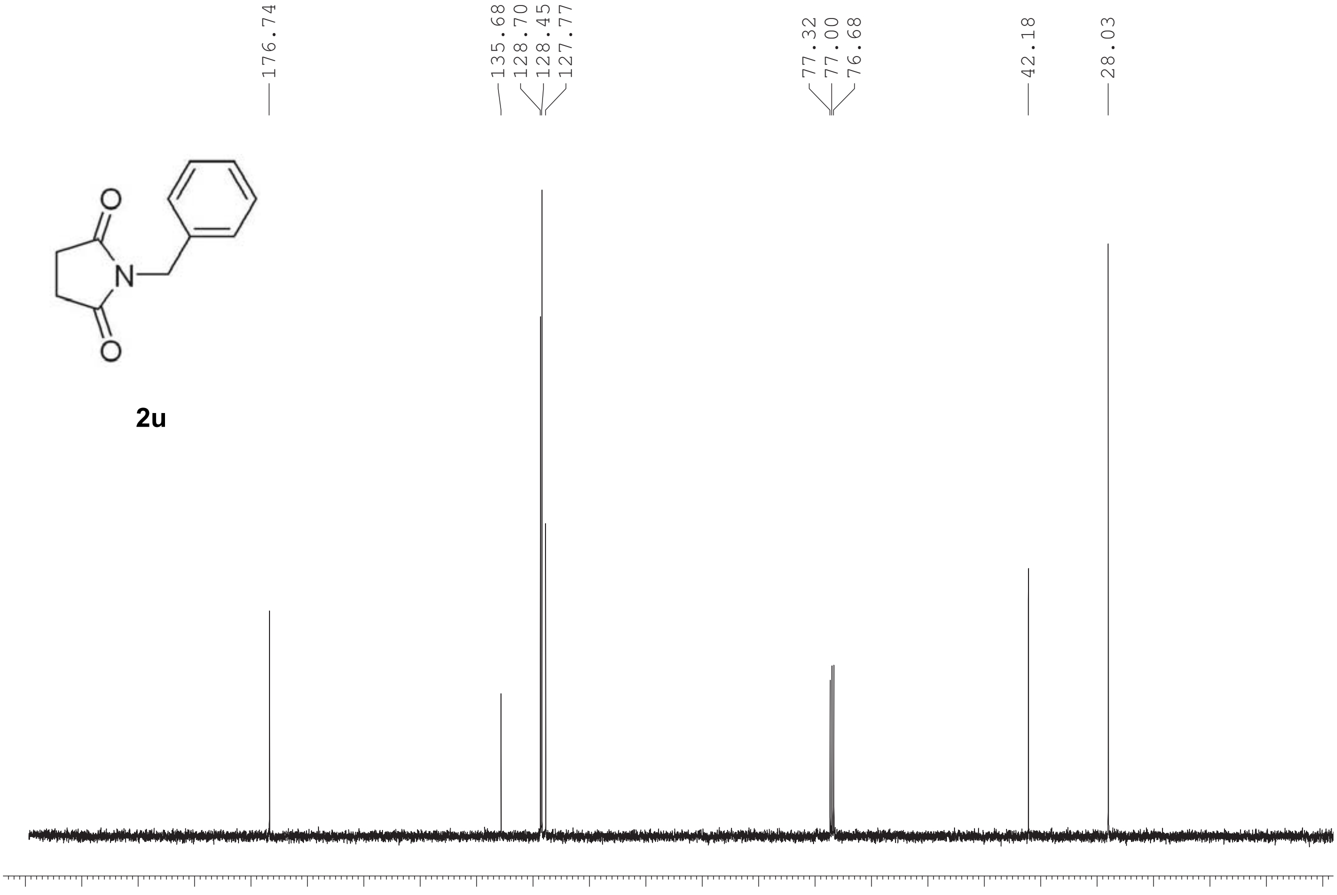
176.74
135.68
128.70
128.45
127.77
77.32
77.00
76.68
42.18
28.03
220 210 200 190 180 170 160 150 140 130 120 110 100 90 80 70 60 50 40 30 20 10 0 ppm

2u

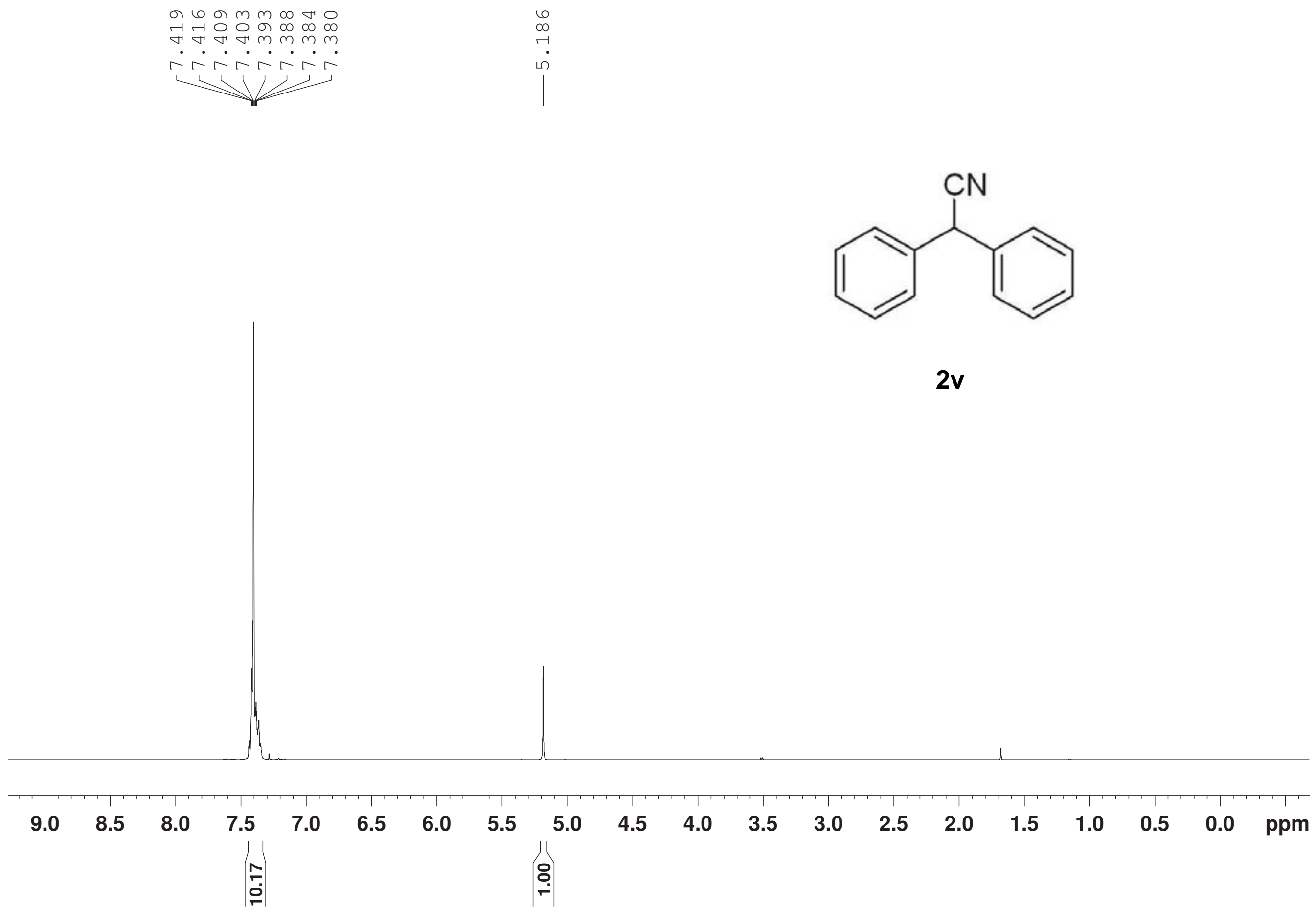
7.419
7.416
7.409
7.403
7.393
7.388
7.384
7.380
5.186
CN
2v
9.0 8.5 8.0 7.5 7.0 6.5 6.0 5.5 5.0 4.5 4.0 3.5 3.0 2.5 2.0 1.5 1.0 0.5 0.0 ppm
10.17
1.00

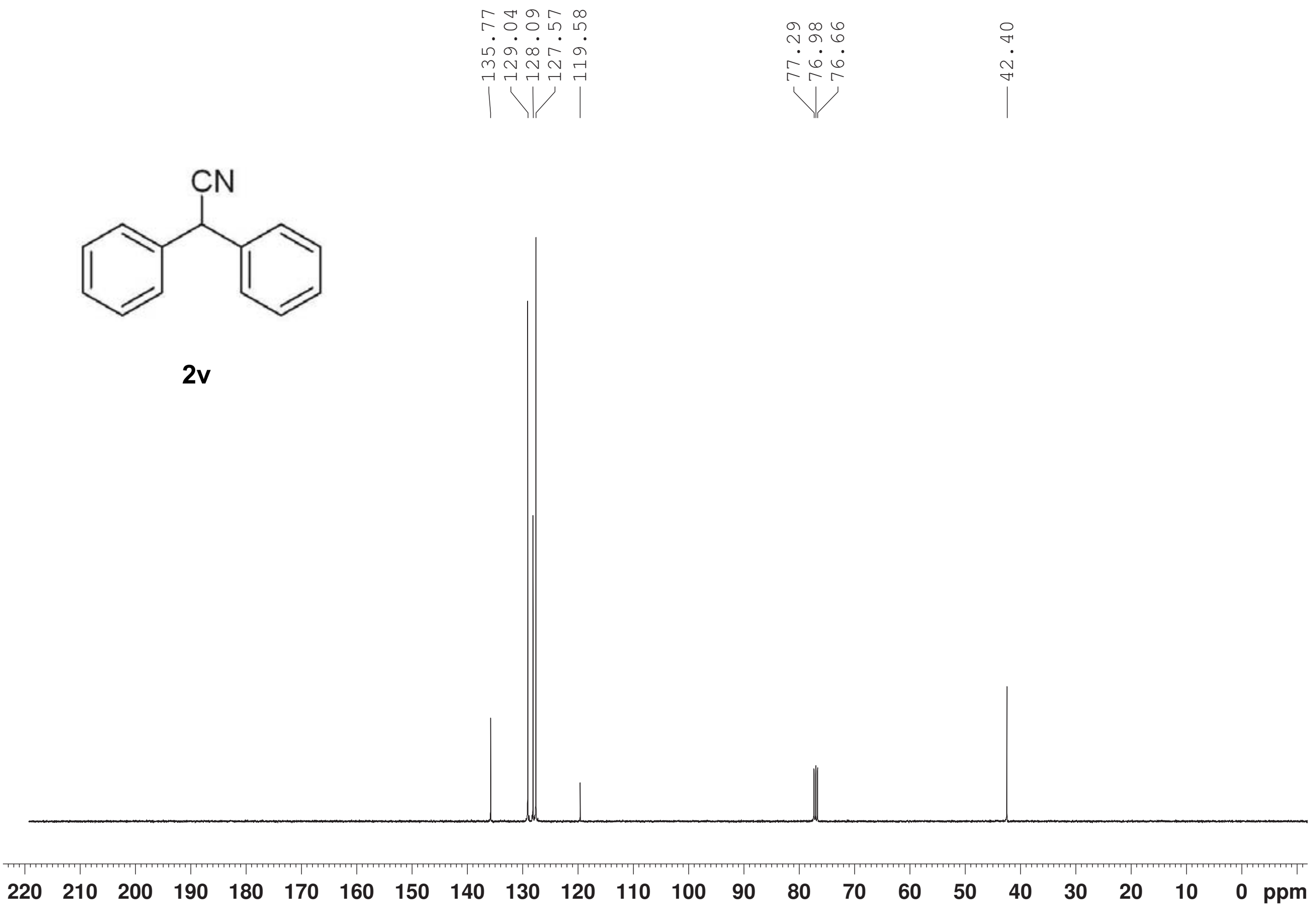
135.77
129.04
128.09
127.57
119.58
77.29
76.98
76.66
42.40
CN
2v
220 210 200 190 180 170 160 150 140 130 120 110 100 90 80 70 60 50 40 30 20 10 0 ppm

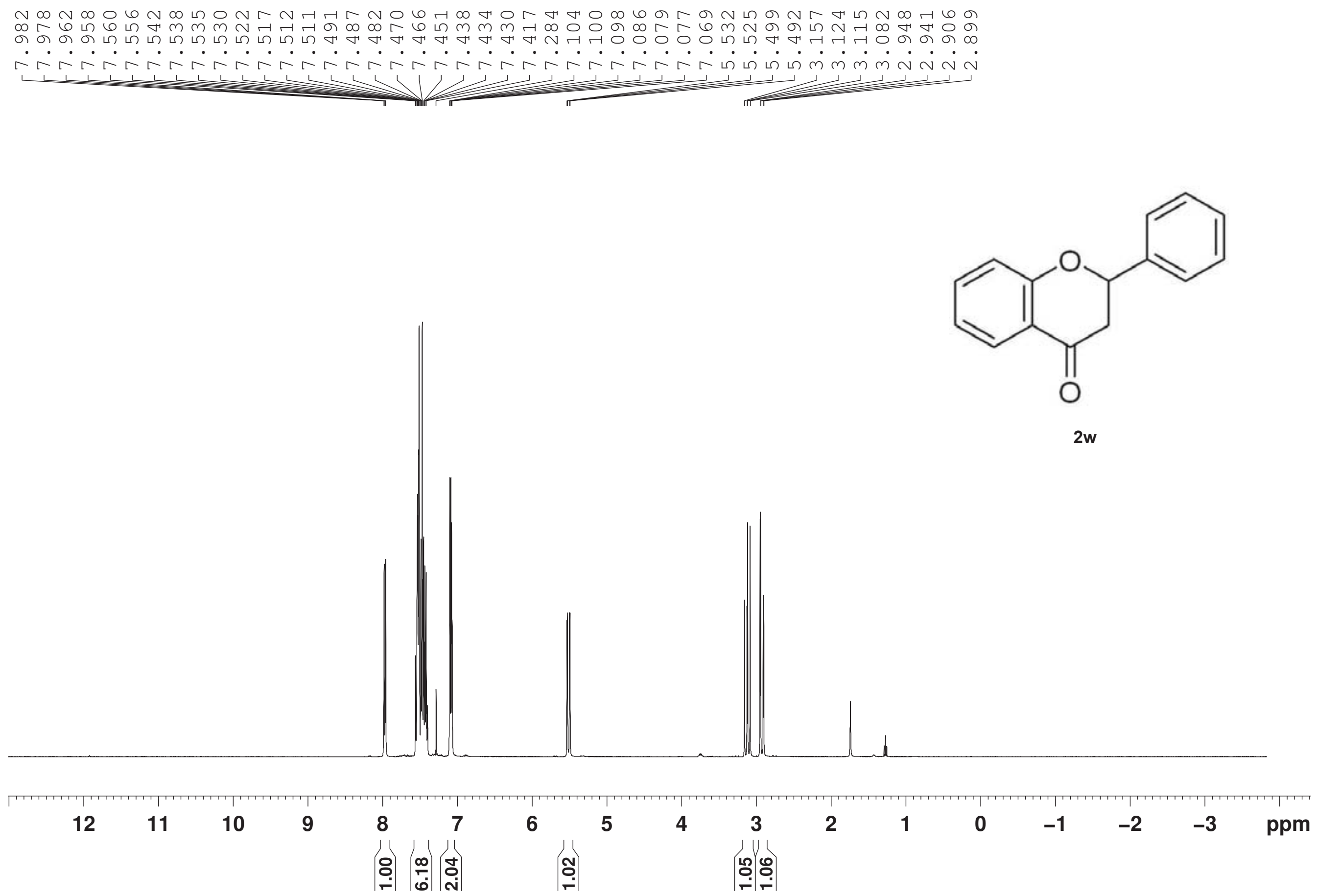
7.982
7.978
7.962
7.958
7.560
7.556
7.542
7.538
7.535
7.530
7.522
7.517
7.512
7.511
7.491
7.487
7.482
7.470
7.466
7.451
7.438
7.434
7.430
7.417
7.284
7.104
7.100
7.098
7.086
7.079
7.077
7.069
5.532
5.525
5.499
5.492
3.157
3.124
3.115
3.082
2.948
2.941
2.906
2.899
2w
12
11
10
9
8
7
6
5
4
3
2
1
0
–1
–2
–3
ppm
1.00
6.18
2.04
1.02
1.05
1.06

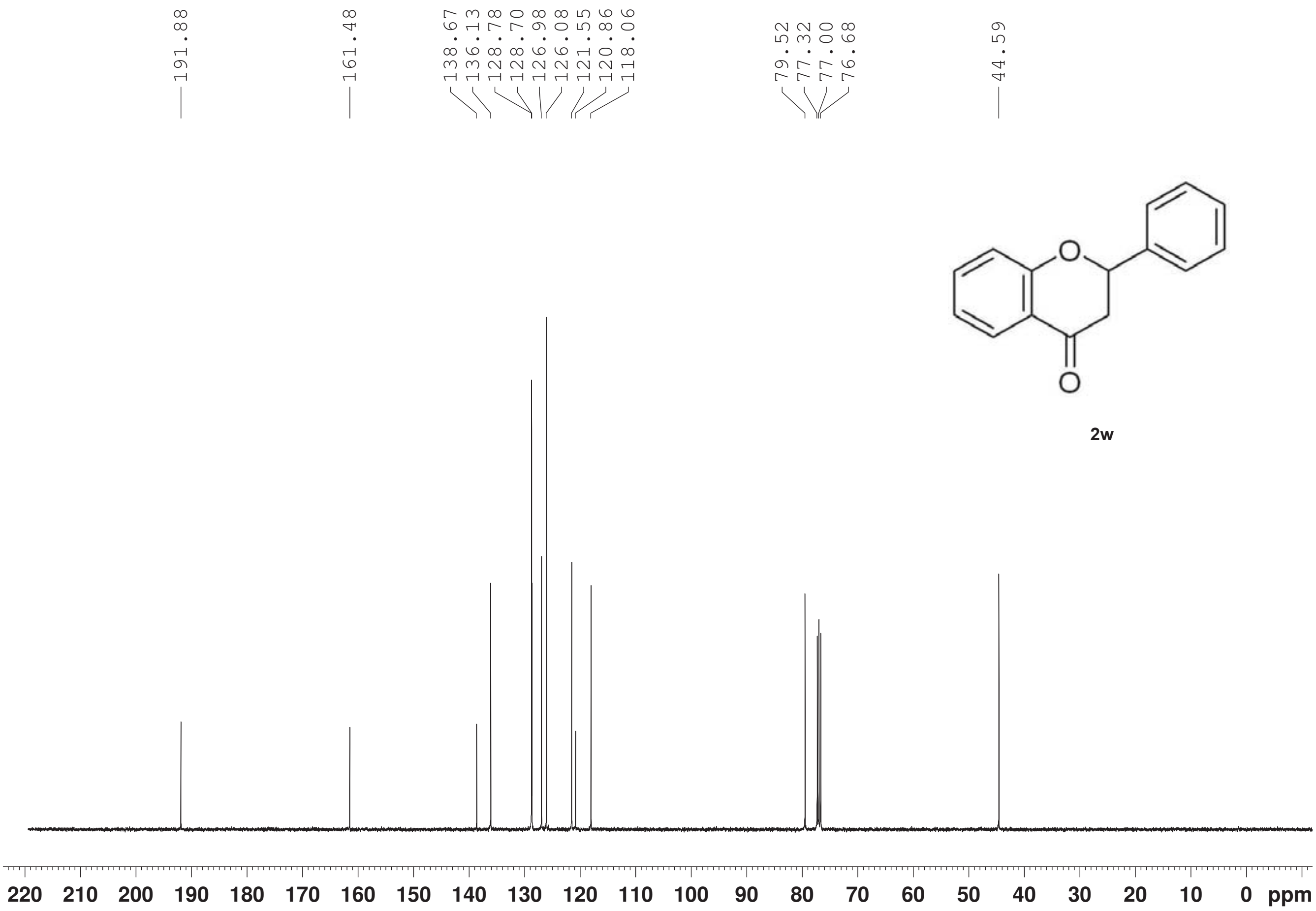
191.88
161.48
138.67
136.13
128.78
128.70
126.98
126.08
121.55
120.86
118.06
79.52
77.32
77.00
76.68
44.59
2w
220
210
200
190
180
170
160
150
140
130
120
110
100
90
80
70
60
50
40
30
20
10
0
ppm

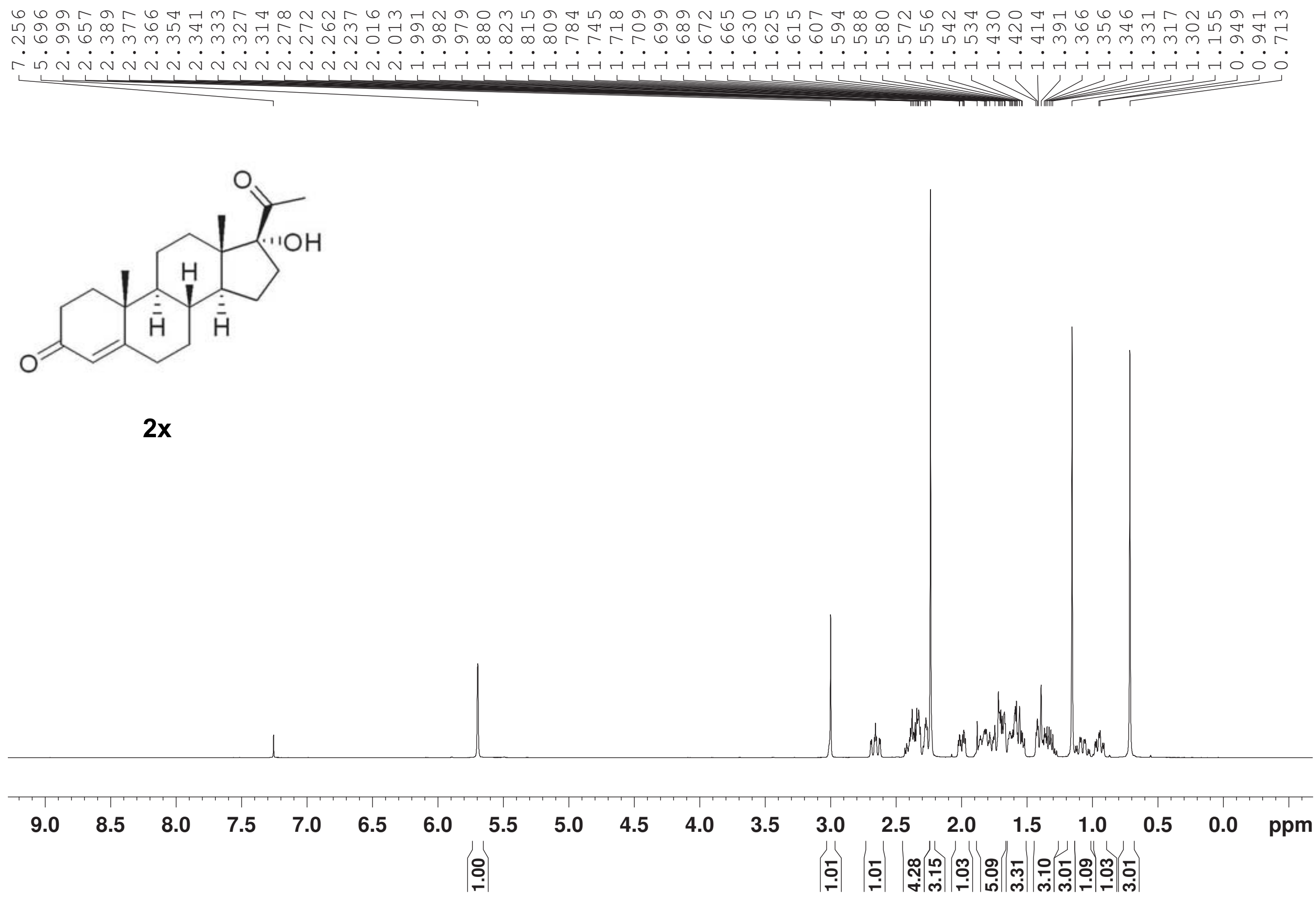
7.256 5.696 2.999 2.657 2.389 2.377 2.366 2.354 2.341 2.333 2.327 2.314 2.278 2.272 2.262 2.237 2.016 2.013 1.991 1.982 1.979 1.880 1.823 1.815 1.809 1.784 1.745 1.718 1.709 1.699 1.689 1.672 1.665 1.630 1.625 1.615 1.607 1.594 1.588 1.580 1.572 1.556 1.542 1.534 1.430 1.420 1.414 1.391 1.366 1.356 1.346 1.331 1.317 1.302 1.155 0.949 0.941 0.713
OH
H
H
H
O
O
2x
9.0 8.5 8.0 7.5 7.0 6.5 6.0 5.5 5.0 4.5 4.0 3.5 3.0 2.5 2.0 1.5 1.0 0.5 0.0 ppm
1.00
1.01 1.01 4.28 3.15 1.03 5.09 3.31 3.10 3.01 1.09 1.03 3.01

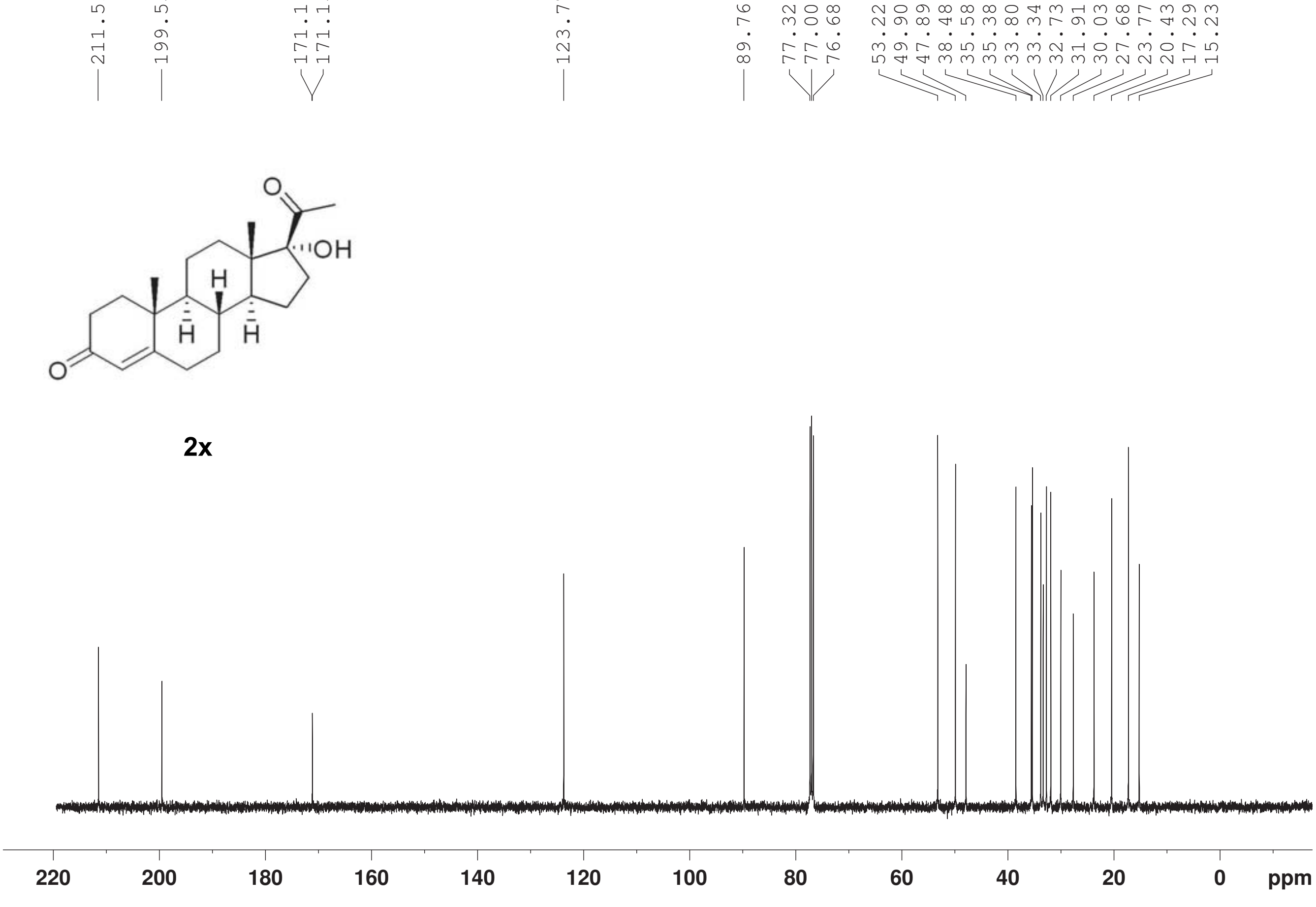
211.51
199.55
171.19 171.15
123.77
89.76
77.32 77.00 76.68
53.22 49.90 47.89 38.48 35.58 35.38 33.80 33.34 32.73 31.91 30.03 27.68 23.77 20.43 17.29 15.23
OH
H
H
H
O
O
2x
220 200 180 160 140 120 100 80 60 40 20 0 ppm
